# Supplementary material for: Purification of cross-linked RNA-protein complexes by phenol-toluol extraction
Source: Nat Commun. 2019 Mar 1;10:990. doi: 10.1038/s41467-019-08942-3 (PMC6397201; doi:10.1038/s41467-019-08942-3)
Supplement: Supplementary file 3 — Description of Additional Supplementary Files [file 41467_2019_8942_MOESM3_ESM.docx]

**Description of Supplementary Files**

**File Name:** Supplementary Data 1

**Description:** PTex HEK293 proteins

**File Name:** Supplementary Data 2

**Description:** PTex HEK293 protein features

**File Name:** Supplementary Data 3

**Description:** PTex Salmonella proteins

**File Name:** Supplementary Data 4

**Description:** PTex performance raw data

**File Name:** Supplementary Data 5

**Description:** PTex HEK293 RNA

**File Name:** Supplementary Data 6

**Description:** PTex HEK293 RNA classes

**File Name:** Supplementary Data 7

**Description:** pCLIP HuR RNA cluster

**File Name:** Supplementary Data 8

**Description:** HEK293 MS Analysis

**File Name:** Supplementary Data 9

**Description:** Salmonella MS Analysis
