# Supplementary material for: Purification of cross-linked RNA-protein complexes by phenol-toluol extraction
Source: Nat Commun. 2019 Mar 1;10:990. doi: 10.1038/s41467-019-08942-3 (PMC6397201; doi:10.1038/s41467-019-08942-3)
Supplement: Supplementary file 11 — Supplementary Data 8 [file 41467_2019_8942_MOESM11_ESM.zip › PTex_Supplementary_HEK293_MS_analysis/PTex.nb.html]

PTex HEK293 MS Analysis


Code 

- Show All Code
- Hide All Code
- Download Rmd

# PTex HEK293 MS Analysis

- 1 Introduction
- 2 Libraries
- 3 Data preparation
  - 3.1 First filter
  - 3.2 Transformation
  - 3.3 Trypsin normalisation factor
  - 3.4 Contaminant removal
  - 3.5 Trypsin normalisation
  - 3.6 Incomplete observation removal
  - 3.7 Plots
    - 3.7.1 Intensities Scatterplots
- 4 Enrichment analysis
  - 4.1 Fold Changes
  - 4.2 Moderated t-test and Benjamini-Hochberg correction
  - 4.3 Mean intensities
  - 4.4 FDR and FC filter subset
  - 4.5 Plots
    - 4.5.1 Enrichments
    - 4.5.2 Volcano Plots
- 5 Matching
  - 5.1 Matching table
    - 5.1.1 Matching with other datasets
  - 5.2 Matching df
- 6 Annotations
  - 6.1 Isoelectric Point, Molecular Weight, Sequences and hydrophobicity
    - 6.1.1 Plots
  - 6.2 RBD
  - 6.3 GO
- 7 Master Table
- 8 Session Info

# 1 Introduction

This document guides through the analysis of the MS data for the PTex HEK293 project. Inputdata have to be provided in the folder `inputdata` inside the working directory. These are:

```
working directory/
  PTex.Rmd
  inputdata/
    proteinGroups.txt                   # MS data
    RBP table-Table 1.csv               # Gerstberger review (2014) RBPs
    human TFs-Table 1.csv               # Gerstberger review (2014) TFs
    2015-06-09_human_interactomes.csv   # Landthaler human mRNA IC
    preiss_genenames.csv                # Preiss
    Brannan_annotated.txt               # Brannan SONAR annotated RBPs
    Brannan_SONAR_0.79.txt              # Brannan SONAR predicted
    hubstenberger.csv                   # Hubstenberger P-Body Proteins
    RICK_high_conf.csv                  # RICK high confidence
    RICK_low_conf.csv                   # RICK low confidence
    RICK_unique.csv                     # RICK unique
    caric.csv                           # CARIC
    human_proteome_reviewed_290118.list # UniProt SwissProt
    UP000005640_9606_all.fasta.pI.csv   # Isoelectricpointdb
```

# 2 Libraries

The following libraries are needed for the analysis. Packages have to be installed prior to loading.


```
library("dplyr")
```


```
Attaching package: ‘dplyr’

The following objects are masked from ‘package:stats’:

    filter, lag

The following objects are masked from ‘package:base’:

    intersect, setdiff, setequal, union
```


```
library("limma")
library("reshape2")
library("ggplot2")
library("GGally")
```


```
Attaching package: ‘GGally’

The following object is masked from ‘package:dplyr’:

    nasa
```


```
library("Peptides")
library("DT")
```


To produce pairwise scatter plots with correlation information we use the `ggpairs()` function from the `GGally` package. Custom theme for pairwise scatter plots:


```
scatter_theme <- theme(legend.position = "none", 
      panel.grid.major = element_blank(), 
      axis.ticks = element_blank(), 
      panel.border = element_rect(linetype = "solid", colour = "black", fill = NA))
ggplot <- function(...) ggplot2::ggplot(...) + scale_colour_manual(values = c("black","red")) + scale_fill_manual(values = c("black","red"))
unlockBinding("ggplot",parent.env(asNamespace("GGally")))
assign("ggplot",ggplot,parent.env(asNamespace("GGally")))
```

# 3 Data preparation

The primary MS data are contained in the file `proteinGroups.txt`.


```
primaryMassSpecData <- read.table("inputdata/proteinGroups.txt", header = TRUE, sep = "\t")
```


To ease later analyses, character vectors of columnnames are created.


```
intensities <- c("Intensity.Input_NoCl_I",
               "Intensity.Input_NoCl_II",
               "Intensity.Input_NoCl_III",
               "Intensity.Input_0015_I",
               "Intensity.Input_0015_II",
               "Intensity.Input_0015_III",
               "Intensity.Input_015_I",
               "Intensity.Input_015_II",
               "Intensity.Input_015_III",
               "Intensity.Input_15_I",
               "Intensity.Input_15_II",
               "Intensity.Input_15_III",
               "Intensity.PTex_NoCl_I",
               "Intensity.PTex_NoCl_II",
               "Intensity.PTex_NoCl_III",
               "Intensity.PTex_0015_I",
               "Intensity.PTex_0015_II",
               "Intensity.PTex_0015_III",
               "Intensity.PTex_015_I",
               "Intensity.PTex_015_II",
               "Intensity.PTex_015_III",
               "Intensity.PTex_15_I",
               "Intensity.PTex_15_II",
               "Intensity.PTex_15_III")
```


```
LFQ <- c("LFQ.intensity.Input_NoCl_I",
               "LFQ.intensity.Input_NoCl_II",
               "LFQ.intensity.Input_NoCl_III",
               "LFQ.intensity.Input_0015_I",
               "LFQ.intensity.Input_0015_II",
               "LFQ.intensity.Input_0015_III",
               "LFQ.intensity.Input_015_I",
               "LFQ.intensity.Input_015_II",
               "LFQ.intensity.Input_015_III",
               "LFQ.intensity.Input_15_I",
               "LFQ.intensity.Input_15_II",
               "LFQ.intensity.Input_15_III",
               "LFQ.intensity.PTex_NoCl_I",
               "LFQ.intensity.PTex_NoCl_II",
               "LFQ.intensity.PTex_NoCl_III",
               "LFQ.intensity.PTex_0015_I",
               "LFQ.intensity.PTex_0015_II",
               "LFQ.intensity.PTex_0015_III",
               "LFQ.intensity.PTex_015_I",
               "LFQ.intensity.PTex_015_II",
               "LFQ.intensity.PTex_015_III",
               "LFQ.intensity.PTex_15_I",
               "LFQ.intensity.PTex_15_II",
               "LFQ.intensity.PTex_15_III")
```


```
IBAQ <- c("iBAQ.Input_NoCl_I",
               "iBAQ.Input_NoCl_II",
               "iBAQ.Input_NoCl_III",
               "iBAQ.Input_0015_I",
               "iBAQ.Input_0015_II",
               "iBAQ.Input_0015_III",
               "iBAQ.Input_015_I",
               "iBAQ.Input_015_II",
               "iBAQ.Input_015_III",
               "iBAQ.Input_15_I",
               "iBAQ.Input_15_II",
               "iBAQ.Input_15_III",
               "iBAQ.PTex_NoCl_I",
               "iBAQ.PTex_NoCl_II",
               "iBAQ.PTex_NoCl_III",
               "iBAQ.PTex_0015_I",
               "iBAQ.PTex_0015_II",
               "iBAQ.PTex_0015_III",
               "iBAQ.PTex_015_I",
               "iBAQ.PTex_015_II",
               "iBAQ.PTex_015_III",
               "iBAQ.PTex_15_I",
               "iBAQ.PTex_15_II",
               "iBAQ.PTex_15_III")
```


## 3.1 First filter

In a first filtering step reverse and hits only identified by site are removed.


```
MassSpecData <- primaryMassSpecData[primaryMassSpecData$Reverse != "+",]
MassSpecData <- MassSpecData[MassSpecData$Only.identified.by.site != "+",]
```

## 3.2 Transformation

All intensites are log2 transformed.


```
MassSpecData[c(intensities, IBAQ, LFQ)] <- log2(MassSpecData[c(intensities, IBAQ, LFQ)])
```


Infinity values that resulted from log2 transformation are converted to `NA`:


```
is.na(MassSpecData[c(intensities,IBAQ,LFQ)]) <- sapply(MassSpecData[c(intensities,IBAQ,LFQ)], is.infinite)
```

## 3.3 Trypsin normalisation factor

The LFQ intensities are normalised under the assumption that most intensities won’t change. This is not true for PTex data, so that the normalisation has to be corrected. Correction is done by using trypsin (added to all samples in the same amount) to calculate a normalisation factor.


```
trypsin = MassSpecData[MassSpecData$Majority.protein.IDs == "CON__P00761",]
trp_mean_Inp_NoCl <- mean(trypsin[,LFQ[1]],trypsin[,LFQ[2]],trypsin[,LFQ[3]])
trp_mean_Inp_0015 <- mean(trypsin[,LFQ[4]],trypsin[,LFQ[5]],trypsin[,LFQ[6]])
trp_mean_Inp_015 <- mean(trypsin[,LFQ[7]],trypsin[,LFQ[8]],trypsin[,LFQ[9]])
trp_mean_Inp_15 <- mean(trypsin[,LFQ[10]],trypsin[,LFQ[11]],trypsin[,LFQ[12]])
trp_mean_PTex_NoCl <- mean(trypsin[,LFQ[13]],trypsin[,LFQ[14]],trypsin[,LFQ[15]])
trp_mean_PTex_0015 <- mean(trypsin[,LFQ[16]],trypsin[,LFQ[17]],trypsin[,LFQ[18]])
trp_mean_PTex_015 <- mean(trypsin[,LFQ[19]],trypsin[,LFQ[20]],trypsin[,LFQ[21]])
trp_mean_PTex_15 <- mean(trypsin[,LFQ[22]],trypsin[,LFQ[23]],trypsin[,LFQ[24]]) 
nf_NoCl <- trp_mean_PTex_NoCl - trp_mean_Inp_NoCl
nf_0015Cl <- trp_mean_PTex_0015 - trp_mean_Inp_0015
nf_015Cl <- trp_mean_PTex_015 - trp_mean_Inp_015
nf_15Cl <- trp_mean_PTex_15 - trp_mean_Inp_15
```


Tidy up:


```
rm(list = ls(pattern = "trp_mean_"))
rm(trypsin)
```

## 3.4 Contaminant removal

After calculation of the normalisation factors the contaminants are removed from the data set.


```
MassSpecData <- MassSpecData[MassSpecData$Potential.contaminant != "+",]
```

## 3.5 Trypsin normalisation

The normalisation factors are applied by subtraction, since the data were already log transformed.


```
norm_MassSpecData <- MassSpecData
norm_MassSpecData[LFQ[13:15]] <- norm_MassSpecData[LFQ[13:15]] - nf_NoCl
norm_MassSpecData[LFQ[16:18]] <- norm_MassSpecData[LFQ[16:18]] - nf_0015Cl
norm_MassSpecData[LFQ[19:21]] <- norm_MassSpecData[LFQ[19:21]] - nf_015Cl
norm_MassSpecData[LFQ[22:24]] <- norm_MassSpecData[LFQ[22:24]] - nf_15Cl
```


Tidy up:


```
rm(list = ls(pattern = "nf_"))
```

## 3.6 Incomplete observation removal

We only want to consider those proteins, that were found in all replicates of all experiments.


```
for(exp in c("PTex_noCL","PTex_0015","PTex_015","PTex_15", "Input_noCL","Input_0015","Input_015","Input_15")){
  
  sub_MassSpecData <- select(norm_MassSpecData, matches(paste("LFQ.intensity", exp, sep = ".")))
  x <- apply(sub_MassSpecData, 1, function(x)sum(!is.na(x))>2)
  norm_MassSpecData <- norm_MassSpecData[x,]
}
```


Tidy up:


```
rm(list = ls(pattern = "sub_"))
rm(exp)
rm(x)
```

## 3.7 Plots

### 3.7.1 Intensities Scatterplots


```
## Input noCL
input_nocl_scatter <- ggpairs(norm_MassSpecData[,LFQ[1:3]], 
        lower = list(continuous = wrap("cor", size = 7)), 
        upper = list(continuous = "smooth"),
        diag = list(continuous = "densityDiag")) + scatter_theme
## Input 0.015
input_0015_scatter <- ggpairs(norm_MassSpecData[,LFQ[4:6]], 
        lower = list(continuous = wrap("cor", size = 7)), 
        upper = list(continuous = "smooth"),
        diag = list(continuous = "densityDiag")) + scatter_theme
## Input 0.15
input_015_scatter <- ggpairs(norm_MassSpecData[,LFQ[7:9]], 
        lower = list(continuous = wrap("cor", size = 7)), 
        upper = list(continuous = "smooth"),
        diag = list(continuous = "densityDiag"))  + scatter_theme
## Input 1.5
input_15_scatter <- ggpairs(norm_MassSpecData[,LFQ[10:12]], 
        lower = list(continuous = wrap("cor", size = 7)), 
        upper = list(continuous = "smooth"),
        diag = list(continuous = "densityDiag"))  + scatter_theme
## PTex noCL
ptex_nocl_scatter <- ggpairs(norm_MassSpecData[,LFQ[13:15]], 
        lower = list(continuous = wrap("cor", size = 7)), 
        upper = list(continuous = "smooth"),
        diag = list(continuous = "densityDiag"))  + scatter_theme
## PTex 0.015
ptex_0015_scatter <- ggpairs(norm_MassSpecData[,LFQ[16:18]], 
        lower = list(continuous = wrap("cor", size = 7)), 
        upper = list(continuous = "smooth"),
        diag = list(continuous = "densityDiag"))  + scatter_theme
## PTex 0.15
ptex_015_scatter <- ggpairs(norm_MassSpecData[,LFQ[19:21]], 
        lower = list(continuous = wrap("cor", size = 7)), 
        upper = list(continuous = "smooth"),
        diag = list(continuous = "densityDiag"))  + scatter_theme
## PTex 1.5
ptex_15_scatter <- ggpairs(norm_MassSpecData[,LFQ[22:24]],
        lower = list(continuous = wrap("cor", size = 7)), 
        upper = list(continuous = "smooth"),
        diag = list(continuous = "densityDiag"))  + scatter_theme
```


```
input_nocl_scatter
```


```
input_0015_scatter
```


```
input_015_scatter
```


```
input_15_scatter
```


```
ptex_nocl_scatter
```


```
ptex_0015_scatter
```


```
ptex_015_scatter
```


```
ptex_15_scatter
```


Tidy up:


```
rm(list = ls(pattern = "_scatter"))
```

# 4 Enrichment analysis

After preparation the fold changes between respective Input/PTex pairs can be calculated and a moderated t-test with following Benjamini-Hochberg correction is used to determine the false discovery rate (FDR).

## 4.1 Fold Changes

The fold changes are calculated by subtracting the log-transformed LFQ intensity values of the non-crosslinked (-CL) from the crosslinked (+CL) samples.


```
norm_MassSpecData$FC.Input_0015.rep1 <- (norm_MassSpecData$LFQ.intensity.Input_0015_I - norm_MassSpecData$LFQ.intensity.Input_NoCl_I)
norm_MassSpecData$FC.Input_0015.rep2 <- (norm_MassSpecData$LFQ.intensity.Input_0015_II - norm_MassSpecData$LFQ.intensity.Input_NoCl_II)
norm_MassSpecData$FC.Input_0015.rep3 <- (norm_MassSpecData$LFQ.intensity.Input_0015_III - norm_MassSpecData$LFQ.intensity.Input_NoCl_III)
norm_MassSpecData$FC.Input_015.rep1 <- (norm_MassSpecData$LFQ.intensity.Input_015_I - norm_MassSpecData$LFQ.intensity.Input_NoCl_I)
norm_MassSpecData$FC.Input_015.rep2 <- (norm_MassSpecData$LFQ.intensity.Input_015_II - norm_MassSpecData$LFQ.intensity.Input_NoCl_II)
norm_MassSpecData$FC.Input_015.rep3 <- (norm_MassSpecData$LFQ.intensity.Input_015_III - norm_MassSpecData$LFQ.intensity.Input_NoCl_III)
norm_MassSpecData$FC.Input_15.rep1 <- (norm_MassSpecData$LFQ.intensity.Input_15_I - norm_MassSpecData$LFQ.intensity.Input_NoCl_I)
norm_MassSpecData$FC.Input_15.rep2 <- (norm_MassSpecData$LFQ.intensity.Input_15_II - norm_MassSpecData$LFQ.intensity.Input_NoCl_II)
norm_MassSpecData$FC.Input_15.rep3 <- (norm_MassSpecData$LFQ.intensity.Input_15_III - norm_MassSpecData$LFQ.intensity.Input_NoCl_III)
norm_MassSpecData$FC.PTex_0015.rep1 <- (norm_MassSpecData$LFQ.intensity.PTex_0015_I - norm_MassSpecData$LFQ.intensity.PTex_NoCl_I)
norm_MassSpecData$FC.PTex_0015.rep2 <- (norm_MassSpecData$LFQ.intensity.PTex_0015_II - norm_MassSpecData$LFQ.intensity.PTex_NoCl_II)
norm_MassSpecData$FC.PTex_0015.rep3 <- (norm_MassSpecData$LFQ.intensity.PTex_0015_III - norm_MassSpecData$LFQ.intensity.PTex_NoCl_III)
norm_MassSpecData$FC.PTex_015.rep1 <- (norm_MassSpecData$LFQ.intensity.PTex_015_I - norm_MassSpecData$LFQ.intensity.PTex_NoCl_I)
norm_MassSpecData$FC.PTex_015.rep2 <- (norm_MassSpecData$LFQ.intensity.PTex_015_II - norm_MassSpecData$LFQ.intensity.PTex_NoCl_II)
norm_MassSpecData$FC.PTex_015.rep3 <- (norm_MassSpecData$LFQ.intensity.PTex_015_III - norm_MassSpecData$LFQ.intensity.PTex_NoCl_III)
norm_MassSpecData$FC.PTex_15.rep1 <- (norm_MassSpecData$LFQ.intensity.PTex_15_I - norm_MassSpecData$LFQ.intensity.PTex_NoCl_I)
norm_MassSpecData$FC.PTex_15.rep2 <- (norm_MassSpecData$LFQ.intensity.PTex_15_II - norm_MassSpecData$LFQ.intensity.PTex_NoCl_II)
norm_MassSpecData$FC.PTex_15.rep3 <- (norm_MassSpecData$LFQ.intensity.PTex_15_III - norm_MassSpecData$LFQ.intensity.PTex_NoCl_III)
```


Mean fold changes are calculated.


```
norm_MassSpecData$FC.Input_0015.mean <- rowMeans(norm_MassSpecData[,251:253])
norm_MassSpecData$FC.Input_015.mean <- rowMeans(norm_MassSpecData[,254:256])
norm_MassSpecData$FC.Input_15.mean <- rowMeans(norm_MassSpecData[,257:259])
norm_MassSpecData$FC.PTex_0015.mean <- rowMeans(norm_MassSpecData[,260:262])
norm_MassSpecData$FC.PTex_015.mean <- rowMeans(norm_MassSpecData[,263:265])
norm_MassSpecData$FC.PTex_15.mean <- rowMeans(norm_MassSpecData[,266:268])
```

## 4.2 Moderated t-test and Benjamini-Hochberg correction

A moderated t-test including Benjamini-Hochberg p-value correction is layed out on the fold changes.


```
pval =  eBayes(lmFit(norm_MassSpecData[,grep("FC.Input_0015.rep",names(norm_MassSpecData))]))
norm_MassSpecData$pval.Input_0015 <- pval$p.value
norm_MassSpecData$padj.Input_0015 <-  p.adjust(norm_MassSpecData$pval.Input_0015, method="BH")
pval =  eBayes(lmFit(norm_MassSpecData[,grep("FC.Input_015.rep",names(norm_MassSpecData))]))
norm_MassSpecData$pval.Input_015 <- pval$p.value
norm_MassSpecData$padj.Input_015 <-  p.adjust(norm_MassSpecData$pval.Input_015, method="BH")
pval =  eBayes(lmFit(norm_MassSpecData[,grep("FC.Input_15.rep",names(norm_MassSpecData))]))
norm_MassSpecData$pval.Input_15 <- pval$p.value
norm_MassSpecData$padj.Input_15 <-  p.adjust(norm_MassSpecData$pval.Input_15, method="BH")
pval = eBayes(lmFit(norm_MassSpecData[,grep("FC.PTex_0015.rep",names(norm_MassSpecData))]))
norm_MassSpecData$pval.PTex_0015 <- pval$p.value
norm_MassSpecData$padj.PTex_0015 <- p.adjust(norm_MassSpecData$pval.PTex_0015, method="BH")
pval = eBayes(lmFit(norm_MassSpecData[,grep("FC.PTex_015.rep",names(norm_MassSpecData))]))
norm_MassSpecData$pval.PTex_015 <- pval$p.value
norm_MassSpecData$padj.PTex_015 <- p.adjust(norm_MassSpecData$pval.PTex_015, method="BH")
pval = eBayes(lmFit(norm_MassSpecData[,grep("FC.PTex_15.rep",names(norm_MassSpecData))]))
norm_MassSpecData$pval.PTex_15 <- pval$p.value
norm_MassSpecData$padj.PTex_15 <- p.adjust(norm_MassSpecData$pval.PTex_15, method="BH")
```


Tidy up:


```
rm(pval)
```

## 4.3 Mean intensities

Mean intensities (LFQ and iBAQ) are calculated.


```
### LFQ
norm_MassSpecData$LFQ.intensity.Input_noCl_mean <- rowMeans(norm_MassSpecData[,c("LFQ.intensity.Input_NoCl_I", "LFQ.intensity.Input_NoCl_II", "LFQ.intensity.Input_NoCl_III")], na.rm = FALSE)
norm_MassSpecData$LFQ.intensity.Input_0015_mean <- rowMeans(norm_MassSpecData[,c("LFQ.intensity.Input_0015_I", "LFQ.intensity.Input_0015_II", "LFQ.intensity.Input_0015_III")], na.rm = FALSE)
norm_MassSpecData$LFQ.intensity.Input_015_mean <- rowMeans(norm_MassSpecData[,c("LFQ.intensity.Input_015_I", "LFQ.intensity.Input_015_II", "LFQ.intensity.Input_015_III")], na.rm = FALSE)
norm_MassSpecData$LFQ.intensity.Input_15_mean <- rowMeans(norm_MassSpecData[,c("LFQ.intensity.Input_15_I", "LFQ.intensity.Input_15_II", "LFQ.intensity.Input_15_III")], na.rm = FALSE)
norm_MassSpecData$LFQ.intensity.PTex_noCl_mean <- rowMeans(norm_MassSpecData[,c("LFQ.intensity.PTex_NoCl_I", "LFQ.intensity.PTex_NoCl_II", "LFQ.intensity.PTex_NoCl_III")], na.rm = FALSE)
norm_MassSpecData$LFQ.intensity.PTex_0015_mean <- rowMeans(norm_MassSpecData[,c("LFQ.intensity.PTex_0015_I", "LFQ.intensity.PTex_0015_II", "LFQ.intensity.PTex_0015_III")], na.rm = FALSE)
norm_MassSpecData$LFQ.intensity.PTex_015_mean <- rowMeans(norm_MassSpecData[,c("LFQ.intensity.PTex_015_I", "LFQ.intensity.PTex_015_II", "LFQ.intensity.PTex_015_III")], na.rm = FALSE)
norm_MassSpecData$LFQ.intensity.PTex_15_mean <- rowMeans(norm_MassSpecData[,c("LFQ.intensity.PTex_15_I", "LFQ.intensity.PTex_15_II", "LFQ.intensity.PTex_15_III")], na.rm = FALSE)
### iBAQ
norm_MassSpecData$iBAQ.Input_noCl_mean <- rowMeans(norm_MassSpecData[,c("iBAQ.Input_NoCl_I", "iBAQ.Input_NoCl_II", "iBAQ.Input_NoCl_III")], na.rm = FALSE)
norm_MassSpecData$iBAQ.Input_0015_mean <- rowMeans(norm_MassSpecData[,c("iBAQ.Input_0015_I", "iBAQ.Input_0015_II", "iBAQ.Input_0015_III")], na.rm = FALSE)
norm_MassSpecData$iBAQ.Input_015_mean <- rowMeans(norm_MassSpecData[,c("iBAQ.Input_015_I", "iBAQ.Input_015_II", "iBAQ.Input_015_III")], na.rm = FALSE)
norm_MassSpecData$iBAQ.Input_15_mean <- rowMeans(norm_MassSpecData[,c("iBAQ.Input_15_I", "iBAQ.Input_15_II", "iBAQ.Input_15_III")], na.rm = FALSE)
norm_MassSpecData$iBAQ.PTex_noCl_mean <- rowMeans(norm_MassSpecData[,c("iBAQ.PTex_NoCl_I", "iBAQ.PTex_NoCl_II", "iBAQ.PTex_NoCl_III")], na.rm = FALSE)
norm_MassSpecData$iBAQ.PTex_0015_mean <- rowMeans(norm_MassSpecData[,c("iBAQ.PTex_0015_I", "iBAQ.PTex_0015_II", "iBAQ.PTex_0015_III")], na.rm = FALSE)
norm_MassSpecData$iBAQ.PTex_015_mean <- rowMeans(norm_MassSpecData[,c("iBAQ.PTex_015_I", "iBAQ.PTex_015_II", "iBAQ.PTex_015_III")], na.rm = FALSE)
norm_MassSpecData$iBAQ.PTex_15_mean <- rowMeans(norm_MassSpecData[,c("iBAQ.PTex_15_I", "iBAQ.PTex_15_II", "iBAQ.PTex_15_III")], na.rm = FALSE)
```

## 4.4 FDR and FC filter subset

We want to additionally exclude proteins that are not significantly enriched in all 3 libraries and those, which are significantly depleted:


```
sig_norm_MassSpecData <- norm_MassSpecData[norm_MassSpecData$padj.PTex_0015 <= 0.01 & norm_MassSpecData$padj.PTex_015 <= 0.01 & norm_MassSpecData$padj.PTex_15 <= 0.01,]
dim(sig_norm_MassSpecData)
```


```
[1] 3042  302
```


```
sig_norm_MassSpecData <- sig_norm_MassSpecData[sig_norm_MassSpecData$FC.PTex_0015.mean > 0 & sig_norm_MassSpecData$FC.PTex_015.mean > 0 & sig_norm_MassSpecData$FC.PTex_15.mean > 0,]
dim(sig_norm_MassSpecData)
```


```
[1] 3037  302
```

## 4.5 Plots

### 4.5.1 Enrichments


```
FC <- colnames(norm_MassSpecData)[251:268]
## Input 0.015
padj_co <- norm_MassSpecData$padj.Input_0015 <= 0.01
padj_co[which(padj_co)] <- "significant"
padj_co[which(padj_co==FALSE)] <- "non-significant"
fc_input_0015_scatter <- ggpairs(cbind(norm_MassSpecData[,FC[1:3]], padj_co),
        columns = 1:3,
        mapping = ggplot2::aes(colour = padj_co, alpha = 0.5),
        lower = list(continuous = wrap("cor", size = 5)), 
        upper = list(continuous = "smooth"),
        diag = list(continuous = "densityDiag")) + scatter_theme
## Input 0.15
padj_co <- norm_MassSpecData$padj.Input_015 <= 0.01
padj_co[which(padj_co)] <- "significant"
padj_co[which(padj_co==FALSE)] <- "non-significant"
fc_input_015_scatter <- ggpairs(cbind(norm_MassSpecData[,FC[4:6]], padj_co),
        columns = 1:3,
        mapping = ggplot2::aes(colour = padj_co, alpha = 0.5),
        lower = list(continuous = wrap("cor", size = 5)), 
        upper = list(continuous = "smooth"),
        diag = list(continuous = "densityDiag"))  + scatter_theme
## Input 1.5
padj_co <- norm_MassSpecData$padj.Input_15 <= 0.01
padj_co[which(padj_co)] <- "significant"
padj_co[which(padj_co==FALSE)] <- "non-significant"
fc_input_15_scatter <- ggpairs(cbind(norm_MassSpecData[,FC[7:9]], padj_co),
        columns = 1:3,
        mapping = ggplot2::aes(colour = padj_co, alpha = 0.5), 
        lower = list(continuous = wrap("cor", size = 5)), 
        upper = list(continuous = "smooth"),
        diag = list(continuous = "densityDiag"))  + scatter_theme
## PTex 0.015
padj_co <- norm_MassSpecData$padj.PTex_0015 <= 0.01
padj_co[which(padj_co)] <- "significant"
padj_co[which(padj_co==FALSE)] <- "non-significant"
fc_ptex_0015_scatter <- ggpairs(cbind(norm_MassSpecData[,FC[10:12]], padj_co),
        columns = 1:3,
        mapping = ggplot2::aes(colour = padj_co, alpha = 0.5), 
        lower = list(continuous = wrap("cor", size = 5)), 
        upper = list(continuous = "smooth"),
        diag = list(continuous = "densityDiag"))  + scatter_theme
## PTex 0.15
padj_co <- norm_MassSpecData$padj.PTex_015 <= 0.01
padj_co[which(padj_co)] <- "significant"
padj_co[which(padj_co==FALSE)] <- "non-significant"
fc_ptex_015_scatter <- ggpairs(cbind(norm_MassSpecData[,FC[13:15]], padj_co),
        columns = 1:3,
        mapping = ggplot2::aes(colour = padj_co, alpha = 0.5),
        lower = list(continuous = wrap("cor", size = 5)), 
        upper = list(continuous = "smooth"),
        diag = list(continuous = "densityDiag"))  + scatter_theme
## PTex 1.5
padj_co <- norm_MassSpecData$padj.PTex_15 <= 0.01
padj_co[which(padj_co)] <- "significant"
padj_co[which(padj_co==FALSE)] <- "non-significant"
fc_ptex_15_scatter <- ggpairs(cbind(norm_MassSpecData[,FC[16:18]], padj_co),
        columns = 1:3,
        mapping = ggplot2::aes(colour = padj_co, alpha = 0.5), 
        lower = list(continuous = wrap("cor", size = 5)), 
        upper = list(continuous = "smooth"),
        diag = list(continuous = "densityDiag"))  + scatter_theme
```


```
fc_input_0015_scatter
```


```
fc_input_015_scatter
```


```
fc_input_15_scatter
```


```
fc_ptex_0015_scatter
```


```
fc_ptex_015_scatter
```


```
fc_ptex_15_scatter
```

### 4.5.2 Volcano Plots


```
## Input 0.015
padj_co <- norm_MassSpecData$padj.Input_0015 <= 0.01
padj_co[which(padj_co)] <- "significant"
padj_co[which(padj_co==FALSE)] <- "non-significant"
fc_input_0015_volcano <- ggplot(data=cbind(norm_MassSpecData, padj_co), aes(x=FC.Input_0015.mean, y=-log10(padj.Input_0015), colour = padj_co ) ) +
  geom_point(alpha=0.5, size=1.75) +
#  xlim(c(-6, 6)) + ylim(c(0, 10)) +
  xlab("log2 fold change") + ylab("-log10 adj. p-value")
## Input 0.15
padj_co <- norm_MassSpecData$padj.Input_015 <= 0.01
padj_co[which(padj_co)] <- "significant"
padj_co[which(padj_co==FALSE)] <- "non-significant"
fc_input_015_volcano <- ggplot(data=cbind(norm_MassSpecData, padj_co), aes(x=FC.Input_015.mean, y=-log10(padj.Input_015), colour = padj_co ) ) +
  geom_point(alpha=0.5, size=1.75) +
#  xlim(c(-6, 6)) + ylim(c(0, 10)) +
  xlab("log2 fold change") + ylab("-log10 adj. p-value")
## Input 1.5
padj_co <- norm_MassSpecData$padj.Input_15 <= 0.01
padj_co[which(padj_co)] <- "significant"
padj_co[which(padj_co==FALSE)] <- "non-significant"
fc_input_15_volcano <- ggplot(data=cbind(norm_MassSpecData, padj_co), aes(x=FC.Input_15.mean, y=-log10(padj.Input_15), colour = padj_co ) ) +
  geom_point(alpha=0.5, size=1.75) +
#  xlim(c(-6, 6)) + ylim(c(0, 10)) +
  xlab("log2 fold change") + ylab("-log10 adj. p-value")
## PTex 0.015
padj_co <- norm_MassSpecData$padj.PTex_0015 <= 0.01
padj_co[which(padj_co)] <- "significant"
padj_co[which(padj_co==FALSE)] <- "non-significant"
fc_ptex_0015_volcano <- ggplot(data=cbind(norm_MassSpecData, padj_co), aes(x=FC.PTex_0015.mean, y=-log10(padj.PTex_0015), colour = padj_co ) ) +
  geom_point(alpha=0.5, size=1.75) +
#  xlim(c(-6, 6)) + ylim(c(0, 10)) +
  xlab("log2 fold change") + ylab("-log10 adj. p-value")
## PTex 0.15
padj_co <- norm_MassSpecData$padj.PTex_015 <= 0.01
padj_co[which(padj_co)] <- "significant"
padj_co[which(padj_co==FALSE)] <- "non-significant"
fc_ptex_015_volcano <- ggplot(data=cbind(norm_MassSpecData, padj_co), aes(x=FC.PTex_015.mean, y=-log10(padj.PTex_015), colour = padj_co ) ) +
  geom_point(alpha=0.5, size=1.75) +
#  xlim(c(-6, 6)) + ylim(c(0, 10)) +
  xlab("log2 fold change") + ylab("-log10 adj. p-value")
## PTex 1.5
padj_co <- norm_MassSpecData$padj.PTex_15 <= 0.01
padj_co[which(padj_co)] <- "significant"
padj_co[which(padj_co==FALSE)] <- "non-significant"
fc_ptex_15_volcano <- ggplot(data=cbind(norm_MassSpecData, padj_co), aes(x=FC.PTex_15.mean, y=-log10(padj.PTex_15), colour = padj_co ) ) +
  geom_point(alpha=0.5, size=1.75) +
#  xlim(c(-6, 6)) + ylim(c(0, 10)) +
  xlab("log2 fold change") + ylab("-log10 adj. p-value")
```


```
fc_input_0015_volcano
```


```
fc_input_015_volcano
```


```
fc_input_15_volcano
```


```
fc_ptex_0015_volcano
```


```
fc_ptex_015_volcano
```


```
fc_ptex_15_volcano
```


Tidy up:


```
rm(list = ls(pattern = "_scatter"))
rm(list = ls(pattern = "_volcano"))
rm(padj_co)
```

# 5 Matching

To assess performance of PTex to find RBPs, the significantly enriched proteins are compared to other studies. The following studies are taken into account: - Gerstberger review 2014 - Landthaler HEK293 interactome capture - Preiss - SONAR (annotated and predicted) - Hubstenberger P-Body proteins - RICK (high, low and unique) - CARIC


```
## Gerstberger review (2014) RBPs
ge <- read.csv("inputdata/RBP table-Table 1.csv")
## Gerstberger review (2014) TFs
tf <- read.csv("inputdata/human TFs-Table 1.csv")
## Human mRNA interactome capture data:
ic <- read.csv("inputdata/2015-06-09_human_interactomes.csv",na.strings=c("", "NA") )
## Preiss
preiss_gn <- read.csv("inputdata/preiss_genenames.csv", header=T, sep="\t")
## annotated RBPs from SONAR paper (Brannan et al.)
sonar_all <- read.csv("inputdata/Brannan_annotated.txt", header = FALSE)
## Predicted SONAR proteins (with score > 0.79, see Brannan et al. Mol Cell 2016)
sonar_079 <- read.csv("inputdata/Brannan_SONAR_0.79.txt", header = FALSE)
## Hubstenberger P-Body Proteins
hubst <- read.csv("inputdata/hubstenberger.csv", header = TRUE, sep = "\t")
## RICK
rick_high <- read.csv("inputdata/RICK_high_conf.csv", sep = "\t", stringsAsFactors = F)
rick_low <- read.csv("inputdata/RICK_low_conf.csv", sep = "\t", stringsAsFactors = F)
rick_unique <- read.csv("inputdata/RICK_unique.csv", sep="\t", stringsAsFactors = F)
## CARIC
caric <- read.csv("inputdata/caric.csv", sep = "\t", stringsAsFactors = F)
```


## 5.1 Matching table

A matching table is created to perform the matching and store the results. It contains the following identifier columns: - Majority.protein.IDs: the Majority.protein.IDs of the significantly enriched PTex hits - MPIDsNoIso: Majority.protein.IDs with isoforms resolved to canonical form - gene\_name: the gene names of the significantly enriched PTex hits


```
matching_table <- data.frame(Majority.protein.IDs = as.character(sig_norm_MassSpecData$Majority.protein.IDs))
matching_table$Majority.protein.IDs <- apply(X = matching_table, MARGIN = 1, FUN = function(x)unlist(strsplit(as.character(x), split=";")))
matching_table$MPIDsNoIso <- lapply(strsplit(gsub("-[0-9]{1,2}","",sig_norm_MassSpecData$Majority.protein.IDs),split=";"), function(x)unique(x))
matching_table$gene_name <- sig_norm_MassSpecData$Gene.names
```


The canonical MPIDs were matched with all reviewed human UniProt (SwissProt) entries.


```
all_rev_uniprot <- read.table("inputdata/human_proteome_reviewed_291118.list", header=F, col.names = "uniprot")
MPIDsNoIso_rev_stat <- apply(matching_table, 1, function(x) unlist(x$MPIDsNoIso) %in% all_rev_uniprot$uniprot)
MPIDsNoIso.rev <- mapply(x=matching_table$MPIDsNoIso, y=MPIDsNoIso_rev_stat, function(x,y)unlist(x[unlist(y)]))
MPIDsNoIso.rev[unlist(lapply(MPIDsNoIso.rev, function(x)length(x)==0))] <- NA
sum(is.na(MPIDsNoIso.rev))
```


```
[1] 16
```


```
matching_table$MPIDr <- MPIDsNoIso.rev
```


For 16 majority protein ID groups no SwissProt entry could be found.


```
datatable(matching_table[which(MPIDsNoIso.rev%in%"NA"),])
```


Those 16 hits were subjected to manual curation, meaning the accession numbers and gene names were used for a UniProt query. If an unambiguous hit was found it was corrected. The following were corrected:


```
matching_table$MPIDr[74] <- "P43243"
matching_table$MPIDr[83] <- "O75122"
matching_table$MPIDr[93] <- "Q9UBX3"
matching_table$MPIDr[134] <- "O95819"
matching_table$MPIDr[226] <- "Q9UPN3"
matching_table$MPIDr[264] <- "Q96MU7"
matching_table$MPIDr[268] <- "P36776"
matching_table$MPIDr[674] <- "P0DMV8"
matching_table$MPIDr[2937] <- "P0DPB6"
matching_table$MPIDr[3034] <- "P17980"
matching_table$MPIDr[3035] <- "Q9UKV3"
```


After manual correction, the following were still not identifiable.


```
datatable(matching_table[which(matching_table$MPIDr%in%"NA"),])
```


These hits were excluded from further analyses.


```
matching_table <- matching_table[-c(162,169,178,896,3037),]
datatable(matching_table[which(matching_table$MPIDr%in%"NA"),])
```


WARNING:`235 Q13404;A5PLL7 UBE2V1;TMEM189 T;F` stays! Others might, too!

### 5.1.1 Matching with other datasets


```
## Gerstberger RBPs
matching_table$Gerstberger.RBP <- apply(matching_table,1,function(x) any(ge$gene.name %in% unlist(strsplit(as.character(x$gene_name),split=";"))))
## Gerstberger TFs (negative control)
matching_table$Gerstberger.TF <- apply(matching_table,1,function(x) any(tf$gene.name %in% unlist(strsplit(as.character(x$gene_name),split=";"))))
## Landthaler HEK293 interactome capture data
hekmRBP <- subset(ic, ic$HEK293 == "mRNA interactome")
matching_table$Landthaler.mRBP <- apply(matching_table, 1, function(x) any(hekmRBP$Uniprot.entry %in% unlist(x$MPIDr)))
## Preiss
matching_table$Preiss <- apply(matching_table, 1, function(x) any(preiss_gn$gene.name %in% unlist(strsplit(as.character(x$gene_name),split=";"))))
## Annotated RBPs (from the SONAR paper)
matching_table$Brannan.annotated <- apply(matching_table, 1, function(x) any(sonar_all$V1 %in% unlist(strsplit(as.character(x$gene_name),split=";"))))
## SONAR-predicted RBPs
matching_table$Brannan.predicted <- apply(matching_table, 1, function(x) any(sonar_079$V1 %in% unlist(strsplit(as.character(x$gene_name),split=";"))))
## Hubstenberger P-Body proteins
matching_table$Hubstenberger.PBP <- apply(matching_table, 1, function(x) any(hubst$gene.name %in% unlist(strsplit(as.character(x$gene_name),split=";"))))
## RICK data
matching_table$Rick.high <- apply(matching_table, 1, function(x) any(rick_high$Protein_ID %in% unlist(x$MPIDr)))
matching_table$Rick.low <- apply(matching_table, 1, function(x) any(rick_low$Protein_ID %in% unlist(x$MPIDr)))
matching_table$Rick.unique <- apply(matching_table, 1, function(x) any(rick_unique$Protein_ID %in% unlist(x$MPIDr)))
## CARIC RBPs
matching_table$Caric <- apply(matching_table, 1, function(x) any(caric$UniProt.accession %in% unlist(x$MPIDr)))
```


Note that matching via stringsplitted gene names can produce multiple hits in reference for a single PTex hit.

## 5.2 Matching df


```
# inPTex, not inPTex
matching_df <- data.frame(
  match = c("yes", "no"),
  Gerstberger.RBPs = c(round(sum(matching_table$Gerstberger.RBP)/nrow(ge),4), round((nrow(ge) - sum(matching_table$Gerstberger.RBP))/nrow(ge),4)),
  Gerstberger.TFs =c(round(sum(matching_table$Gerstberger.TF)/nrow(tf),4), round((nrow(tf) - sum(matching_table$Gerstberger.TF))/nrow(tf),4)),
  Landthaler = c(round(sum(matching_table$Landthaler.mRBP)/nrow(hekmRBP),4), round((nrow(hekmRBP) - sum(matching_table$Landthaler.mRBP))/nrow(hekmRBP),4)),
  Preiss = c(round(sum(matching_table$Preiss)/nrow(preiss_gn),4), round((nrow(preiss_gn)-sum(matching_table$Preiss))/nrow(preiss_gn),4)),
  Brannan.annotated = c(round(sum(matching_table$Brannan.annotated)/nrow(sonar_all),4), round((nrow(sonar_all) - sum(matching_table$Brannan.annotated))/nrow(sonar_all),4)),
  Brannan.predicted = c(round(sum(matching_table$Brannan.predicted)/nrow(sonar_079),4), round((nrow(sonar_079) - sum(matching_table$Brannan.predicted))/nrow(sonar_079),4)),
  Hubstenberger.PBP = c(round(sum(matching_table$Hubstenberger.PBP)/nrow(hubst),4), round((nrow(hubst) - sum(matching_table$Hubstenberger.PBP))/nrow(hubst),4)),
  Rick.high = c(round(sum(matching_table$Rick.high)/nrow(rick_high),4), round((nrow(rick_high) - sum(matching_table$Rick.high))/nrow(rick_high),4)),
  Rick.low = c(round(sum(matching_table$Rick.low)/nrow(rick_low),4), round((nrow(rick_low) - sum(matching_table$Rick.low))/nrow(rick_low),4)),
  Rick.unique = c(round(sum(matching_table$Rick.unique)/nrow(rick_unique),4), round((nrow(rick_unique) - sum(matching_table$Rick.unique))/nrow(rick_unique),4)),
  Caric = c(round(sum(matching_table$Caric)/nrow(caric),4), round((nrow(caric) - sum(matching_table$Caric))/nrow(caric),4))
)
```


```
ggplot(melt(matching_df, id.vars = "match"), aes(x=variable, y=value, fill=match)) +
  geom_bar(stat = "identity", alpha=.6) +
  ylab("fraction") + xlab("") +
  theme(axis.text.x = element_text(angle = 45, hjust = 1))
```

# 6 Annotations

The significantly enriched hits are annotated with - isoelectric point - molecular weight - hydrophobicity - (RBD) - (GO)

For that a list of UniProt accession numbers is needed. For that the `MPIDr` of the matching table is used, while for entries with multiple accession numbers, only the first is used.


```
accessions <- unlist(lapply(X=matching_table$MPIDr, FUN = function(x) strsplit(as.character(x), split = ";")[[1]][1]))
write.table(accessions, "accessions.tsv", row.names = F, col.names = F)
```


## 6.1 Isoelectric Point, Molecular Weight, Sequences and hydrophobicity

For comparison of the MS data with the human proteome a dataset from `isoelectricpointdb.org` was retrieved (including isoelectric points, molecular weights and sequences) (as of 29.11.18):


```
isoelectricpointdb <- read.csv("inputdata/UP000005640_9606_all.fasta.pI.csv", header=TRUE, sep=",")
```


```
IPDB_matching <- data.frame(
  UniProt = accessions,
  Sequence = rep(NA,length(accessions)),
  MolWeight = rep(NA,length(accessions)),
  IPC_protein = rep(NA,length(accessions)),
  Hydrophobicity = rep(NA,length(accessions))
)
```


```
for(i in seq(nrow(IPDB_matching))){
  try(IPDB_matching$Sequence[i] <- as.character(isoelectricpointdb$sequence[grep(pattern = paste("|",IPDB_matching$UniProt[i],"|", sep=""), x = isoelectricpointdb$header, fixed = TRUE)]))
  try(IPDB_matching$MolWeight[i] <- isoelectricpointdb$molecular_weight[grep(pattern = paste("|",IPDB_matching$UniProt[i],"|", sep=""), x = isoelectricpointdb$header, fixed = TRUE)])
  try(IPDB_matching$IPC_protein[i] <- isoelectricpointdb$IPC_protein[grep(pattern = paste("|",IPDB_matching$UniProt[i],"|", sep=""), x = isoelectricpointdb$header, fixed = TRUE)])
}
```


```
Error in IPDB_matching$Sequence[i] <- as.character(isoelectricpointdb$sequence[grep(pattern = paste("|",  : 
  replacement has length zero
Error in IPDB_matching$MolWeight[i] <- isoelectricpointdb$molecular_weight[grep(pattern = paste("|",  : 
  replacement has length zero
Error in IPDB_matching$IPC_protein[i] <- isoelectricpointdb$IPC_protein[grep(pattern = paste("|",  : 
  replacement has length zero
```


Not found: P0DPB6 which is >sp|Q9Y2S0|RPAC2\_HUMAN DNA-directed RNA polymerases I and III subunit RPAC2 OS=Homo sapiens GN=POLR1D PE=1 SV=1


```
for(i in seq(nrow(IPDB_matching))){
  try(IPDB_matching$Hydrophobicity[i] <- aaComp(IPDB_matching$Sequence[i])[[1]]["NonPolar","Mole%"])
}
```


```
Sequence 1 has unrecognized amino acid types. Output value might be wrong calculatedSequence 1 has unrecognized amino acid types. Output value might be wrong calculatedSequence 1 has unrecognized amino acid types. Output value might be wrong calculated
```


`all_rev_uniprot` were matched with the isoelectricpointdb. (Note that the following chunk runs very long, because of the low speed of for loops)


```
IPDB_matching_proteome <- data.frame(
  UniProt = as.character(all_rev_uniprot$uniprot),
  Sequence = rep(NA,length(all_rev_uniprot)),
  MolWeight = rep(NA,length(all_rev_uniprot)),
  IPC_protein = rep(NA,length(all_rev_uniprot)),
  Hydrophobicity = rep(NA,length(all_rev_uniprot))
)
for(i in seq(nrow(IPDB_matching_proteome))){
  try(IPDB_matching_proteome$Sequence[i] <- as.character(isoelectricpointdb$sequence[grep(pattern = paste("|",IPDB_matching_proteome$UniProt[i],"|", sep=""), x = isoelectricpointdb$header, fixed = TRUE)]))
  try(IPDB_matching_proteome$MolWeight[i] <- isoelectricpointdb$molecular_weight[grep(pattern = paste("|",IPDB_matching_proteome$UniProt[i],"|", sep=""), x = isoelectricpointdb$header, fixed = TRUE)])
  try(IPDB_matching_proteome$IPC_protein[i] <- isoelectricpointdb$IPC_protein[grep(pattern = paste("|",IPDB_matching_proteome$UniProt[i],"|", sep=""), x = isoelectricpointdb$header, fixed = TRUE)])
}
```


```
Error in IPDB_matching_proteome$Sequence[i] <- as.character(isoelectricpointdb$sequence[grep(pattern = paste("|",  : 
  replacement has length zero
Error in IPDB_matching_proteome$MolWeight[i] <- isoelectricpointdb$molecular_weight[grep(pattern = paste("|",  : 
  replacement has length zero
Error in IPDB_matching_proteome$IPC_protein[i] <- isoelectricpointdb$IPC_protein[grep(pattern = paste("|",  : 
  replacement has length zero
Error in IPDB_matching_proteome$Sequence[i] <- as.character(isoelectricpointdb$sequence[grep(pattern = paste("|",  : 
  replacement has length zero
Error in IPDB_matching_proteome$MolWeight[i] <- isoelectricpointdb$molecular_weight[grep(pattern = paste("|",  : 
  replacement has length zero
Error in IPDB_matching_proteome$IPC_protein[i] <- isoelectricpointdb$IPC_protein[grep(pattern = paste("|",  : 
  replacement has length zero
Error in IPDB_matching_proteome$Sequence[i] <- as.character(isoelectricpointdb$sequence[grep(pattern = paste("|",  : 
  replacement has length zero
Error in IPDB_matching_proteome$MolWeight[i] <- isoelectricpointdb$molecular_weight[grep(pattern = paste("|",  : 
  replacement has length zero
Error in IPDB_matching_proteome$IPC_protein[i] <- isoelectricpointdb$IPC_protein[grep(pattern = paste("|",  : 
  replacement has length zero
Error in IPDB_matching_proteome$Sequence[i] <- as.character(isoelectricpointdb$sequence[grep(pattern = paste("|",  : 
  replacement has length zero
Error in IPDB_matching_proteome$MolWeight[i] <- isoelectricpointdb$molecular_weight[grep(pattern = paste("|",  : 
  replacement has length zero
Error in IPDB_matching_proteome$IPC_protein[i] <- isoelectricpointdb$IPC_protein[grep(pattern = paste("|",  : 
  replacement has length zero
Error in IPDB_matching_proteome$Sequence[i] <- as.character(isoelectricpointdb$sequence[grep(pattern = paste("|",  : 
  replacement has length zero
Error in IPDB_matching_proteome$MolWeight[i] <- isoelectricpointdb$molecular_weight[grep(pattern = paste("|",  : 
  replacement has length zero
Error in IPDB_matching_proteome$IPC_protein[i] <- isoelectricpointdb$IPC_protein[grep(pattern = paste("|",  : 
  replacement has length zero
Error in IPDB_matching_proteome$Sequence[i] <- as.character(isoelectricpointdb$sequence[grep(pattern = paste("|",  : 
  replacement has length zero
Error in IPDB_matching_proteome$MolWeight[i] <- isoelectricpointdb$molecular_weight[grep(pattern = paste("|",  : 
  replacement has length zero
Error in IPDB_matching_proteome$IPC_protein[i] <- isoelectricpointdb$IPC_protein[grep(pattern = paste("|",  : 
  replacement has length zero
Error in IPDB_matching_proteome$Sequence[i] <- as.character(isoelectricpointdb$sequence[grep(pattern = paste("|",  : 
  replacement has length zero
Error in IPDB_matching_proteome$MolWeight[i] <- isoelectricpointdb$molecular_weight[grep(pattern = paste("|",  : 
  replacement has length zero
Error in IPDB_matching_proteome$IPC_protein[i] <- isoelectricpointdb$IPC_protein[grep(pattern = paste("|",  : 
  replacement has length zero
Error in IPDB_matching_proteome$Sequence[i] <- as.character(isoelectricpointdb$sequence[grep(pattern = paste("|",  : 
  replacement has length zero
Error in IPDB_matching_proteome$MolWeight[i] <- isoelectricpointdb$molecular_weight[grep(pattern = paste("|",  : 
  replacement has length zero
Error in IPDB_matching_proteome$IPC_protein[i] <- isoelectricpointdb$IPC_protein[grep(pattern = paste("|",  : 
  replacement has length zero
Error in IPDB_matching_proteome$Sequence[i] <- as.character(isoelectricpointdb$sequence[grep(pattern = paste("|",  : 
  replacement has length zero
Error in IPDB_matching_proteome$MolWeight[i] <- isoelectricpointdb$molecular_weight[grep(pattern = paste("|",  : 
  replacement has length zero
Error in IPDB_matching_proteome$IPC_protein[i] <- isoelectricpointdb$IPC_protein[grep(pattern = paste("|",  : 
  replacement has length zero
Error in IPDB_matching_proteome$Sequence[i] <- as.character(isoelectricpointdb$sequence[grep(pattern = paste("|",  : 
  replacement has length zero
Error in IPDB_matching_proteome$MolWeight[i] <- isoelectricpointdb$molecular_weight[grep(pattern = paste("|",  : 
  replacement has length zero
Error in IPDB_matching_proteome$IPC_protein[i] <- isoelectricpointdb$IPC_protein[grep(pattern = paste("|",  : 
  replacement has length zero
Error in IPDB_matching_proteome$Sequence[i] <- as.character(isoelectricpointdb$sequence[grep(pattern = paste("|",  : 
  replacement has length zero
Error in IPDB_matching_proteome$MolWeight[i] <- isoelectricpointdb$molecular_weight[grep(pattern = paste("|",  : 
  replacement has length zero
Error in IPDB_matching_proteome$IPC_protein[i] <- isoelectricpointdb$IPC_protein[grep(pattern = paste("|",  : 
  replacement has length zero
Error in IPDB_matching_proteome$Sequence[i] <- as.character(isoelectricpointdb$sequence[grep(pattern = paste("|",  : 
  replacement has length zero
Error in IPDB_matching_proteome$MolWeight[i] <- isoelectricpointdb$molecular_weight[grep(pattern = paste("|",  : 
  replacement has length zero
Error in IPDB_matching_proteome$IPC_protein[i] <- isoelectricpointdb$IPC_protein[grep(pattern = paste("|",  : 
  replacement has length zero
Error in IPDB_matching_proteome$Sequence[i] <- as.character(isoelectricpointdb$sequence[grep(pattern = paste("|",  : 
  replacement has length zero
Error in IPDB_matching_proteome$MolWeight[i] <- isoelectricpointdb$molecular_weight[grep(pattern = paste("|",  : 
  replacement has length zero
Error in IPDB_matching_proteome$IPC_protein[i] <- isoelectricpointdb$IPC_protein[grep(pattern = paste("|",  : 
  replacement has length zero
Error in IPDB_matching_proteome$Sequence[i] <- as.character(isoelectricpointdb$sequence[grep(pattern = paste("|",  : 
  replacement has length zero
Error in IPDB_matching_proteome$MolWeight[i] <- isoelectricpointdb$molecular_weight[grep(pattern = paste("|",  : 
  replacement has length zero
Error in IPDB_matching_proteome$IPC_protein[i] <- isoelectricpointdb$IPC_protein[grep(pattern = paste("|",  : 
  replacement has length zero
Error in IPDB_matching_proteome$Sequence[i] <- as.character(isoelectricpointdb$sequence[grep(pattern = paste("|",  : 
  replacement has length zero
Error in IPDB_matching_proteome$MolWeight[i] <- isoelectricpointdb$molecular_weight[grep(pattern = paste("|",  : 
  replacement has length zero
Error in IPDB_matching_proteome$IPC_protein[i] <- isoelectricpointdb$IPC_protein[grep(pattern = paste("|",  : 
  replacement has length zero
Error in IPDB_matching_proteome$Sequence[i] <- as.character(isoelectricpointdb$sequence[grep(pattern = paste("|",  : 
  replacement has length zero
Error in IPDB_matching_proteome$MolWeight[i] <- isoelectricpointdb$molecular_weight[grep(pattern = paste("|",  : 
  replacement has length zero
Error in IPDB_matching_proteome$IPC_protein[i] <- isoelectricpointdb$IPC_protein[grep(pattern = paste("|",  : 
  replacement has length zero
Error in IPDB_matching_proteome$Sequence[i] <- as.character(isoelectricpointdb$sequence[grep(pattern = paste("|",  : 
  replacement has length zero
Error in IPDB_matching_proteome$MolWeight[i] <- isoelectricpointdb$molecular_weight[grep(pattern = paste("|",  : 
  replacement has length zero
Error in IPDB_matching_proteome$IPC_protein[i] <- isoelectricpointdb$IPC_protein[grep(pattern = paste("|",  : 
  replacement has length zero
Error in IPDB_matching_proteome$Sequence[i] <- as.character(isoelectricpointdb$sequence[grep(pattern = paste("|",  : 
  replacement has length zero
Error in IPDB_matching_proteome$MolWeight[i] <- isoelectricpointdb$molecular_weight[grep(pattern = paste("|",  : 
  replacement has length zero
Error in IPDB_matching_proteome$IPC_protein[i] <- isoelectricpointdb$IPC_protein[grep(pattern = paste("|",  : 
  replacement has length zero
Error in IPDB_matching_proteome$Sequence[i] <- as.character(isoelectricpointdb$sequence[grep(pattern = paste("|",  : 
  replacement has length zero
Error in IPDB_matching_proteome$MolWeight[i] <- isoelectricpointdb$molecular_weight[grep(pattern = paste("|",  : 
  replacement has length zero
Error in IPDB_matching_proteome$IPC_protein[i] <- isoelectricpointdb$IPC_protein[grep(pattern = paste("|",  : 
  replacement has length zero
Error in IPDB_matching_proteome$Sequence[i] <- as.character(isoelectricpointdb$sequence[grep(pattern = paste("|",  : 
  replacement has length zero
Error in IPDB_matching_proteome$MolWeight[i] <- isoelectricpointdb$molecular_weight[grep(pattern = paste("|",  : 
  replacement has length zero
Error in IPDB_matching_proteome$IPC_protein[i] <- isoelectricpointdb$IPC_protein[grep(pattern = paste("|",  : 
  replacement has length zero
Error in IPDB_matching_proteome$Sequence[i] <- as.character(isoelectricpointdb$sequence[grep(pattern = paste("|",  : 
  replacement has length zero
Error in IPDB_matching_proteome$MolWeight[i] <- isoelectricpointdb$molecular_weight[grep(pattern = paste("|",  : 
  replacement has length zero
Error in IPDB_matching_proteome$IPC_protein[i] <- isoelectricpointdb$IPC_protein[grep(pattern = paste("|",  : 
  replacement has length zero
Error in IPDB_matching_proteome$Sequence[i] <- as.character(isoelectricpointdb$sequence[grep(pattern = paste("|",  : 
  replacement has length zero
Error in IPDB_matching_proteome$MolWeight[i] <- isoelectricpointdb$molecular_weight[grep(pattern = paste("|",  : 
  replacement has length zero
Error in IPDB_matching_proteome$IPC_protein[i] <- isoelectricpointdb$IPC_protein[grep(pattern = paste("|",  : 
  replacement has length zero
Error in IPDB_matching_proteome$Sequence[i] <- as.character(isoelectricpointdb$sequence[grep(pattern = paste("|",  : 
  replacement has length zero
Error in IPDB_matching_proteome$MolWeight[i] <- isoelectricpointdb$molecular_weight[grep(pattern = paste("|",  : 
  replacement has length zero
Error in IPDB_matching_proteome$IPC_protein[i] <- isoelectricpointdb$IPC_protein[grep(pattern = paste("|",  : 
  replacement has length zero
Error in IPDB_matching_proteome$Sequence[i] <- as.character(isoelectricpointdb$sequence[grep(pattern = paste("|",  : 
  replacement has length zero
Error in IPDB_matching_proteome$MolWeight[i] <- isoelectricpointdb$molecular_weight[grep(pattern = paste("|",  : 
  replacement has length zero
Error in IPDB_matching_proteome$IPC_protein[i] <- isoelectricpointdb$IPC_protein[grep(pattern = paste("|",  : 
  replacement has length zero
Error in IPDB_matching_proteome$Sequence[i] <- as.character(isoelectricpointdb$sequence[grep(pattern = paste("|",  : 
  replacement has length zero
Error in IPDB_matching_proteome$MolWeight[i] <- isoelectricpointdb$molecular_weight[grep(pattern = paste("|",  : 
  replacement has length zero
Error in IPDB_matching_proteome$IPC_protein[i] <- isoelectricpointdb$IPC_protein[grep(pattern = paste("|",  : 
  replacement has length zero
Error in IPDB_matching_proteome$Sequence[i] <- as.character(isoelectricpointdb$sequence[grep(pattern = paste("|",  : 
  replacement has length zero
Error in IPDB_matching_proteome$MolWeight[i] <- isoelectricpointdb$molecular_weight[grep(pattern = paste("|",  : 
  replacement has length zero
Error in IPDB_matching_proteome$IPC_protein[i] <- isoelectricpointdb$IPC_protein[grep(pattern = paste("|",  : 
  replacement has length zero
Error in IPDB_matching_proteome$Sequence[i] <- as.character(isoelectricpointdb$sequence[grep(pattern = paste("|",  : 
  replacement has length zero
Error in IPDB_matching_proteome$MolWeight[i] <- isoelectricpointdb$molecular_weight[grep(pattern = paste("|",  : 
  replacement has length zero
Error in IPDB_matching_proteome$IPC_protein[i] <- isoelectricpointdb$IPC_protein[grep(pattern = paste("|",  : 
  replacement has length zero
Error in IPDB_matching_proteome$Sequence[i] <- as.character(isoelectricpointdb$sequence[grep(pattern = paste("|",  : 
  replacement has length zero
Error in IPDB_matching_proteome$MolWeight[i] <- isoelectricpointdb$molecular_weight[grep(pattern = paste("|",  : 
  replacement has length zero
Error in IPDB_matching_proteome$IPC_protein[i] <- isoelectricpointdb$IPC_protein[grep(pattern = paste("|",  : 
  replacement has length zero
Error in IPDB_matching_proteome$Sequence[i] <- as.character(isoelectricpointdb$sequence[grep(pattern = paste("|",  : 
  replacement has length zero
Error in IPDB_matching_proteome$MolWeight[i] <- isoelectricpointdb$molecular_weight[grep(pattern = paste("|",  : 
  replacement has length zero
Error in IPDB_matching_proteome$IPC_protein[i] <- isoelectricpointdb$IPC_protein[grep(pattern = paste("|",  : 
  replacement has length zero
Error in IPDB_matching_proteome$Sequence[i] <- as.character(isoelectricpointdb$sequence[grep(pattern = paste("|",  : 
  replacement has length zero
Error in IPDB_matching_proteome$MolWeight[i] <- isoelectricpointdb$molecular_weight[grep(pattern = paste("|",  : 
  replacement has length zero
Error in IPDB_matching_proteome$IPC_protein[i] <- isoelectricpointdb$IPC_protein[grep(pattern = paste("|",  : 
  replacement has length zero
Error in IPDB_matching_proteome$Sequence[i] <- as.character(isoelectricpointdb$sequence[grep(pattern = paste("|",  : 
  replacement has length zero
Error in IPDB_matching_proteome$MolWeight[i] <- isoelectricpointdb$molecular_weight[grep(pattern = paste("|",  : 
  replacement has length zero
Error in IPDB_matching_proteome$IPC_protein[i] <- isoelectricpointdb$IPC_protein[grep(pattern = paste("|",  : 
  replacement has length zero
Error in IPDB_matching_proteome$Sequence[i] <- as.character(isoelectricpointdb$sequence[grep(pattern = paste("|",  : 
  replacement has length zero
Error in IPDB_matching_proteome$MolWeight[i] <- isoelectricpointdb$molecular_weight[grep(pattern = paste("|",  : 
  replacement has length zero
Error in IPDB_matching_proteome$IPC_protein[i] <- isoelectricpointdb$IPC_protein[grep(pattern = paste("|",  : 
  replacement has length zero
Error in IPDB_matching_proteome$Sequence[i] <- as.character(isoelectricpointdb$sequence[grep(pattern = paste("|",  : 
  replacement has length zero
Error in IPDB_matching_proteome$MolWeight[i] <- isoelectricpointdb$molecular_weight[grep(pattern = paste("|",  : 
  replacement has length zero
Error in IPDB_matching_proteome$IPC_protein[i] <- isoelectricpointdb$IPC_protein[grep(pattern = paste("|",  : 
  replacement has length zero
Error in IPDB_matching_proteome$Sequence[i] <- as.character(isoelectricpointdb$sequence[grep(pattern = paste("|",  : 
  replacement has length zero
Error in IPDB_matching_proteome$MolWeight[i] <- isoelectricpointdb$molecular_weight[grep(pattern = paste("|",  : 
  replacement has length zero
Error in IPDB_matching_proteome$IPC_protein[i] <- isoelectricpointdb$IPC_protein[grep(pattern = paste("|",  : 
  replacement has length zero
Error in IPDB_matching_proteome$Sequence[i] <- as.character(isoelectricpointdb$sequence[grep(pattern = paste("|",  : 
  replacement has length zero
Error in IPDB_matching_proteome$MolWeight[i] <- isoelectricpointdb$molecular_weight[grep(pattern = paste("|",  : 
  replacement has length zero
Error in IPDB_matching_proteome$IPC_protein[i] <- isoelectricpointdb$IPC_protein[grep(pattern = paste("|",  : 
  replacement has length zero
Error in IPDB_matching_proteome$Sequence[i] <- as.character(isoelectricpointdb$sequence[grep(pattern = paste("|",  : 
  replacement has length zero
Error in IPDB_matching_proteome$MolWeight[i] <- isoelectricpointdb$molecular_weight[grep(pattern = paste("|",  : 
  replacement has length zero
Error in IPDB_matching_proteome$IPC_protein[i] <- isoelectricpointdb$IPC_protein[grep(pattern = paste("|",  : 
  replacement has length zero
Error in IPDB_matching_proteome$Sequence[i] <- as.character(isoelectricpointdb$sequence[grep(pattern = paste("|",  : 
  replacement has length zero
Error in IPDB_matching_proteome$MolWeight[i] <- isoelectricpointdb$molecular_weight[grep(pattern = paste("|",  : 
  replacement has length zero
Error in IPDB_matching_proteome$IPC_protein[i] <- isoelectricpointdb$IPC_protein[grep(pattern = paste("|",  : 
  replacement has length zero
Error in IPDB_matching_proteome$Sequence[i] <- as.character(isoelectricpointdb$sequence[grep(pattern = paste("|",  : 
  replacement has length zero
Error in IPDB_matching_proteome$MolWeight[i] <- isoelectricpointdb$molecular_weight[grep(pattern = paste("|",  : 
  replacement has length zero
Error in IPDB_matching_proteome$IPC_protein[i] <- isoelectricpointdb$IPC_protein[grep(pattern = paste("|",  : 
  replacement has length zero
Error in IPDB_matching_proteome$Sequence[i] <- as.character(isoelectricpointdb$sequence[grep(pattern = paste("|",  : 
  replacement has length zero
Error in IPDB_matching_proteome$MolWeight[i] <- isoelectricpointdb$molecular_weight[grep(pattern = paste("|",  : 
  replacement has length zero
Error in IPDB_matching_proteome$IPC_protein[i] <- isoelectricpointdb$IPC_protein[grep(pattern = paste("|",  : 
  replacement has length zero
Error in IPDB_matching_proteome$Sequence[i] <- as.character(isoelectricpointdb$sequence[grep(pattern = paste("|",  : 
  replacement has length zero
Error in IPDB_matching_proteome$MolWeight[i] <- isoelectricpointdb$molecular_weight[grep(pattern = paste("|",  : 
  replacement has length zero
Error in IPDB_matching_proteome$IPC_protein[i] <- isoelectricpointdb$IPC_protein[grep(pattern = paste("|",  : 
  replacement has length zero
Error in IPDB_matching_proteome$Sequence[i] <- as.character(isoelectricpointdb$sequence[grep(pattern = paste("|",  : 
  replacement has length zero
Error in IPDB_matching_proteome$MolWeight[i] <- isoelectricpointdb$molecular_weight[grep(pattern = paste("|",  : 
  replacement has length zero
Error in IPDB_matching_proteome$IPC_protein[i] <- isoelectricpointdb$IPC_protein[grep(pattern = paste("|",  : 
  replacement has length zero
Error in IPDB_matching_proteome$Sequence[i] <- as.character(isoelectricpointdb$sequence[grep(pattern = paste("|",  : 
  replacement has length zero
Error in IPDB_matching_proteome$MolWeight[i] <- isoelectricpointdb$molecular_weight[grep(pattern = paste("|",  : 
  replacement has length zero
Error in IPDB_matching_proteome$IPC_protein[i] <- isoelectricpointdb$IPC_protein[grep(pattern = paste("|",  : 
  replacement has length zero
Error in IPDB_matching_proteome$Sequence[i] <- as.character(isoelectricpointdb$sequence[grep(pattern = paste("|",  : 
  replacement has length zero
Error in IPDB_matching_proteome$MolWeight[i] <- isoelectricpointdb$molecular_weight[grep(pattern = paste("|",  : 
  replacement has length zero
Error in IPDB_matching_proteome$IPC_protein[i] <- isoelectricpointdb$IPC_protein[grep(pattern = paste("|",  : 
  replacement has length zero
Error in IPDB_matching_proteome$Sequence[i] <- as.character(isoelectricpointdb$sequence[grep(pattern = paste("|",  : 
  replacement has length zero
Error in IPDB_matching_proteome$MolWeight[i] <- isoelectricpointdb$molecular_weight[grep(pattern = paste("|",  : 
  replacement has length zero
Error in IPDB_matching_proteome$IPC_protein[i] <- isoelectricpointdb$IPC_protein[grep(pattern = paste("|",  : 
  replacement has length zero
Error in IPDB_matching_proteome$Sequence[i] <- as.character(isoelectricpointdb$sequence[grep(pattern = paste("|",  : 
  replacement has length zero
Error in IPDB_matching_proteome$MolWeight[i] <- isoelectricpointdb$molecular_weight[grep(pattern = paste("|",  : 
  replacement has length zero
Error in IPDB_matching_proteome$IPC_protein[i] <- isoelectricpointdb$IPC_protein[grep(pattern = paste("|",  : 
  replacement has length zero
Error in IPDB_matching_proteome$Sequence[i] <- as.character(isoelectricpointdb$sequence[grep(pattern = paste("|",  : 
  replacement has length zero
Error in IPDB_matching_proteome$MolWeight[i] <- isoelectricpointdb$molecular_weight[grep(pattern = paste("|",  : 
  replacement has length zero
Error in IPDB_matching_proteome$IPC_protein[i] <- isoelectricpointdb$IPC_protein[grep(pattern = paste("|",  : 
  replacement has length zero
Error in IPDB_matching_proteome$Sequence[i] <- as.character(isoelectricpointdb$sequence[grep(pattern = paste("|",  : 
  replacement has length zero
Error in IPDB_matching_proteome$MolWeight[i] <- isoelectricpointdb$molecular_weight[grep(pattern = paste("|",  : 
  replacement has length zero
Error in IPDB_matching_proteome$IPC_protein[i] <- isoelectricpointdb$IPC_protein[grep(pattern = paste("|",  : 
  replacement has length zero
Error in IPDB_matching_proteome$Sequence[i] <- as.character(isoelectricpointdb$sequence[grep(pattern = paste("|",  : 
  replacement has length zero
Error in IPDB_matching_proteome$MolWeight[i] <- isoelectricpointdb$molecular_weight[grep(pattern = paste("|",  : 
  replacement has length zero
Error in IPDB_matching_proteome$IPC_protein[i] <- isoelectricpointdb$IPC_protein[grep(pattern = paste("|",  : 
  replacement has length zero
Error in IPDB_matching_proteome$Sequence[i] <- as.character(isoelectricpointdb$sequence[grep(pattern = paste("|",  : 
  replacement has length zero
Error in IPDB_matching_proteome$MolWeight[i] <- isoelectricpointdb$molecular_weight[grep(pattern = paste("|",  : 
  replacement has length zero
Error in IPDB_matching_proteome$IPC_protein[i] <- isoelectricpointdb$IPC_protein[grep(pattern = paste("|",  : 
  replacement has length zero
Error in IPDB_matching_proteome$Sequence[i] <- as.character(isoelectricpointdb$sequence[grep(pattern = paste("|",  : 
  replacement has length zero
Error in IPDB_matching_proteome$MolWeight[i] <- isoelectricpointdb$molecular_weight[grep(pattern = paste("|",  : 
  replacement has length zero
Error in IPDB_matching_proteome$IPC_protein[i] <- isoelectricpointdb$IPC_protein[grep(pattern = paste("|",  : 
  replacement has length zero
Error in IPDB_matching_proteome$Sequence[i] <- as.character(isoelectricpointdb$sequence[grep(pattern = paste("|",  : 
  replacement has length zero
Error in IPDB_matching_proteome$MolWeight[i] <- isoelectricpointdb$molecular_weight[grep(pattern = paste("|",  : 
  replacement has length zero
Error in IPDB_matching_proteome$IPC_protein[i] <- isoelectricpointdb$IPC_protein[grep(pattern = paste("|",  : 
  replacement has length zero
Error in IPDB_matching_proteome$Sequence[i] <- as.character(isoelectricpointdb$sequence[grep(pattern = paste("|",  : 
  replacement has length zero
Error in IPDB_matching_proteome$MolWeight[i] <- isoelectricpointdb$molecular_weight[grep(pattern = paste("|",  : 
  replacement has length zero
Error in IPDB_matching_proteome$IPC_protein[i] <- isoelectricpointdb$IPC_protein[grep(pattern = paste("|",  : 
  replacement has length zero
Error in IPDB_matching_proteome$Sequence[i] <- as.character(isoelectricpointdb$sequence[grep(pattern = paste("|",  : 
  replacement has length zero
Error in IPDB_matching_proteome$MolWeight[i] <- isoelectricpointdb$molecular_weight[grep(pattern = paste("|",  : 
  replacement has length zero
Error in IPDB_matching_proteome$IPC_protein[i] <- isoelectricpointdb$IPC_protein[grep(pattern = paste("|",  : 
  replacement has length zero
Error in IPDB_matching_proteome$Sequence[i] <- as.character(isoelectricpointdb$sequence[grep(pattern = paste("|",  : 
  replacement has length zero
Error in IPDB_matching_proteome$MolWeight[i] <- isoelectricpointdb$molecular_weight[grep(pattern = paste("|",  : 
  replacement has length zero
Error in IPDB_matching_proteome$IPC_protein[i] <- isoelectricpointdb$IPC_protein[grep(pattern = paste("|",  : 
  replacement has length zero
Error in IPDB_matching_proteome$Sequence[i] <- as.character(isoelectricpointdb$sequence[grep(pattern = paste("|",  : 
  replacement has length zero
Error in IPDB_matching_proteome$MolWeight[i] <- isoelectricpointdb$molecular_weight[grep(pattern = paste("|",  : 
  replacement has length zero
Error in IPDB_matching_proteome$IPC_protein[i] <- isoelectricpointdb$IPC_protein[grep(pattern = paste("|",  : 
  replacement has length zero
Error in IPDB_matching_proteome$Sequence[i] <- as.character(isoelectricpointdb$sequence[grep(pattern = paste("|",  : 
  replacement has length zero
Error in IPDB_matching_proteome$MolWeight[i] <- isoelectricpointdb$molecular_weight[grep(pattern = paste("|",  : 
  replacement has length zero
Error in IPDB_matching_proteome$IPC_protein[i] <- isoelectricpointdb$IPC_protein[grep(pattern = paste("|",  : 
  replacement has length zero
Error in IPDB_matching_proteome$Sequence[i] <- as.character(isoelectricpointdb$sequence[grep(pattern = paste("|",  : 
  replacement has length zero
Error in IPDB_matching_proteome$MolWeight[i] <- isoelectricpointdb$molecular_weight[grep(pattern = paste("|",  : 
  replacement has length zero
Error in IPDB_matching_proteome$IPC_protein[i] <- isoelectricpointdb$IPC_protein[grep(pattern = paste("|",  : 
  replacement has length zero
Error in IPDB_matching_proteome$Sequence[i] <- as.character(isoelectricpointdb$sequence[grep(pattern = paste("|",  : 
  replacement has length zero
Error in IPDB_matching_proteome$MolWeight[i] <- isoelectricpointdb$molecular_weight[grep(pattern = paste("|",  : 
  replacement has length zero
Error in IPDB_matching_proteome$IPC_protein[i] <- isoelectricpointdb$IPC_protein[grep(pattern = paste("|",  : 
  replacement has length zero
Error in IPDB_matching_proteome$Sequence[i] <- as.character(isoelectricpointdb$sequence[grep(pattern = paste("|",  : 
  replacement has length zero
Error in IPDB_matching_proteome$MolWeight[i] <- isoelectricpointdb$molecular_weight[grep(pattern = paste("|",  : 
  replacement has length zero
Error in IPDB_matching_proteome$IPC_protein[i] <- isoelectricpointdb$IPC_protein[grep(pattern = paste("|",  : 
  replacement has length zero
Error in IPDB_matching_proteome$Sequence[i] <- as.character(isoelectricpointdb$sequence[grep(pattern = paste("|",  : 
  replacement has length zero
Error in IPDB_matching_proteome$MolWeight[i] <- isoelectricpointdb$molecular_weight[grep(pattern = paste("|",  : 
  replacement has length zero
Error in IPDB_matching_proteome$IPC_protein[i] <- isoelectricpointdb$IPC_protein[grep(pattern = paste("|",  : 
  replacement has length zero
Error in IPDB_matching_proteome$Sequence[i] <- as.character(isoelectricpointdb$sequence[grep(pattern = paste("|",  : 
  replacement has length zero
Error in IPDB_matching_proteome$MolWeight[i] <- isoelectricpointdb$molecular_weight[grep(pattern = paste("|",  : 
  replacement has length zero
Error in IPDB_matching_proteome$IPC_protein[i] <- isoelectricpointdb$IPC_protein[grep(pattern = paste("|",  : 
  replacement has length zero
Error in IPDB_matching_proteome$Sequence[i] <- as.character(isoelectricpointdb$sequence[grep(pattern = paste("|",  : 
  replacement has length zero
Error in IPDB_matching_proteome$MolWeight[i] <- isoelectricpointdb$molecular_weight[grep(pattern = paste("|",  : 
  replacement has length zero
Error in IPDB_matching_proteome$IPC_protein[i] <- isoelectricpointdb$IPC_protein[grep(pattern = paste("|",  : 
  replacement has length zero
Error in IPDB_matching_proteome$Sequence[i] <- as.character(isoelectricpointdb$sequence[grep(pattern = paste("|",  : 
  replacement has length zero
Error in IPDB_matching_proteome$MolWeight[i] <- isoelectricpointdb$molecular_weight[grep(pattern = paste("|",  : 
  replacement has length zero
Error in IPDB_matching_proteome$IPC_protein[i] <- isoelectricpointdb$IPC_protein[grep(pattern = paste("|",  : 
  replacement has length zero
Error in IPDB_matching_proteome$Sequence[i] <- as.character(isoelectricpointdb$sequence[grep(pattern = paste("|",  : 
  replacement has length zero
Error in IPDB_matching_proteome$MolWeight[i] <- isoelectricpointdb$molecular_weight[grep(pattern = paste("|",  : 
  replacement has length zero
Error in IPDB_matching_proteome$IPC_protein[i] <- isoelectricpointdb$IPC_protein[grep(pattern = paste("|",  : 
  replacement has length zero
Error in IPDB_matching_proteome$Sequence[i] <- as.character(isoelectricpointdb$sequence[grep(pattern = paste("|",  : 
  replacement has length zero
Error in IPDB_matching_proteome$MolWeight[i] <- isoelectricpointdb$molecular_weight[grep(pattern = paste("|",  : 
  replacement has length zero
Error in IPDB_matching_proteome$IPC_protein[i] <- isoelectricpointdb$IPC_protein[grep(pattern = paste("|",  : 
  replacement has length zero
Error in IPDB_matching_proteome$Sequence[i] <- as.character(isoelectricpointdb$sequence[grep(pattern = paste("|",  : 
  replacement has length zero
Error in IPDB_matching_proteome$MolWeight[i] <- isoelectricpointdb$molecular_weight[grep(pattern = paste("|",  : 
  replacement has length zero
Error in IPDB_matching_proteome$IPC_protein[i] <- isoelectricpointdb$IPC_protein[grep(pattern = paste("|",  : 
  replacement has length zero
Error in IPDB_matching_proteome$Sequence[i] <- as.character(isoelectricpointdb$sequence[grep(pattern = paste("|",  : 
  replacement has length zero
Error in IPDB_matching_proteome$MolWeight[i] <- isoelectricpointdb$molecular_weight[grep(pattern = paste("|",  : 
  replacement has length zero
Error in IPDB_matching_proteome$IPC_protein[i] <- isoelectricpointdb$IPC_protein[grep(pattern = paste("|",  : 
  replacement has length zero
Error in IPDB_matching_proteome$Sequence[i] <- as.character(isoelectricpointdb$sequence[grep(pattern = paste("|",  : 
  replacement has length zero
Error in IPDB_matching_proteome$MolWeight[i] <- isoelectricpointdb$molecular_weight[grep(pattern = paste("|",  : 
  replacement has length zero
Error in IPDB_matching_proteome$IPC_protein[i] <- isoelectricpointdb$IPC_protein[grep(pattern = paste("|",  : 
  replacement has length zero
Error in IPDB_matching_proteome$Sequence[i] <- as.character(isoelectricpointdb$sequence[grep(pattern = paste("|",  : 
  replacement has length zero
Error in IPDB_matching_proteome$MolWeight[i] <- isoelectricpointdb$molecular_weight[grep(pattern = paste("|",  : 
  replacement has length zero
Error in IPDB_matching_proteome$IPC_protein[i] <- isoelectricpointdb$IPC_protein[grep(pattern = paste("|",  : 
  replacement has length zero
Error in IPDB_matching_proteome$Sequence[i] <- as.character(isoelectricpointdb$sequence[grep(pattern = paste("|",  : 
  replacement has length zero
Error in IPDB_matching_proteome$MolWeight[i] <- isoelectricpointdb$molecular_weight[grep(pattern = paste("|",  : 
  replacement has length zero
Error in IPDB_matching_proteome$IPC_protein[i] <- isoelectricpointdb$IPC_protein[grep(pattern = paste("|",  : 
  replacement has length zero
Error in IPDB_matching_proteome$Sequence[i] <- as.character(isoelectricpointdb$sequence[grep(pattern = paste("|",  : 
  replacement has length zero
Error in IPDB_matching_proteome$MolWeight[i] <- isoelectricpointdb$molecular_weight[grep(pattern = paste("|",  : 
  replacement has length zero
Error in IPDB_matching_proteome$IPC_protein[i] <- isoelectricpointdb$IPC_protein[grep(pattern = paste("|",  : 
  replacement has length zero
Error in IPDB_matching_proteome$Sequence[i] <- as.character(isoelectricpointdb$sequence[grep(pattern = paste("|",  : 
  replacement has length zero
Error in IPDB_matching_proteome$MolWeight[i] <- isoelectricpointdb$molecular_weight[grep(pattern = paste("|",  : 
  replacement has length zero
Error in IPDB_matching_proteome$IPC_protein[i] <- isoelectricpointdb$IPC_protein[grep(pattern = paste("|",  : 
  replacement has length zero
Error in IPDB_matching_proteome$Sequence[i] <- as.character(isoelectricpointdb$sequence[grep(pattern = paste("|",  : 
  replacement has length zero
Error in IPDB_matching_proteome$MolWeight[i] <- isoelectricpointdb$molecular_weight[grep(pattern = paste("|",  : 
  replacement has length zero
Error in IPDB_matching_proteome$IPC_protein[i] <- isoelectricpointdb$IPC_protein[grep(pattern = paste("|",  : 
  replacement has length zero
Error in IPDB_matching_proteome$Sequence[i] <- as.character(isoelectricpointdb$sequence[grep(pattern = paste("|",  : 
  replacement has length zero
Error in IPDB_matching_proteome$MolWeight[i] <- isoelectricpointdb$molecular_weight[grep(pattern = paste("|",  : 
  replacement has length zero
Error in IPDB_matching_proteome$IPC_protein[i] <- isoelectricpointdb$IPC_protein[grep(pattern = paste("|",  : 
  replacement has length zero
Error in IPDB_matching_proteome$Sequence[i] <- as.character(isoelectricpointdb$sequence[grep(pattern = paste("|",  : 
  replacement has length zero
Error in IPDB_matching_proteome$MolWeight[i] <- isoelectricpointdb$molecular_weight[grep(pattern = paste("|",  : 
  replacement has length zero
Error in IPDB_matching_proteome$IPC_protein[i] <- isoelectricpointdb$IPC_protein[grep(pattern = paste("|",  : 
  replacement has length zero
Error in IPDB_matching_proteome$Sequence[i] <- as.character(isoelectricpointdb$sequence[grep(pattern = paste("|",  : 
  replacement has length zero
Error in IPDB_matching_proteome$MolWeight[i] <- isoelectricpointdb$molecular_weight[grep(pattern = paste("|",  : 
  replacement has length zero
Error in IPDB_matching_proteome$IPC_protein[i] <- isoelectricpointdb$IPC_protein[grep(pattern = paste("|",  : 
  replacement has length zero
Error in IPDB_matching_proteome$Sequence[i] <- as.character(isoelectricpointdb$sequence[grep(pattern = paste("|",  : 
  replacement has length zero
Error in IPDB_matching_proteome$MolWeight[i] <- isoelectricpointdb$molecular_weight[grep(pattern = paste("|",  : 
  replacement has length zero
Error in IPDB_matching_proteome$IPC_protein[i] <- isoelectricpointdb$IPC_protein[grep(pattern = paste("|",  : 
  replacement has length zero
Error in IPDB_matching_proteome$Sequence[i] <- as.character(isoelectricpointdb$sequence[grep(pattern = paste("|",  : 
  replacement has length zero
Error in IPDB_matching_proteome$MolWeight[i] <- isoelectricpointdb$molecular_weight[grep(pattern = paste("|",  : 
  replacement has length zero
Error in IPDB_matching_proteome$IPC_protein[i] <- isoelectricpointdb$IPC_protein[grep(pattern = paste("|",  : 
  replacement has length zero
Error in IPDB_matching_proteome$Sequence[i] <- as.character(isoelectricpointdb$sequence[grep(pattern = paste("|",  : 
  replacement has length zero
Error in IPDB_matching_proteome$MolWeight[i] <- isoelectricpointdb$molecular_weight[grep(pattern = paste("|",  : 
  replacement has length zero
Error in IPDB_matching_proteome$IPC_protein[i] <- isoelectricpointdb$IPC_protein[grep(pattern = paste("|",  : 
  replacement has length zero
Error in IPDB_matching_proteome$Sequence[i] <- as.character(isoelectricpointdb$sequence[grep(pattern = paste("|",  : 
  replacement has length zero
Error in IPDB_matching_proteome$MolWeight[i] <- isoelectricpointdb$molecular_weight[grep(pattern = paste("|",  : 
  replacement has length zero
Error in IPDB_matching_proteome$IPC_protein[i] <- isoelectricpointdb$IPC_protein[grep(pattern = paste("|",  : 
  replacement has length zero
Error in IPDB_matching_proteome$Sequence[i] <- as.character(isoelectricpointdb$sequence[grep(pattern = paste("|",  : 
  replacement has length zero
Error in IPDB_matching_proteome$MolWeight[i] <- isoelectricpointdb$molecular_weight[grep(pattern = paste("|",  : 
  replacement has length zero
Error in IPDB_matching_proteome$IPC_protein[i] <- isoelectricpointdb$IPC_protein[grep(pattern = paste("|",  : 
  replacement has length zero
Error in IPDB_matching_proteome$Sequence[i] <- as.character(isoelectricpointdb$sequence[grep(pattern = paste("|",  : 
  replacement has length zero
Error in IPDB_matching_proteome$MolWeight[i] <- isoelectricpointdb$molecular_weight[grep(pattern = paste("|",  : 
  replacement has length zero
Error in IPDB_matching_proteome$IPC_protein[i] <- isoelectricpointdb$IPC_protein[grep(pattern = paste("|",  : 
  replacement has length zero
Error in IPDB_matching_proteome$Sequence[i] <- as.character(isoelectricpointdb$sequence[grep(pattern = paste("|",  : 
  replacement has length zero
Error in IPDB_matching_proteome$MolWeight[i] <- isoelectricpointdb$molecular_weight[grep(pattern = paste("|",  : 
  replacement has length zero
Error in IPDB_matching_proteome$IPC_protein[i] <- isoelectricpointdb$IPC_protein[grep(pattern = paste("|",  : 
  replacement has length zero
Error in IPDB_matching_proteome$Sequence[i] <- as.character(isoelectricpointdb$sequence[grep(pattern = paste("|",  : 
  replacement has length zero
Error in IPDB_matching_proteome$MolWeight[i] <- isoelectricpointdb$molecular_weight[grep(pattern = paste("|",  : 
  replacement has length zero
Error in IPDB_matching_proteome$IPC_protein[i] <- isoelectricpointdb$IPC_protein[grep(pattern = paste("|",  : 
  replacement has length zero
Error in IPDB_matching_proteome$Sequence[i] <- as.character(isoelectricpointdb$sequence[grep(pattern = paste("|",  : 
  replacement has length zero
Error in IPDB_matching_proteome$MolWeight[i] <- isoelectricpointdb$molecular_weight[grep(pattern = paste("|",  : 
  replacement has length zero
Error in IPDB_matching_proteome$IPC_protein[i] <- isoelectricpointdb$IPC_protein[grep(pattern = paste("|",  : 
  replacement has length zero
Error in IPDB_matching_proteome$Sequence[i] <- as.character(isoelectricpointdb$sequence[grep(pattern = paste("|",  : 
  replacement has length zero
Error in IPDB_matching_proteome$MolWeight[i] <- isoelectricpointdb$molecular_weight[grep(pattern = paste("|",  : 
  replacement has length zero
Error in IPDB_matching_proteome$IPC_protein[i] <- isoelectricpointdb$IPC_protein[grep(pattern = paste("|",  : 
  replacement has length zero
Error in IPDB_matching_proteome$Sequence[i] <- as.character(isoelectricpointdb$sequence[grep(pattern = paste("|",  : 
  replacement has length zero
Error in IPDB_matching_proteome$MolWeight[i] <- isoelectricpointdb$molecular_weight[grep(pattern = paste("|",  : 
  replacement has length zero
Error in IPDB_matching_proteome$IPC_protein[i] <- isoelectricpointdb$IPC_protein[grep(pattern = paste("|",  : 
  replacement has length zero
Error in IPDB_matching_proteome$Sequence[i] <- as.character(isoelectricpointdb$sequence[grep(pattern = paste("|",  : 
  replacement has length zero
Error in IPDB_matching_proteome$MolWeight[i] <- isoelectricpointdb$molecular_weight[grep(pattern = paste("|",  : 
  replacement has length zero
Error in IPDB_matching_proteome$IPC_protein[i] <- isoelectricpointdb$IPC_protein[grep(pattern = paste("|",  : 
  replacement has length zero
Error in IPDB_matching_proteome$Sequence[i] <- as.character(isoelectricpointdb$sequence[grep(pattern = paste("|",  : 
  replacement has length zero
Error in IPDB_matching_proteome$MolWeight[i] <- isoelectricpointdb$molecular_weight[grep(pattern = paste("|",  : 
  replacement has length zero
Error in IPDB_matching_proteome$IPC_protein[i] <- isoelectricpointdb$IPC_protein[grep(pattern = paste("|",  : 
  replacement has length zero
Error in IPDB_matching_proteome$Sequence[i] <- as.character(isoelectricpointdb$sequence[grep(pattern = paste("|",  : 
  replacement has length zero
Error in IPDB_matching_proteome$MolWeight[i] <- isoelectricpointdb$molecular_weight[grep(pattern = paste("|",  : 
  replacement has length zero
Error in IPDB_matching_proteome$IPC_protein[i] <- isoelectricpointdb$IPC_protein[grep(pattern = paste("|",  : 
  replacement has length zero
Error in IPDB_matching_proteome$Sequence[i] <- as.character(isoelectricpointdb$sequence[grep(pattern = paste("|",  : 
  replacement has length zero
Error in IPDB_matching_proteome$MolWeight[i] <- isoelectricpointdb$molecular_weight[grep(pattern = paste("|",  : 
  replacement has length zero
Error in IPDB_matching_proteome$IPC_protein[i] <- isoelectricpointdb$IPC_protein[grep(pattern = paste("|",  : 
  replacement has length zero
Error in IPDB_matching_proteome$Sequence[i] <- as.character(isoelectricpointdb$sequence[grep(pattern = paste("|",  : 
  replacement has length zero
Error in IPDB_matching_proteome$MolWeight[i] <- isoelectricpointdb$molecular_weight[grep(pattern = paste("|",  : 
  replacement has length zero
Error in IPDB_matching_proteome$IPC_protein[i] <- isoelectricpointdb$IPC_protein[grep(pattern = paste("|",  : 
  replacement has length zero
Error in IPDB_matching_proteome$Sequence[i] <- as.character(isoelectricpointdb$sequence[grep(pattern = paste("|",  : 
  replacement has length zero
Error in IPDB_matching_proteome$MolWeight[i] <- isoelectricpointdb$molecular_weight[grep(pattern = paste("|",  : 
  replacement has length zero
Error in IPDB_matching_proteome$IPC_protein[i] <- isoelectricpointdb$IPC_protein[grep(pattern = paste("|",  : 
  replacement has length zero
Error in IPDB_matching_proteome$Sequence[i] <- as.character(isoelectricpointdb$sequence[grep(pattern = paste("|",  : 
  replacement has length zero
Error in IPDB_matching_proteome$MolWeight[i] <- isoelectricpointdb$molecular_weight[grep(pattern = paste("|",  : 
  replacement has length zero
Error in IPDB_matching_proteome$IPC_protein[i] <- isoelectricpointdb$IPC_protein[grep(pattern = paste("|",  : 
  replacement has length zero
Error in IPDB_matching_proteome$Sequence[i] <- as.character(isoelectricpointdb$sequence[grep(pattern = paste("|",  : 
  replacement has length zero
Error in IPDB_matching_proteome$MolWeight[i] <- isoelectricpointdb$molecular_weight[grep(pattern = paste("|",  : 
  replacement has length zero
Error in IPDB_matching_proteome$IPC_protein[i] <- isoelectricpointdb$IPC_protein[grep(pattern = paste("|",  : 
  replacement has length zero
Error in IPDB_matching_proteome$Sequence[i] <- as.character(isoelectricpointdb$sequence[grep(pattern = paste("|",  : 
  replacement has length zero
Error in IPDB_matching_proteome$MolWeight[i] <- isoelectricpointdb$molecular_weight[grep(pattern = paste("|",  : 
  replacement has length zero
Error in IPDB_matching_proteome$IPC_protein[i] <- isoelectricpointdb$IPC_protein[grep(pattern = paste("|",  : 
  replacement has length zero
Error in IPDB_matching_proteome$Sequence[i] <- as.character(isoelectricpointdb$sequence[grep(pattern = paste("|",  : 
  replacement has length zero
Error in IPDB_matching_proteome$MolWeight[i] <- isoelectricpointdb$molecular_weight[grep(pattern = paste("|",  : 
  replacement has length zero
Error in IPDB_matching_proteome$IPC_protein[i] <- isoelectricpointdb$IPC_protein[grep(pattern = paste("|",  : 
  replacement has length zero
Error in IPDB_matching_proteome$Sequence[i] <- as.character(isoelectricpointdb$sequence[grep(pattern = paste("|",  : 
  replacement has length zero
Error in IPDB_matching_proteome$MolWeight[i] <- isoelectricpointdb$molecular_weight[grep(pattern = paste("|",  : 
  replacement has length zero
Error in IPDB_matching_proteome$IPC_protein[i] <- isoelectricpointdb$IPC_protein[grep(pattern = paste("|",  : 
  replacement has length zero
Error in IPDB_matching_proteome$Sequence[i] <- as.character(isoelectricpointdb$sequence[grep(pattern = paste("|",  : 
  replacement has length zero
Error in IPDB_matching_proteome$MolWeight[i] <- isoelectricpointdb$molecular_weight[grep(pattern = paste("|",  : 
  replacement has length zero
Error in IPDB_matching_proteome$IPC_protein[i] <- isoelectricpointdb$IPC_protein[grep(pattern = paste("|",  : 
  replacement has length zero
Error in IPDB_matching_proteome$Sequence[i] <- as.character(isoelectricpointdb$sequence[grep(pattern = paste("|",  : 
  replacement has length zero
Error in IPDB_matching_proteome$MolWeight[i] <- isoelectricpointdb$molecular_weight[grep(pattern = paste("|",  : 
  replacement has length zero
Error in IPDB_matching_proteome$IPC_protein[i] <- isoelectricpointdb$IPC_protein[grep(pattern = paste("|",  : 
  replacement has length zero
Error in IPDB_matching_proteome$Sequence[i] <- as.character(isoelectricpointdb$sequence[grep(pattern = paste("|",  : 
  replacement has length zero
Error in IPDB_matching_proteome$MolWeight[i] <- isoelectricpointdb$molecular_weight[grep(pattern = paste("|",  : 
  replacement has length zero
Error in IPDB_matching_proteome$IPC_protein[i] <- isoelectricpointdb$IPC_protein[grep(pattern = paste("|",  : 
  replacement has length zero
Error in IPDB_matching_proteome$Sequence[i] <- as.character(isoelectricpointdb$sequence[grep(pattern = paste("|",  : 
  replacement has length zero
Error in IPDB_matching_proteome$MolWeight[i] <- isoelectricpointdb$molecular_weight[grep(pattern = paste("|",  : 
  replacement has length zero
Error in IPDB_matching_proteome$IPC_protein[i] <- isoelectricpointdb$IPC_protein[grep(pattern = paste("|",  : 
  replacement has length zero
Error in IPDB_matching_proteome$Sequence[i] <- as.character(isoelectricpointdb$sequence[grep(pattern = paste("|",  : 
  replacement has length zero
Error in IPDB_matching_proteome$MolWeight[i] <- isoelectricpointdb$molecular_weight[grep(pattern = paste("|",  : 
  replacement has length zero
Error in IPDB_matching_proteome$IPC_protein[i] <- isoelectricpointdb$IPC_protein[grep(pattern = paste("|",  : 
  replacement has length zero
Error in IPDB_matching_proteome$Sequence[i] <- as.character(isoelectricpointdb$sequence[grep(pattern = paste("|",  : 
  replacement has length zero
Error in IPDB_matching_proteome$MolWeight[i] <- isoelectricpointdb$molecular_weight[grep(pattern = paste("|",  : 
  replacement has length zero
Error in IPDB_matching_proteome$IPC_protein[i] <- isoelectricpointdb$IPC_protein[grep(pattern = paste("|",  : 
  replacement has length zero
Error in IPDB_matching_proteome$Sequence[i] <- as.character(isoelectricpointdb$sequence[grep(pattern = paste("|",  : 
  replacement has length zero
Error in IPDB_matching_proteome$MolWeight[i] <- isoelectricpointdb$molecular_weight[grep(pattern = paste("|",  : 
  replacement has length zero
Error in IPDB_matching_proteome$IPC_protein[i] <- isoelectricpointdb$IPC_protein[grep(pattern = paste("|",  : 
  replacement has length zero
Error in IPDB_matching_proteome$Sequence[i] <- as.character(isoelectricpointdb$sequence[grep(pattern = paste("|",  : 
  replacement has length zero
Error in IPDB_matching_proteome$MolWeight[i] <- isoelectricpointdb$molecular_weight[grep(pattern = paste("|",  : 
  replacement has length zero
Error in IPDB_matching_proteome$IPC_protein[i] <- isoelectricpointdb$IPC_protein[grep(pattern = paste("|",  : 
  replacement has length zero
Error in IPDB_matching_proteome$Sequence[i] <- as.character(isoelectricpointdb$sequence[grep(pattern = paste("|",  : 
  replacement has length zero
Error in IPDB_matching_proteome$MolWeight[i] <- isoelectricpointdb$molecular_weight[grep(pattern = paste("|",  : 
  replacement has length zero
Error in IPDB_matching_proteome$IPC_protein[i] <- isoelectricpointdb$IPC_protein[grep(pattern = paste("|",  : 
  replacement has length zero
Error in IPDB_matching_proteome$Sequence[i] <- as.character(isoelectricpointdb$sequence[grep(pattern = paste("|",  : 
  replacement has length zero
Error in IPDB_matching_proteome$MolWeight[i] <- isoelectricpointdb$molecular_weight[grep(pattern = paste("|",  : 
  replacement has length zero
Error in IPDB_matching_proteome$IPC_protein[i] <- isoelectricpointdb$IPC_protein[grep(pattern = paste("|",  : 
  replacement has length zero
Error in IPDB_matching_proteome$Sequence[i] <- as.character(isoelectricpointdb$sequence[grep(pattern = paste("|",  : 
  replacement has length zero
Error in IPDB_matching_proteome$MolWeight[i] <- isoelectricpointdb$molecular_weight[grep(pattern = paste("|",  : 
  replacement has length zero
Error in IPDB_matching_proteome$IPC_protein[i] <- isoelectricpointdb$IPC_protein[grep(pattern = paste("|",  : 
  replacement has length zero
Error in IPDB_matching_proteome$Sequence[i] <- as.character(isoelectricpointdb$sequence[grep(pattern = paste("|",  : 
  replacement has length zero
Error in IPDB_matching_proteome$MolWeight[i] <- isoelectricpointdb$molecular_weight[grep(pattern = paste("|",  : 
  replacement has length zero
Error in IPDB_matching_proteome$IPC_protein[i] <- isoelectricpointdb$IPC_protein[grep(pattern = paste("|",  : 
  replacement has length zero
Error in IPDB_matching_proteome$Sequence[i] <- as.character(isoelectricpointdb$sequence[grep(pattern = paste("|",  : 
  replacement has length zero
Error in IPDB_matching_proteome$MolWeight[i] <- isoelectricpointdb$molecular_weight[grep(pattern = paste("|",  : 
  replacement has length zero
Error in IPDB_matching_proteome$IPC_protein[i] <- isoelectricpointdb$IPC_protein[grep(pattern = paste("|",  : 
  replacement has length zero
Error in IPDB_matching_proteome$Sequence[i] <- as.character(isoelectricpointdb$sequence[grep(pattern = paste("|",  : 
  replacement has length zero
Error in IPDB_matching_proteome$MolWeight[i] <- isoelectricpointdb$molecular_weight[grep(pattern = paste("|",  : 
  replacement has length zero
Error in IPDB_matching_proteome$IPC_protein[i] <- isoelectricpointdb$IPC_protein[grep(pattern = paste("|",  : 
  replacement has length zero
Error in IPDB_matching_proteome$Sequence[i] <- as.character(isoelectricpointdb$sequence[grep(pattern = paste("|",  : 
  replacement has length zero
Error in IPDB_matching_proteome$MolWeight[i] <- isoelectricpointdb$molecular_weight[grep(pattern = paste("|",  : 
  replacement has length zero
Error in IPDB_matching_proteome$IPC_protein[i] <- isoelectricpointdb$IPC_protein[grep(pattern = paste("|",  : 
  replacement has length zero
Error in IPDB_matching_proteome$Sequence[i] <- as.character(isoelectricpointdb$sequence[grep(pattern = paste("|",  : 
  replacement has length zero
Error in IPDB_matching_proteome$MolWeight[i] <- isoelectricpointdb$molecular_weight[grep(pattern = paste("|",  : 
  replacement has length zero
Error in IPDB_matching_proteome$IPC_protein[i] <- isoelectricpointdb$IPC_protein[grep(pattern = paste("|",  : 
  replacement has length zero
Error in IPDB_matching_proteome$Sequence[i] <- as.character(isoelectricpointdb$sequence[grep(pattern = paste("|",  : 
  replacement has length zero
Error in IPDB_matching_proteome$MolWeight[i] <- isoelectricpointdb$molecular_weight[grep(pattern = paste("|",  : 
  replacement has length zero
Error in IPDB_matching_proteome$IPC_protein[i] <- isoelectricpointdb$IPC_protein[grep(pattern = paste("|",  : 
  replacement has length zero
Error in IPDB_matching_proteome$Sequence[i] <- as.character(isoelectricpointdb$sequence[grep(pattern = paste("|",  : 
  replacement has length zero
Error in IPDB_matching_proteome$MolWeight[i] <- isoelectricpointdb$molecular_weight[grep(pattern = paste("|",  : 
  replacement has length zero
Error in IPDB_matching_proteome$IPC_protein[i] <- isoelectricpointdb$IPC_protein[grep(pattern = paste("|",  : 
  replacement has length zero
Error in IPDB_matching_proteome$Sequence[i] <- as.character(isoelectricpointdb$sequence[grep(pattern = paste("|",  : 
  replacement has length zero
Error in IPDB_matching_proteome$MolWeight[i] <- isoelectricpointdb$molecular_weight[grep(pattern = paste("|",  : 
  replacement has length zero
Error in IPDB_matching_proteome$IPC_protein[i] <- isoelectricpointdb$IPC_protein[grep(pattern = paste("|",  : 
  replacement has length zero
Error in IPDB_matching_proteome$Sequence[i] <- as.character(isoelectricpointdb$sequence[grep(pattern = paste("|",  : 
  replacement has length zero
Error in IPDB_matching_proteome$MolWeight[i] <- isoelectricpointdb$molecular_weight[grep(pattern = paste("|",  : 
  replacement has length zero
Error in IPDB_matching_proteome$IPC_protein[i] <- isoelectricpointdb$IPC_protein[grep(pattern = paste("|",  : 
  replacement has length zero
Error in IPDB_matching_proteome$Sequence[i] <- as.character(isoelectricpointdb$sequence[grep(pattern = paste("|",  : 
  replacement has length zero
Error in IPDB_matching_proteome$MolWeight[i] <- isoelectricpointdb$molecular_weight[grep(pattern = paste("|",  : 
  replacement has length zero
Error in IPDB_matching_proteome$IPC_protein[i] <- isoelectricpointdb$IPC_protein[grep(pattern = paste("|",  : 
  replacement has length zero
Error in IPDB_matching_proteome$Sequence[i] <- as.character(isoelectricpointdb$sequence[grep(pattern = paste("|",  : 
  replacement has length zero
Error in IPDB_matching_proteome$MolWeight[i] <- isoelectricpointdb$molecular_weight[grep(pattern = paste("|",  : 
  replacement has length zero
Error in IPDB_matching_proteome$IPC_protein[i] <- isoelectricpointdb$IPC_protein[grep(pattern = paste("|",  : 
  replacement has length zero
Error in IPDB_matching_proteome$Sequence[i] <- as.character(isoelectricpointdb$sequence[grep(pattern = paste("|",  : 
  replacement has length zero
Error in IPDB_matching_proteome$MolWeight[i] <- isoelectricpointdb$molecular_weight[grep(pattern = paste("|",  : 
  replacement has length zero
Error in IPDB_matching_proteome$IPC_protein[i] <- isoelectricpointdb$IPC_protein[grep(pattern = paste("|",  : 
  replacement has length zero
Error in IPDB_matching_proteome$Sequence[i] <- as.character(isoelectricpointdb$sequence[grep(pattern = paste("|",  : 
  replacement has length zero
Error in IPDB_matching_proteome$MolWeight[i] <- isoelectricpointdb$molecular_weight[grep(pattern = paste("|",  : 
  replacement has length zero
Error in IPDB_matching_proteome$IPC_protein[i] <- isoelectricpointdb$IPC_protein[grep(pattern = paste("|",  : 
  replacement has length zero
Error in IPDB_matching_proteome$Sequence[i] <- as.character(isoelectricpointdb$sequence[grep(pattern = paste("|",  : 
  replacement has length zero
Error in IPDB_matching_proteome$MolWeight[i] <- isoelectricpointdb$molecular_weight[grep(pattern = paste("|",  : 
  replacement has length zero
Error in IPDB_matching_proteome$IPC_protein[i] <- isoelectricpointdb$IPC_protein[grep(pattern = paste("|",  : 
  replacement has length zero
Error in IPDB_matching_proteome$Sequence[i] <- as.character(isoelectricpointdb$sequence[grep(pattern = paste("|",  : 
  replacement has length zero
Error in IPDB_matching_proteome$MolWeight[i] <- isoelectricpointdb$molecular_weight[grep(pattern = paste("|",  : 
  replacement has length zero
Error in IPDB_matching_proteome$IPC_protein[i] <- isoelectricpointdb$IPC_protein[grep(pattern = paste("|",  : 
  replacement has length zero
Error in IPDB_matching_proteome$Sequence[i] <- as.character(isoelectricpointdb$sequence[grep(pattern = paste("|",  : 
  replacement has length zero
Error in IPDB_matching_proteome$MolWeight[i] <- isoelectricpointdb$molecular_weight[grep(pattern = paste("|",  : 
  replacement has length zero
Error in IPDB_matching_proteome$IPC_protein[i] <- isoelectricpointdb$IPC_protein[grep(pattern = paste("|",  : 
  replacement has length zero
Error in IPDB_matching_proteome$Sequence[i] <- as.character(isoelectricpointdb$sequence[grep(pattern = paste("|",  : 
  replacement has length zero
Error in IPDB_matching_proteome$MolWeight[i] <- isoelectricpointdb$molecular_weight[grep(pattern = paste("|",  : 
  replacement has length zero
Error in IPDB_matching_proteome$IPC_protein[i] <- isoelectricpointdb$IPC_protein[grep(pattern = paste("|",  : 
  replacement has length zero
Error in IPDB_matching_proteome$Sequence[i] <- as.character(isoelectricpointdb$sequence[grep(pattern = paste("|",  : 
  replacement has length zero
Error in IPDB_matching_proteome$MolWeight[i] <- isoelectricpointdb$molecular_weight[grep(pattern = paste("|",  : 
  replacement has length zero
Error in IPDB_matching_proteome$IPC_protein[i] <- isoelectricpointdb$IPC_protein[grep(pattern = paste("|",  : 
  replacement has length zero
Error in IPDB_matching_proteome$Sequence[i] <- as.character(isoelectricpointdb$sequence[grep(pattern = paste("|",  : 
  replacement has length zero
Error in IPDB_matching_proteome$MolWeight[i] <- isoelectricpointdb$molecular_weight[grep(pattern = paste("|",  : 
  replacement has length zero
Error in IPDB_matching_proteome$IPC_protein[i] <- isoelectricpointdb$IPC_protein[grep(pattern = paste("|",  : 
  replacement has length zero
Error in IPDB_matching_proteome$Sequence[i] <- as.character(isoelectricpointdb$sequence[grep(pattern = paste("|",  : 
  replacement has length zero
Error in IPDB_matching_proteome$MolWeight[i] <- isoelectricpointdb$molecular_weight[grep(pattern = paste("|",  : 
  replacement has length zero
Error in IPDB_matching_proteome$IPC_protein[i] <- isoelectricpointdb$IPC_protein[grep(pattern = paste("|",  : 
  replacement has length zero
Error in IPDB_matching_proteome$Sequence[i] <- as.character(isoelectricpointdb$sequence[grep(pattern = paste("|",  : 
  replacement has length zero
Error in IPDB_matching_proteome$MolWeight[i] <- isoelectricpointdb$molecular_weight[grep(pattern = paste("|",  : 
  replacement has length zero
Error in IPDB_matching_proteome$IPC_protein[i] <- isoelectricpointdb$IPC_protein[grep(pattern = paste("|",  : 
  replacement has length zero
Error in IPDB_matching_proteome$Sequence[i] <- as.character(isoelectricpointdb$sequence[grep(pattern = paste("|",  : 
  replacement has length zero
Error in IPDB_matching_proteome$MolWeight[i] <- isoelectricpointdb$molecular_weight[grep(pattern = paste("|",  : 
  replacement has length zero
Error in IPDB_matching_proteome$IPC_protein[i] <- isoelectricpointdb$IPC_protein[grep(pattern = paste("|",  : 
  replacement has length zero
Error in IPDB_matching_proteome$Sequence[i] <- as.character(isoelectricpointdb$sequence[grep(pattern = paste("|",  : 
  replacement has length zero
Error in IPDB_matching_proteome$MolWeight[i] <- isoelectricpointdb$molecular_weight[grep(pattern = paste("|",  : 
  replacement has length zero
Error in IPDB_matching_proteome$IPC_protein[i] <- isoelectricpointdb$IPC_protein[grep(pattern = paste("|",  : 
  replacement has length zero
Error in IPDB_matching_proteome$Sequence[i] <- as.character(isoelectricpointdb$sequence[grep(pattern = paste("|",  : 
  replacement has length zero
Error in IPDB_matching_proteome$MolWeight[i] <- isoelectricpointdb$molecular_weight[grep(pattern = paste("|",  : 
  replacement has length zero
Error in IPDB_matching_proteome$IPC_protein[i] <- isoelectricpointdb$IPC_protein[grep(pattern = paste("|",  : 
  replacement has length zero
Error in IPDB_matching_proteome$Sequence[i] <- as.character(isoelectricpointdb$sequence[grep(pattern = paste("|",  : 
  replacement has length zero
Error in IPDB_matching_proteome$MolWeight[i] <- isoelectricpointdb$molecular_weight[grep(pattern = paste("|",  : 
  replacement has length zero
Error in IPDB_matching_proteome$IPC_protein[i] <- isoelectricpointdb$IPC_protein[grep(pattern = paste("|",  : 
  replacement has length zero
Error in IPDB_matching_proteome$Sequence[i] <- as.character(isoelectricpointdb$sequence[grep(pattern = paste("|",  : 
  replacement has length zero
Error in IPDB_matching_proteome$MolWeight[i] <- isoelectricpointdb$molecular_weight[grep(pattern = paste("|",  : 
  replacement has length zero
Error in IPDB_matching_proteome$IPC_protein[i] <- isoelectricpointdb$IPC_protein[grep(pattern = paste("|",  : 
  replacement has length zero
Error in IPDB_matching_proteome$Sequence[i] <- as.character(isoelectricpointdb$sequence[grep(pattern = paste("|",  : 
  replacement has length zero
Error in IPDB_matching_proteome$MolWeight[i] <- isoelectricpointdb$molecular_weight[grep(pattern = paste("|",  : 
  replacement has length zero
Error in IPDB_matching_proteome$IPC_protein[i] <- isoelectricpointdb$IPC_protein[grep(pattern = paste("|",  : 
  replacement has length zero
Error in IPDB_matching_proteome$Sequence[i] <- as.character(isoelectricpointdb$sequence[grep(pattern = paste("|",  : 
  replacement has length zero
Error in IPDB_matching_proteome$MolWeight[i] <- isoelectricpointdb$molecular_weight[grep(pattern = paste("|",  : 
  replacement has length zero
Error in IPDB_matching_proteome$IPC_protein[i] <- isoelectricpointdb$IPC_protein[grep(pattern = paste("|",  : 
  replacement has length zero
Error in IPDB_matching_proteome$Sequence[i] <- as.character(isoelectricpointdb$sequence[grep(pattern = paste("|",  : 
  replacement has length zero
Error in IPDB_matching_proteome$MolWeight[i] <- isoelectricpointdb$molecular_weight[grep(pattern = paste("|",  : 
  replacement has length zero
Error in IPDB_matching_proteome$IPC_protein[i] <- isoelectricpointdb$IPC_protein[grep(pattern = paste("|",  : 
  replacement has length zero
Error in IPDB_matching_proteome$Sequence[i] <- as.character(isoelectricpointdb$sequence[grep(pattern = paste("|",  : 
  replacement has length zero
Error in IPDB_matching_proteome$MolWeight[i] <- isoelectricpointdb$molecular_weight[grep(pattern = paste("|",  : 
  replacement has length zero
Error in IPDB_matching_proteome$IPC_protein[i] <- isoelectricpointdb$IPC_protein[grep(pattern = paste("|",  : 
  replacement has length zero
Error in IPDB_matching_proteome$Sequence[i] <- as.character(isoelectricpointdb$sequence[grep(pattern = paste("|",  : 
  replacement has length zero
Error in IPDB_matching_proteome$MolWeight[i] <- isoelectricpointdb$molecular_weight[grep(pattern = paste("|",  : 
  replacement has length zero
Error in IPDB_matching_proteome$IPC_protein[i] <- isoelectricpointdb$IPC_protein[grep(pattern = paste("|",  : 
  replacement has length zero
Error in IPDB_matching_proteome$Sequence[i] <- as.character(isoelectricpointdb$sequence[grep(pattern = paste("|",  : 
  replacement has length zero
Error in IPDB_matching_proteome$MolWeight[i] <- isoelectricpointdb$molecular_weight[grep(pattern = paste("|",  : 
  replacement has length zero
Error in IPDB_matching_proteome$IPC_protein[i] <- isoelectricpointdb$IPC_protein[grep(pattern = paste("|",  : 
  replacement has length zero
Error in IPDB_matching_proteome$Sequence[i] <- as.character(isoelectricpointdb$sequence[grep(pattern = paste("|",  : 
  replacement has length zero
Error in IPDB_matching_proteome$MolWeight[i] <- isoelectricpointdb$molecular_weight[grep(pattern = paste("|",  : 
  replacement has length zero
Error in IPDB_matching_proteome$IPC_protein[i] <- isoelectricpointdb$IPC_protein[grep(pattern = paste("|",  : 
  replacement has length zero
Error in IPDB_matching_proteome$Sequence[i] <- as.character(isoelectricpointdb$sequence[grep(pattern = paste("|",  : 
  replacement has length zero
Error in IPDB_matching_proteome$MolWeight[i] <- isoelectricpointdb$molecular_weight[grep(pattern = paste("|",  : 
  replacement has length zero
Error in IPDB_matching_proteome$IPC_protein[i] <- isoelectricpointdb$IPC_protein[grep(pattern = paste("|",  : 
  replacement has length zero
Error in IPDB_matching_proteome$Sequence[i] <- as.character(isoelectricpointdb$sequence[grep(pattern = paste("|",  : 
  replacement has length zero
Error in IPDB_matching_proteome$MolWeight[i] <- isoelectricpointdb$molecular_weight[grep(pattern = paste("|",  : 
  replacement has length zero
Error in IPDB_matching_proteome$IPC_protein[i] <- isoelectricpointdb$IPC_protein[grep(pattern = paste("|",  : 
  replacement has length zero
Error in IPDB_matching_proteome$Sequence[i] <- as.character(isoelectricpointdb$sequence[grep(pattern = paste("|",  : 
  replacement has length zero
Error in IPDB_matching_proteome$MolWeight[i] <- isoelectricpointdb$molecular_weight[grep(pattern = paste("|",  : 
  replacement has length zero
Error in IPDB_matching_proteome$IPC_protein[i] <- isoelectricpointdb$IPC_protein[grep(pattern = paste("|",  : 
  replacement has length zero
Error in IPDB_matching_proteome$Sequence[i] <- as.character(isoelectricpointdb$sequence[grep(pattern = paste("|",  : 
  replacement has length zero
Error in IPDB_matching_proteome$MolWeight[i] <- isoelectricpointdb$molecular_weight[grep(pattern = paste("|",  : 
  replacement has length zero
Error in IPDB_matching_proteome$IPC_protein[i] <- isoelectricpointdb$IPC_protein[grep(pattern = paste("|",  : 
  replacement has length zero
Error in IPDB_matching_proteome$Sequence[i] <- as.character(isoelectricpointdb$sequence[grep(pattern = paste("|",  : 
  replacement has length zero
Error in IPDB_matching_proteome$MolWeight[i] <- isoelectricpointdb$molecular_weight[grep(pattern = paste("|",  : 
  replacement has length zero
Error in IPDB_matching_proteome$IPC_protein[i] <- isoelectricpointdb$IPC_protein[grep(pattern = paste("|",  : 
  replacement has length zero
Error in IPDB_matching_proteome$Sequence[i] <- as.character(isoelectricpointdb$sequence[grep(pattern = paste("|",  : 
  replacement has length zero
Error in IPDB_matching_proteome$MolWeight[i] <- isoelectricpointdb$molecular_weight[grep(pattern = paste("|",  : 
  replacement has length zero
Error in IPDB_matching_proteome$IPC_protein[i] <- isoelectricpointdb$IPC_protein[grep(pattern = paste("|",  : 
  replacement has length zero
Error in IPDB_matching_proteome$Sequence[i] <- as.character(isoelectricpointdb$sequence[grep(pattern = paste("|",  : 
  replacement has length zero
Error in IPDB_matching_proteome$MolWeight[i] <- isoelectricpointdb$molecular_weight[grep(pattern = paste("|",  : 
  replacement has length zero
Error in IPDB_matching_proteome$IPC_protein[i] <- isoelectricpointdb$IPC_protein[grep(pattern = paste("|",  : 
  replacement has length zero
Error in IPDB_matching_proteome$Sequence[i] <- as.character(isoelectricpointdb$sequence[grep(pattern = paste("|",  : 
  replacement has length zero
Error in IPDB_matching_proteome$MolWeight[i] <- isoelectricpointdb$molecular_weight[grep(pattern = paste("|",  : 
  replacement has length zero
Error in IPDB_matching_proteome$IPC_protein[i] <- isoelectricpointdb$IPC_protein[grep(pattern = paste("|",  : 
  replacement has length zero
Error in IPDB_matching_proteome$Sequence[i] <- as.character(isoelectricpointdb$sequence[grep(pattern = paste("|",  : 
  replacement has length zero
Error in IPDB_matching_proteome$MolWeight[i] <- isoelectricpointdb$molecular_weight[grep(pattern = paste("|",  : 
  replacement has length zero
Error in IPDB_matching_proteome$IPC_protein[i] <- isoelectricpointdb$IPC_protein[grep(pattern = paste("|",  : 
  replacement has length zero
Error in IPDB_matching_proteome$Sequence[i] <- as.character(isoelectricpointdb$sequence[grep(pattern = paste("|",  : 
  replacement has length zero
Error in IPDB_matching_proteome$MolWeight[i] <- isoelectricpointdb$molecular_weight[grep(pattern = paste("|",  : 
  replacement has length zero
Error in IPDB_matching_proteome$IPC_protein[i] <- isoelectricpointdb$IPC_protein[grep(pattern = paste("|",  : 
  replacement has length zero
Error in IPDB_matching_proteome$Sequence[i] <- as.character(isoelectricpointdb$sequence[grep(pattern = paste("|",  : 
  replacement has length zero
Error in IPDB_matching_proteome$MolWeight[i] <- isoelectricpointdb$molecular_weight[grep(pattern = paste("|",  : 
  replacement has length zero
Error in IPDB_matching_proteome$IPC_protein[i] <- isoelectricpointdb$IPC_protein[grep(pattern = paste("|",  : 
  replacement has length zero
Error in IPDB_matching_proteome$Sequence[i] <- as.character(isoelectricpointdb$sequence[grep(pattern = paste("|",  : 
  replacement has length zero
Error in IPDB_matching_proteome$MolWeight[i] <- isoelectricpointdb$molecular_weight[grep(pattern = paste("|",  : 
  replacement has length zero
Error in IPDB_matching_proteome$IPC_protein[i] <- isoelectricpointdb$IPC_protein[grep(pattern = paste("|",  : 
  replacement has length zero
Error in IPDB_matching_proteome$Sequence[i] <- as.character(isoelectricpointdb$sequence[grep(pattern = paste("|",  : 
  replacement has length zero
Error in IPDB_matching_proteome$MolWeight[i] <- isoelectricpointdb$molecular_weight[grep(pattern = paste("|",  : 
  replacement has length zero
Error in IPDB_matching_proteome$IPC_protein[i] <- isoelectricpointdb$IPC_protein[grep(pattern = paste("|",  : 
  replacement has length zero
Error in IPDB_matching_proteome$Sequence[i] <- as.character(isoelectricpointdb$sequence[grep(pattern = paste("|",  : 
  replacement has length zero
Error in IPDB_matching_proteome$MolWeight[i] <- isoelectricpointdb$molecular_weight[grep(pattern = paste("|",  : 
  replacement has length zero
Error in IPDB_matching_proteome$IPC_protein[i] <- isoelectricpointdb$IPC_protein[grep(pattern = paste("|",  : 
  replacement has length zero
Error in IPDB_matching_proteome$Sequence[i] <- as.character(isoelectricpointdb$sequence[grep(pattern = paste("|",  : 
  replacement has length zero
Error in IPDB_matching_proteome$MolWeight[i] <- isoelectricpointdb$molecular_weight[grep(pattern = paste("|",  : 
  replacement has length zero
Error in IPDB_matching_proteome$IPC_protein[i] <- isoelectricpointdb$IPC_protein[grep(pattern = paste("|",  : 
  replacement has length zero
Error in IPDB_matching_proteome$Sequence[i] <- as.character(isoelectricpointdb$sequence[grep(pattern = paste("|",  : 
  replacement has length zero
Error in IPDB_matching_proteome$MolWeight[i] <- isoelectricpointdb$molecular_weight[grep(pattern = paste("|",  : 
  replacement has length zero
Error in IPDB_matching_proteome$IPC_protein[i] <- isoelectricpointdb$IPC_protein[grep(pattern = paste("|",  : 
  replacement has length zero
Error in IPDB_matching_proteome$Sequence[i] <- as.character(isoelectricpointdb$sequence[grep(pattern = paste("|",  : 
  replacement has length zero
Error in IPDB_matching_proteome$MolWeight[i] <- isoelectricpointdb$molecular_weight[grep(pattern = paste("|",  : 
  replacement has length zero
Error in IPDB_matching_proteome$IPC_protein[i] <- isoelectricpointdb$IPC_protein[grep(pattern = paste("|",  : 
  replacement has length zero
Error in IPDB_matching_proteome$Sequence[i] <- as.character(isoelectricpointdb$sequence[grep(pattern = paste("|",  : 
  replacement has length zero
Error in IPDB_matching_proteome$MolWeight[i] <- isoelectricpointdb$molecular_weight[grep(pattern = paste("|",  : 
  replacement has length zero
Error in IPDB_matching_proteome$IPC_protein[i] <- isoelectricpointdb$IPC_protein[grep(pattern = paste("|",  : 
  replacement has length zero
Error in IPDB_matching_proteome$Sequence[i] <- as.character(isoelectricpointdb$sequence[grep(pattern = paste("|",  : 
  replacement has length zero
Error in IPDB_matching_proteome$MolWeight[i] <- isoelectricpointdb$molecular_weight[grep(pattern = paste("|",  : 
  replacement has length zero
Error in IPDB_matching_proteome$IPC_protein[i] <- isoelectricpointdb$IPC_protein[grep(pattern = paste("|",  : 
  replacement has length zero
Error in IPDB_matching_proteome$Sequence[i] <- as.character(isoelectricpointdb$sequence[grep(pattern = paste("|",  : 
  replacement has length zero
Error in IPDB_matching_proteome$MolWeight[i] <- isoelectricpointdb$molecular_weight[grep(pattern = paste("|",  : 
  replacement has length zero
Error in IPDB_matching_proteome$IPC_protein[i] <- isoelectricpointdb$IPC_protein[grep(pattern = paste("|",  : 
  replacement has length zero
Error in IPDB_matching_proteome$Sequence[i] <- as.character(isoelectricpointdb$sequence[grep(pattern = paste("|",  : 
  replacement has length zero
Error in IPDB_matching_proteome$MolWeight[i] <- isoelectricpointdb$molecular_weight[grep(pattern = paste("|",  : 
  replacement has length zero
Error in IPDB_matching_proteome$IPC_protein[i] <- isoelectricpointdb$IPC_protein[grep(pattern = paste("|",  : 
  replacement has length zero
Error in IPDB_matching_proteome$Sequence[i] <- as.character(isoelectricpointdb$sequence[grep(pattern = paste("|",  : 
  replacement has length zero
Error in IPDB_matching_proteome$MolWeight[i] <- isoelectricpointdb$molecular_weight[grep(pattern = paste("|",  : 
  replacement has length zero
Error in IPDB_matching_proteome$IPC_protein[i] <- isoelectricpointdb$IPC_protein[grep(pattern = paste("|",  : 
  replacement has length zero
Error in IPDB_matching_proteome$Sequence[i] <- as.character(isoelectricpointdb$sequence[grep(pattern = paste("|",  : 
  replacement has length zero
Error in IPDB_matching_proteome$MolWeight[i] <- isoelectricpointdb$molecular_weight[grep(pattern = paste("|",  : 
  replacement has length zero
Error in IPDB_matching_proteome$IPC_protein[i] <- isoelectricpointdb$IPC_protein[grep(pattern = paste("|",  : 
  replacement has length zero
Error in IPDB_matching_proteome$Sequence[i] <- as.character(isoelectricpointdb$sequence[grep(pattern = paste("|",  : 
  replacement has length zero
Error in IPDB_matching_proteome$MolWeight[i] <- isoelectricpointdb$molecular_weight[grep(pattern = paste("|",  : 
  replacement has length zero
Error in IPDB_matching_proteome$IPC_protein[i] <- isoelectricpointdb$IPC_protein[grep(pattern = paste("|",  : 
  replacement has length zero
Error in IPDB_matching_proteome$Sequence[i] <- as.character(isoelectricpointdb$sequence[grep(pattern = paste("|",  : 
  replacement has length zero
Error in IPDB_matching_proteome$MolWeight[i] <- isoelectricpointdb$molecular_weight[grep(pattern = paste("|",  : 
  replacement has length zero
Error in IPDB_matching_proteome$IPC_protein[i] <- isoelectricpointdb$IPC_protein[grep(pattern = paste("|",  : 
  replacement has length zero
Error in IPDB_matching_proteome$Sequence[i] <- as.character(isoelectricpointdb$sequence[grep(pattern = paste("|",  : 
  replacement has length zero
Error in IPDB_matching_proteome$MolWeight[i] <- isoelectricpointdb$molecular_weight[grep(pattern = paste("|",  : 
  replacement has length zero
Error in IPDB_matching_proteome$IPC_protein[i] <- isoelectricpointdb$IPC_protein[grep(pattern = paste("|",  : 
  replacement has length zero
Error in IPDB_matching_proteome$Sequence[i] <- as.character(isoelectricpointdb$sequence[grep(pattern = paste("|",  : 
  replacement has length zero
Error in IPDB_matching_proteome$MolWeight[i] <- isoelectricpointdb$molecular_weight[grep(pattern = paste("|",  : 
  replacement has length zero
Error in IPDB_matching_proteome$IPC_protein[i] <- isoelectricpointdb$IPC_protein[grep(pattern = paste("|",  : 
  replacement has length zero
Error in IPDB_matching_proteome$Sequence[i] <- as.character(isoelectricpointdb$sequence[grep(pattern = paste("|",  : 
  replacement has length zero
Error in IPDB_matching_proteome$MolWeight[i] <- isoelectricpointdb$molecular_weight[grep(pattern = paste("|",  : 
  replacement has length zero
Error in IPDB_matching_proteome$IPC_protein[i] <- isoelectricpointdb$IPC_protein[grep(pattern = paste("|",  : 
  replacement has length zero
Error in IPDB_matching_proteome$Sequence[i] <- as.character(isoelectricpointdb$sequence[grep(pattern = paste("|",  : 
  replacement has length zero
Error in IPDB_matching_proteome$MolWeight[i] <- isoelectricpointdb$molecular_weight[grep(pattern = paste("|",  : 
  replacement has length zero
Error in IPDB_matching_proteome$IPC_protein[i] <- isoelectricpointdb$IPC_protein[grep(pattern = paste("|",  : 
  replacement has length zero
Error in IPDB_matching_proteome$Sequence[i] <- as.character(isoelectricpointdb$sequence[grep(pattern = paste("|",  : 
  replacement has length zero
Error in IPDB_matching_proteome$MolWeight[i] <- isoelectricpointdb$molecular_weight[grep(pattern = paste("|",  : 
  replacement has length zero
Error in IPDB_matching_proteome$IPC_protein[i] <- isoelectricpointdb$IPC_protein[grep(pattern = paste("|",  : 
  replacement has length zero
Error in IPDB_matching_proteome$Sequence[i] <- as.character(isoelectricpointdb$sequence[grep(pattern = paste("|",  : 
  replacement has length zero
Error in IPDB_matching_proteome$MolWeight[i] <- isoelectricpointdb$molecular_weight[grep(pattern = paste("|",  : 
  replacement has length zero
Error in IPDB_matching_proteome$IPC_protein[i] <- isoelectricpointdb$IPC_protein[grep(pattern = paste("|",  : 
  replacement has length zero
```


```
for(i in seq(nrow(IPDB_matching_proteome))){
  try(IPDB_matching_proteome$Hydrophobicity[i] <- aaComp(IPDB_matching_proteome$Sequence[i])[[1]]["NonPolar","Mole%"])
}
```


```
Sequence 1 has unrecognized amino acid types. Output value might be wrong calculatedSequence 1 has unrecognized amino acid types. Output value might be wrong calculatedSequence 1 has unrecognized amino acid types. Output value might be wrong calculatedSequence 1 has unrecognized amino acid types. Output value might be wrong calculatedSequence 1 has unrecognized amino acid types. Output value might be wrong calculatedSequence 1 has unrecognized amino acid types. Output value might be wrong calculatedSequence 1 has unrecognized amino acid types. Output value might be wrong calculatedSequence 1 has unrecognized amino acid types. Output value might be wrong calculatedSequence 1 has unrecognized amino acid types. Output value might be wrong calculatedSequence 1 has unrecognized amino acid types. Output value might be wrong calculatedSequence 1 has unrecognized amino acid types. Output value might be wrong calculatedSequence 1 has unrecognized amino acid types. Output value might be wrong calculatedSequence 1 has unrecognized amino acid types. Output value might be wrong calculatedSequence 1 has unrecognized amino acid types. Output value might be wrong calculatedSequence 1 has unrecognized amino acid types. Output value might be wrong calculatedSequence 1 has unrecognized amino acid types. Output value might be wrong calculatedSequence 1 has unrecognized amino acid types. Output value might be wrong calculatedSequence 1 has unrecognized amino acid types. Output value might be wrong calculatedSequence 1 has unrecognized amino acid types. Output value might be wrong calculatedSequence 1 has unrecognized amino acid types. Output value might be wrong calculatedSequence 1 has unrecognized amino acid types. Output value might be wrong calculatedSequence 1 has unrecognized amino acid types. Output value might be wrong calculatedSequence 1 has unrecognized amino acid types. Output value might be wrong calculatedSequence 1 has unrecognized amino acid types. Output value might be wrong calculatedSequence 1 has unrecognized amino acid types. Output value might be wrong calculatedSequence 1 has unrecognized amino acid types. Output value might be wrong calculatedSequence 1 has unrecognized amino acid types. Output value might be wrong calculatedSequence 1 has unrecognized amino acid types. Output value might be wrong calculatedSequence 1 has unrecognized amino acid types. Output value might be wrong calculatedSequence 1 has unrecognized amino acid types. Output value might be wrong calculatedSequence 1 has unrecognized amino acid types. Output value might be wrong calculatedSequence 1 has unrecognized amino acid types. Output value might be wrong calculatedSequence 1 has unrecognized amino acid types. Output value might be wrong calculatedSequence 1 has unrecognized amino acid types. Output value might be wrong calculatedSequence 1 has unrecognized amino acid types. Output value might be wrong calculatedSequence 1 has unrecognized amino acid types. Output value might be wrong calculatedSequence 1 has unrecognized amino acid types. Output value might be wrong calculatedSequence 1 has unrecognized amino acid types. Output value might be wrong calculatedSequence 1 has unrecognized amino acid types. Output value might be wrong calculatedSequence 1 has unrecognized amino acid types. Output value might be wrong calculatedSequence 1 has unrecognized amino acid types. Output value might be wrong calculatedSequence 1 has unrecognized amino acid types. Output value might be wrong calculatedSequence 1 has unrecognized amino acid types. Output value might be wrong calculatedSequence 1 has unrecognized amino acid types. Output value might be wrong calculatedSequence 1 has unrecognized amino acid types. Output value might be wrong calculatedSequence 1 has unrecognized amino acid types. Output value might be wrong calculatedSequence 1 has unrecognized amino acid types. Output value might be wrong calculatedSequence 1 has unrecognized amino acid types. Output value might be wrong calculatedSequence 1 has unrecognized amino acid types. Output value might be wrong calculatedSequence 1 has unrecognized amino acid types. Output value might be wrong calculatedSequence 1 has unrecognized amino acid types. Output value might be wrong calculatedSequence 1 has unrecognized amino acid types. Output value might be wrong calculatedSequence 1 has unrecognized amino acid types. Output value might be wrong calculatedSequence 1 has unrecognized amino acid types. Output value might be wrong calculatedSequence 1 has unrecognized amino acid types. Output value might be wrong calculatedSequence 1 has unrecognized amino acid types. Output value might be wrong calculatedSequence 1 has unrecognized amino acid types. Output value might be wrong calculatedSequence 1 has unrecognized amino acid types. Output value might be wrong calculatedSequence 1 has unrecognized amino acid types. Output value might be wrong calculatedSequence 1 has unrecognized amino acid types. Output value might be wrong calculatedSequence 1 has unrecognized amino acid types. Output value might be wrong calculatedSequence 1 has unrecognized amino acid types. Output value might be wrong calculatedSequence 1 has unrecognized amino acid types. Output value might be wrong calculatedSequence 1 has unrecognized amino acid types. Output value might be wrong calculatedSequence 1 has unrecognized amino acid types. Output value might be wrong calculatedSequence 1 has unrecognized amino acid types. Output value might be wrong calculatedSequence 1 has unrecognized amino acid types. Output value might be wrong calculatedSequence 1 has unrecognized amino acid types. Output value might be wrong calculatedSequence 1 has unrecognized amino acid types. Output value might be wrong calculatedSequence 1 has unrecognized amino acid types. Output value might be wrong calculatedSequence 1 has unrecognized amino acid types. Output value might be wrong calculatedSequence 1 has unrecognized amino acid types. Output value might be wrong calculatedSequence 1 has unrecognized amino acid types. Output value might be wrong calculatedSequence 1 has unrecognized amino acid types. Output value might be wrong calculatedSequence 1 has unrecognized amino acid types. Output value might be wrong calculatedSequence 1 has unrecognized amino acid types. Output value might be wrong calculatedSequence 1 has unrecognized amino acid types. Output value might be wrong calculatedSequence 1 has unrecognized amino acid types. Output value might be wrong calculatedSequence 1 has unrecognized amino acid types. Output value might be wrong calculatedSequence 1 has unrecognized amino acid types. Output value might be wrong calculatedSequence 1 has unrecognized amino acid types. Output value might be wrong calculatedSequence 1 has unrecognized amino acid types. Output value might be wrong calculatedSequence 1 has unrecognized amino acid types. Output value might be wrong calculatedSequence 1 has unrecognized amino acid types. Output value might be wrong calculatedSequence 1 has unrecognized amino acid types. Output value might be wrong calculatedSequence 1 has unrecognized amino acid types. Output value might be wrong calculatedSequence 1 has unrecognized amino acid types. Output value might be wrong calculatedSequence 1 has unrecognized amino acid types. Output value might be wrong calculatedSequence 1 has unrecognized amino acid types. Output value might be wrong calculatedSequence 1 has unrecognized amino acid types. Output value might be wrong calculatedSequence 1 has unrecognized amino acid types. Output value might be wrong calculatedSequence 1 has unrecognized amino acid types. Output value might be wrong calculatedSequence 1 has unrecognized amino acid types. Output value might be wrong calculatedSequence 1 has unrecognized amino acid types. Output value might be wrong calculatedSequence 1 has unrecognized amino acid types. Output value might be wrong calculatedSequence 1 has unrecognized amino acid types. Output value might be wrong calculatedSequence 1 has unrecognized amino acid types. Output value might be wrong calculatedSequence 1 has unrecognized amino acid types. Output value might be wrong calculatedSequence 1 has unrecognized amino acid types. Output value might be wrong calculatedSequence 1 has unrecognized amino acid types. Output value might be wrong calculatedSequence 1 has unrecognized amino acid types. Output value might be wrong calculatedSequence 1 has unrecognized amino acid types. Output value might be wrong calculatedSequence 1 has unrecognized amino acid types. Output value might be wrong calculatedSequence 1 has unrecognized amino acid types. Output value might be wrong calculatedSequence 1 has unrecognized amino acid types. Output value might be wrong calculatedSequence 1 has unrecognized amino acid types. Output value might be wrong calculatedSequence 1 has unrecognized amino acid types. Output value might be wrong calculatedSequence 1 has unrecognized amino acid types. Output value might be wrong calculatedSequence 1 has unrecognized amino acid types. Output value might be wrong calculatedSequence 1 has unrecognized amino acid types. Output value might be wrong calculatedSequence 1 has unrecognized amino acid types. Output value might be wrong calculatedSequence 1 has unrecognized amino acid types. Output value might be wrong calculatedSequence 1 has unrecognized amino acid types. Output value might be wrong calculatedSequence 1 has unrecognized amino acid types. Output value might be wrong calculatedSequence 1 has unrecognized amino acid types. Output value might be wrong calculatedSequence 1 has unrecognized amino acid types. Output value might be wrong calculatedSequence 1 has unrecognized amino acid types. Output value might be wrong calculatedSequence 1 has unrecognized amino acid types. Output value might be wrong calculatedSequence 1 has unrecognized amino acid types. Output value might be wrong calculatedSequence 1 has unrecognized amino acid types. Output value might be wrong calculatedSequence 1 has unrecognized amino acid types. Output value might be wrong calculatedSequence 1 has unrecognized amino acid types. Output value might be wrong calculatedSequence 1 has unrecognized amino acid types. Output value might be wrong calculatedSequence 1 has unrecognized amino acid types. Output value might be wrong calculatedSequence 1 has unrecognized amino acid types. Output value might be wrong calculatedSequence 1 has unrecognized amino acid types. Output value might be wrong calculatedSequence 1 has unrecognized amino acid types. Output value might be wrong calculatedSequence 1 has unrecognized amino acid types. Output value might be wrong calculatedSequence 1 has unrecognized amino acid types. Output value might be wrong calculatedSequence 1 has unrecognized amino acid types. Output value might be wrong calculatedSequence 1 has unrecognized amino acid types. Output value might be wrong calculatedSequence 1 has unrecognized amino acid types. Output value might be wrong calculatedSequence 1 has unrecognized amino acid types. Output value might be wrong calculatedSequence 1 has unrecognized amino acid types. Output value might be wrong calculatedSequence 1 has unrecognized amino acid types. Output value might be wrong calculatedSequence 1 has unrecognized amino acid types. Output value might be wrong calculatedSequence 1 has unrecognized amino acid types. Output value might be wrong calculatedSequence 1 has unrecognized amino acid types. Output value might be wrong calculatedSequence 1 has unrecognized amino acid types. Output value might be wrong calculatedSequence 1 has unrecognized amino acid types. Output value might be wrong calculatedSequence 1 has unrecognized amino acid types. Output value might be wrong calculatedSequence 1 has unrecognized amino acid types. Output value might be wrong calculatedSequence 1 has unrecognized amino acid types. Output value might be wrong calculatedSequence 1 has unrecognized amino acid types. Output value might be wrong calculatedSequence 1 has unrecognized amino acid types. Output value might be wrong calculatedSequence 1 has unrecognized amino acid types. Output value might be wrong calculatedSequence 1 has unrecognized amino acid types. Output value might be wrong calculatedSequence 1 has unrecognized amino acid types. Output value might be wrong calculatedSequence 1 has unrecognized amino acid types. Output value might be wrong calculatedSequence 1 has unrecognized amino acid types. Output value might be wrong calculatedSequence 1 has unrecognized amino acid types. Output value might be wrong calculatedSequence 1 has unrecognized amino acid types. Output value might be wrong calculatedSequence 1 has unrecognized amino acid types. Output value might be wrong calculatedSequence 1 has unrecognized amino acid types. Output value might be wrong calculatedSequence 1 has unrecognized amino acid types. Output value might be wrong calculatedSequence 1 has unrecognized amino acid types. Output value might be wrong calculatedSequence 1 has unrecognized amino acid types. Output value might be wrong calculatedSequence 1 has unrecognized amino acid types. Output value might be wrong calculatedSequence 1 has unrecognized amino acid types. Output value might be wrong calculatedSequence 1 has unrecognized amino acid types. Output value might be wrong calculatedSequence 1 has unrecognized amino acid types. Output value might be wrong calculatedSequence 1 has unrecognized amino acid types. Output value might be wrong calculatedSequence 1 has unrecognized amino acid types. Output value might be wrong calculatedSequence 1 has unrecognized amino acid types. Output value might be wrong calculatedSequence 1 has unrecognized amino acid types. Output value might be wrong calculatedSequence 1 has unrecognized amino acid types. Output value might be wrong calculatedSequence 1 has unrecognized amino acid types. Output value might be wrong calculatedSequence 1 has unrecognized amino acid types. Output value might be wrong calculatedSequence 1 has unrecognized amino acid types. Output value might be wrong calculatedSequence 1 has unrecognized amino acid types. Output value might be wrong calculatedSequence 1 has unrecognized amino acid types. Output value might be wrong calculatedSequence 1 has unrecognized amino acid types. Output value might be wrong calculatedSequence 1 has unrecognized amino acid types. Output value might be wrong calculatedSequence 1 has unrecognized amino acid types. Output value might be wrong calculatedSequence 1 has unrecognized amino acid types. Output value might be wrong calculatedSequence 1 has unrecognized amino acid types. Output value might be wrong calculatedSequence 1 has unrecognized amino acid types. Output value might be wrong calculatedSequence 1 has unrecognized amino acid types. Output value might be wrong calculatedSequence 1 has unrecognized amino acid types. Output value might be wrong calculatedSequence 1 has unrecognized amino acid types. Output value might be wrong calculatedSequence 1 has unrecognized amino acid types. Output value might be wrong calculatedSequence 1 has unrecognized amino acid types. Output value might be wrong calculatedSequence 1 has unrecognized amino acid types. Output value might be wrong calculatedSequence 1 has unrecognized amino acid types. Output value might be wrong calculatedSequence 1 has unrecognized amino acid types. Output value might be wrong calculatedSequence 1 has unrecognized amino acid types. Output value might be wrong calculatedSequence 1 has unrecognized amino acid types. Output value might be wrong calculatedSequence 1 has unrecognized amino acid types. Output value might be wrong calculatedSequence 1 has unrecognized amino acid types. Output value might be wrong calculatedSequence 1 has unrecognized amino acid types. Output value might be wrong calculatedSequence 1 has unrecognized amino acid types. Output value might be wrong calculatedSequence 1 has unrecognized amino acid types. Output value might be wrong calculatedSequence 1 has unrecognized amino acid types. Output value might be wrong calculatedSequence 1 has unrecognized amino acid types. Output value might be wrong calculatedSequence 1 has unrecognized amino acid types. Output value might be wrong calculatedSequence 1 has unrecognized amino acid types. Output value might be wrong calculatedSequence 1 has unrecognized amino acid types. Output value might be wrong calculatedSequence 1 has unrecognized amino acid types. Output value might be wrong calculatedSequence 1 has unrecognized amino acid types. Output value might be wrong calculatedSequence 1 has unrecognized amino acid types. Output value might be wrong calculatedSequence 1 has unrecognized amino acid types. Output value might be wrong calculatedSequence 1 has unrecognized amino acid types. Output value might be wrong calculatedSequence 1 has unrecognized amino acid types. Output value might be wrong calculatedSequence 1 has unrecognized amino acid types. Output value might be wrong calculatedSequence 1 has unrecognized amino acid types. Output value might be wrong calculatedSequence 1 has unrecognized amino acid types. Output value might be wrong calculatedSequence 1 has unrecognized amino acid types. Output value might be wrong calculatedSequence 1 has unrecognized amino acid types. Output value might be wrong calculatedSequence 1 has unrecognized amino acid types. Output value might be wrong calculatedSequence 1 has unrecognized amino acid types. Output value might be wrong calculatedSequence 1 has unrecognized amino acid types. Output value might be wrong calculatedSequence 1 has unrecognized amino acid types. Output value might be wrong calculatedSequence 1 has unrecognized amino acid types. Output value might be wrong calculatedSequence 1 has unrecognized amino acid types. Output value might be wrong calculatedSequence 1 has unrecognized amino acid types. Output value might be wrong calculatedSequence 1 has unrecognized amino acid types. Output value might be wrong calculated
```


Some of the accession numbers were not included in the isoelectricpointdb.


```
sum(sapply(IPDB_matching_proteome$Sequence, function(x)is.na(x)))
```


```
[1] 173
```


```
sum(sapply(IPDB_matching_proteome$MolWeight, function(x)is.na(x)))
```


```
[1] 173
```


```
sum(sapply(IPDB_matching_proteome$IPC_protein, function(x)is.na(x)))
```


```
[1] 173
```


```
sum(sapply(IPDB_matching_proteome$Hydrophobicity, function(x)is.nan(x)))
```


```
[1] 173
```


### 6.1.1 Plots

#### 6.1.1.1 Molecular Weight


```
mw_distribution <- data.frame(
  MolWeight = c(IPDB_matching$MolWeight, IPDB_matching_proteome$MolWeight),
  set = c(rep("PTex", length(IPDB_matching$MolWeight)), rep("Proteome", length(IPDB_matching_proteome$MolWeight)))
)
ggplot(mw_distribution, aes(x = set, y = MolWeight)) +
  geom_boxplot() + 
  scale_y_log10() +
  xlab("") +
  ylab("Molecular Weight")
pdf("MolWeight_boxplot.pdf", width = 10, height = 7)
```


```
last_plot()
dev.off()
```


```
png 
  2
```

#### 6.1.1.2 Isoelectric Point

##### 6.1.1.2.1 Boxplot


```
pi_distribution <- data.frame(
  IPC_protein = c(IPDB_matching$IPC_protein, IPDB_matching_proteome$IPC_protein),
  set = c(rep("PTex", length(IPDB_matching$IPC_protein)), rep("Proteome", length(IPDB_matching_proteome$IPC_protein)))
)
ggplot(pi_distribution, aes(x = set, y = IPC_protein)) +
  geom_boxplot() + 
  xlab("") +
  ylab("IPC_protein")
pdf("IPC_protein_boxplot.pdf", width = 10, height = 7)
```


```
last_plot()
dev.off()
```


```
png 
  2
```

##### 6.1.1.2.2 Density Plot

A density plot of the IPC\_protein distributions comparing PTex, Landthaler, Rick unique, Rick high and proteome is prepared.


```
rick_high_ip <- data.frame(
  IPC_protein = IPDB_matching_proteome$IPC_protein[IPDB_matching_proteome$UniProt %in% rick_high$Protein_ID],
  set = rep("Rick.high", nrow(rick_high))
)
rick_unique_ip <- data.frame(
  IPC_protein = IPDB_matching_proteome$IPC_protein[IPDB_matching_proteome$UniProt %in% rick_unique$Protein_ID],
  set = rep("Rick.unique", nrow(rick_unique))
)
# 6 accessions of the Landthaler data are not included in the isoelectricpointdb and will be removed
landthaler_ip <- data.frame(
  IPC_protein = IPDB_matching_proteome$IPC_protein[IPDB_matching_proteome$UniProt %in% hekmRBP$Uniprot.entry[-which(hekmRBP$Uniprot.entry %in% IPDB_matching_proteome$UniProt==FALSE)]],
  set = rep("Landthaler.mRBP", length(which(hekmRBP$Uniprot.entry %in% IPDB_matching_proteome$UniProt==TRUE)))
)
ip_density_distribution <- data.frame(
  IPC_protein = c(IPDB_matching$IPC_protein, landthaler_ip$IPC_protein, rick_unique_ip$IPC_protein, rick_high_ip$IPC_protein, IPDB_matching_proteome$IPC_protein),
  set = c(rep("PTex", nrow(IPDB_matching)), as.character(landthaler_ip$set), as.character(rick_unique_ip$set), as.character(rick_high_ip$set), rep("Proteome", nrow(IPDB_matching_proteome)))
)
ggplot2::ggplot(ip_density_distribution, aes(x = IPC_protein, colour = set)) +
  geom_density()
pdf("IPC_protein_density.pdf", width = 10, height = 7)
```


```
last_plot()
dev.off()
```


```
png 
  2
```

#### 6.1.1.3 Hydrophobicity


```
hydrophobicity_distribution <- data.frame(
  Hydrophobicity = c(IPDB_matching$Hydrophobicity, IPDB_matching_proteome$Hydrophobicity),
  set = c(rep("PTex", length(IPDB_matching$Hydrophobicity)), rep("Proteome", length(IPDB_matching_proteome$Hydrophobicity)))
)
ggplot(hydrophobicity_distribution, aes(x = set, y = Hydrophobicity)) +
  geom_boxplot() + 
  xlab("") +
  ylab("Hydrophobicity")
pdf("Hydrophobicity_boxplot.pdf", width = 10, height = 7)
```


```
last_plot()
dev.off()
```


```
png 
  2
```

## 6.2 RBD

The RBD domain annotation is performed using DAVID online tool. Since it only accepts lists with maximum 3000 entries 10 randomly sampled lists were created.


```
set.seed(42)
accessions_S1 <- sample(accessions, 3000)
accessions_S2 <- sample(accessions, 3000)
accessions_S3 <- sample(accessions, 3000)
accessions_S4 <- sample(accessions, 3000)
accessions_S5 <- sample(accessions, 3000)
accessions_S6 <- sample(accessions, 3000)
accessions_S7 <- sample(accessions, 3000)
accessions_S8 <- sample(accessions, 3000)
accessions_S9 <- sample(accessions, 3000)
accessions_S10 <- sample(accessions, 3000)
write.table(accessions_S1, "accessions_S1.tsv", row.names = F, col.names = F)
write.table(accessions_S2, "accessions_S2.tsv", row.names = F, col.names = F)
write.table(accessions_S3, "accessions_S3.tsv", row.names = F, col.names = F)
write.table(accessions_S4, "accessions_S4.tsv", row.names = F, col.names = F)
write.table(accessions_S5, "accessions_S5.tsv", row.names = F, col.names = F)
write.table(accessions_S6, "accessions_S6.tsv", row.names = F, col.names = F)
write.table(accessions_S7, "accessions_S7.tsv", row.names = F, col.names = F)
write.table(accessions_S8, "accessions_S8.tsv", row.names = F, col.names = F)
write.table(accessions_S9, "accessions_S9.tsv", row.names = F, col.names = F)
write.table(accessions_S10, "accessions_S10.tsv", row.names = F, col.names = F)
```

## 6.3 GO

GO term analyses was performed by the PANTHER online tool using the `accessions` table.

# 7 Master Table

A master table containing identifiers, enrichments, matches and annotations is created.


```
# create vector holding rows of sig_norm_MassSpecData that are in matching_table
MSD_to_MT <- which(sapply(X = sig_norm_MassSpecData$Majority.protein.IDs, FUN = function(x)unlist(strsplit(as.character(x), split=";"))) %in% matching_table$Majority.protein.IDs)
master_table <- data.frame(
  UniProt = accessions,
  Majority.protein.IDs = sig_norm_MassSpecData$Majority.protein.IDs[MSD_to_MT],
  MPID.reviewed = as.character(matching_table$MPIDr),
  Protein.name = sig_norm_MassSpecData$Protein.names[MSD_to_MT],
  Gene.name = matching_table$gene_name,
  FC.PTex_0015.mean = sig_norm_MassSpecData$FC.PTex_0015.mean[MSD_to_MT],
  padj.PTex_0015 = sig_norm_MassSpecData$padj.PTex_0015[MSD_to_MT],
  FC.PTex_015.mean = sig_norm_MassSpecData$FC.PTex_015.mean[MSD_to_MT],
  padj.PTex_015 = sig_norm_MassSpecData$padj.PTex_015[MSD_to_MT],
  FC.PTex_15.mean = sig_norm_MassSpecData$FC.PTex_15.mean[MSD_to_MT],
  padj.PTex_15 = sig_norm_MassSpecData$padj.PTex_15[MSD_to_MT],
  Gerstberger.RBP = matching_table$Gerstberger.RBP,
  Gerstberger.TF = matching_table$Gerstberger.TF,
  Landthaler.mRBP = matching_table$Landthaler.mRBP,
  Preiss = matching_table$Preiss,
  Brannan.annotated = matching_table$Brannan.annotated,
  Brannan.predicted = matching_table$Brannan.predicted,
  Hubstenberger.PBP = matching_table$Hubstenberger.PBP,
  Rick.high = matching_table$Rick.high,
  Rick.low = matching_table$Rick.low,
  Rick.unique = matching_table$Rick.unique,
  Caric = matching_table$Caric,
  IPC_protein = IPDB_matching$IPC_protein,
  MolWeight = IPDB_matching$MolWeight,
  Hydrophobicity = IPDB_matching$Hydrophobicity
)
write.table(master_table, "master_table.tsv", sep = "\t", row.names = F)
```

# 8 Session Info


```
sessionInfo()
```


```
R version 3.5.1 (2018-07-02)
Platform: x86_64-pc-linux-gnu (64-bit)
Running under: Arch Linux

Matrix products: default
BLAS/LAPACK: /usr/lib/libopenblas_haswellp-r0.3.3.so

locale:
 [1] LC_CTYPE=en_US.UTF-8       LC_NUMERIC=C               LC_TIME=en_US.UTF-8        LC_COLLATE=en_US.UTF-8     LC_MONETARY=en_US.UTF-8   
 [6] LC_MESSAGES=en_US.UTF-8    LC_PAPER=en_US.UTF-8       LC_NAME=C                  LC_ADDRESS=C               LC_TELEPHONE=C            
[11] LC_MEASUREMENT=en_US.UTF-8 LC_IDENTIFICATION=C       

attached base packages:
[1] stats     graphics  grDevices utils     datasets  methods   base     

other attached packages:
[1] DT_0.4         Peptides_2.4   GGally_1.4.0   ggplot2_3.0.0  reshape2_1.4.3 limma_3.36.3   dplyr_0.7.6   

loaded via a namespace (and not attached):
 [1] Rcpp_1.0.0         later_0.7.4        RColorBrewer_1.1-2 pillar_1.3.0       compiler_3.5.1     plyr_1.8.4         bindr_0.1.1        base64enc_0.1-3   
 [9] tools_3.5.1        digest_0.6.17      jsonlite_1.5       evaluate_0.11      tibble_1.4.2       gtable_0.2.0       pkgconfig_2.0.2    rlang_0.2.2       
[17] shiny_1.1.0        rstudioapi_0.7     crosstalk_1.0.0    yaml_2.2.0         bindrcpp_0.2.2     withr_2.1.2        stringr_1.3.1      knitr_1.20        
[25] htmlwidgets_1.2    rprojroot_1.3-2    grid_3.5.1         tidyselect_0.2.4   reshape_0.8.7      glue_1.3.0         R6_2.3.0           rmarkdown_1.10    
[33] purrr_0.2.5        magrittr_1.5       promises_1.0.1     backports_1.1.2    scales_1.0.0       htmltools_0.3.6    assertthat_0.2.0   xtable_1.8-3      
[41] mime_0.5           colorspace_1.3-2   httpuv_1.4.5       labeling_0.3       stringi_1.2.4      lazyeval_0.2.1     munsell_0.5.0      crayon_1.3.4
```

LS0tCnRpdGxlOiAiUFRleCBIRUsyOTMgTVMgQW5hbHlzaXMiCm91dHB1dDoKICBodG1sX25vdGVib29rOiAKICAgIHRvYzogeWVzCiAgICBudW1iZXJfc2VjdGlvbnM6IHRydWUKICAgIHNtYXJ0OiBmYWxzZQogICAgbWRfZXh0ZW5zaW9uczogK3NtYXJ0Ci0tLQoKIyBJbnRyb2R1Y3Rpb24KClRoaXMgZG9jdW1lbnQgZ3VpZGVzIHRocm91Z2ggdGhlIGFuYWx5c2lzIG9mIHRoZSBNUyBkYXRhIGZvciB0aGUgUFRleCBIRUsyOTMgcHJvamVjdC4KSW5wdXRkYXRhIGhhdmUgdG8gYmUgcHJvdmlkZWQgaW4gdGhlIGZvbGRlciBgaW5wdXRkYXRhYCBpbnNpZGUgdGhlIHdvcmtpbmcgZGlyZWN0b3J5LiBUaGVzZSBhcmU6CgogICAgd29ya2luZyBkaXJlY3RvcnkvCiAgICAgIFBUZXguUm1kCiAgICAgIGlucHV0ZGF0YS8KICAgICAgICBwcm90ZWluR3JvdXBzLnR4dCAgICAgICAgICAgICAgICAgICAjIE1TIGRhdGEKICAgICAgICBSQlAgdGFibGUtVGFibGUgMS5jc3YgICAgICAgICAgICAgICAjIEdlcnN0YmVyZ2VyIHJldmlldyAoMjAxNCkgUkJQcwogICAgICAgIGh1bWFuIFRGcy1UYWJsZSAxLmNzdiAgICAgICAgICAgICAgICMgR2Vyc3RiZXJnZXIgcmV2aWV3ICgyMDE0KSBURnMKICAgICAgICAyMDE1LTA2LTA5X2h1bWFuX2ludGVyYWN0b21lcy5jc3YgICAjIExhbmR0aGFsZXIgaHVtYW4gbVJOQSBJQwogICAgICAgIHByZWlzc19nZW5lbmFtZXMuY3N2ICAgICAgICAgICAgICAgICMgUHJlaXNzCiAgICAgICAgQnJhbm5hbl9hbm5vdGF0ZWQudHh0ICAgICAgICAgICAgICAgIyBCcmFubmFuIFNPTkFSIGFubm90YXRlZCBSQlBzCiAgICAgICAgQnJhbm5hbl9TT05BUl8wLjc5LnR4dCAgICAgICAgICAgICAgIyBCcmFubmFuIFNPTkFSIHByZWRpY3RlZAogICAgICAgIGh1YnN0ZW5iZXJnZXIuY3N2ICAgICAgICAgICAgICAgICAgICMgSHVic3RlbmJlcmdlciBQLUJvZHkgUHJvdGVpbnMKICAgICAgICBSSUNLX2hpZ2hfY29uZi5jc3YgICAgICAgICAgICAgICAgICAjIFJJQ0sgaGlnaCBjb25maWRlbmNlCiAgICAgICAgUklDS19sb3dfY29uZi5jc3YgICAgICAgICAgICAgICAgICAgIyBSSUNLIGxvdyBjb25maWRlbmNlCiAgICAgICAgUklDS191bmlxdWUuY3N2ICAgICAgICAgICAgICAgICAgICAgIyBSSUNLIHVuaXF1ZQogICAgICAgIGNhcmljLmNzdiAgICAgICAgICAgICAgICAgICAgICAgICAgICMgQ0FSSUMKICAgICAgICBodW1hbl9wcm90ZW9tZV9yZXZpZXdlZF8yOTAxMTgubGlzdCAjIFVuaVByb3QgU3dpc3NQcm90CiAgICAgICAgVVAwMDAwMDU2NDBfOTYwNl9hbGwuZmFzdGEucEkuY3N2ICAgIyBJc29lbGVjdHJpY3BvaW50ZGIKCiMgTGlicmFyaWVzIAoKVGhlIGZvbGxvd2luZyBsaWJyYXJpZXMgYXJlIG5lZWRlZCBmb3IgdGhlIGFuYWx5c2lzLiBQYWNrYWdlcyBoYXZlIHRvIGJlIGluc3RhbGxlZCBwcmlvciB0byBsb2FkaW5nLgoKYGBge3J9CmxpYnJhcnkoImRwbHlyIikKbGlicmFyeSgibGltbWEiKQpsaWJyYXJ5KCJyZXNoYXBlMiIpCmxpYnJhcnkoImdncGxvdDIiKQpsaWJyYXJ5KCJHR2FsbHkiKQpsaWJyYXJ5KCJQZXB0aWRlcyIpCmxpYnJhcnkoIkRUIikKYGBgCgpUbyBwcm9kdWNlIHBhaXJ3aXNlIHNjYXR0ZXIgcGxvdHMgd2l0aCBjb3JyZWxhdGlvbiBpbmZvcm1hdGlvbiB3ZSB1c2UgdGhlIGBnZ3BhaXJzKClgIGZ1bmN0aW9uIGZyb20gdGhlIGBHR2FsbHlgIHBhY2thZ2UuCkN1c3RvbSB0aGVtZSBmb3IgcGFpcndpc2Ugc2NhdHRlciBwbG90czoKCmBgYHtyfQpzY2F0dGVyX3RoZW1lIDwtIHRoZW1lKGxlZ2VuZC5wb3NpdGlvbiA9ICJub25lIiwgCiAgICAgIHBhbmVsLmdyaWQubWFqb3IgPSBlbGVtZW50X2JsYW5rKCksIAogICAgICBheGlzLnRpY2tzID0gZWxlbWVudF9ibGFuaygpLCAKICAgICAgcGFuZWwuYm9yZGVyID0gZWxlbWVudF9yZWN0KGxpbmV0eXBlID0gInNvbGlkIiwgY29sb3VyID0gImJsYWNrIiwgZmlsbCA9IE5BKSkKCmdncGxvdCA8LSBmdW5jdGlvbiguLi4pIGdncGxvdDI6OmdncGxvdCguLi4pICsgc2NhbGVfY29sb3VyX21hbnVhbCh2YWx1ZXMgPSBjKCJibGFjayIsInJlZCIpKSArIHNjYWxlX2ZpbGxfbWFudWFsKHZhbHVlcyA9IGMoImJsYWNrIiwicmVkIikpCnVubG9ja0JpbmRpbmcoImdncGxvdCIscGFyZW50LmVudihhc05hbWVzcGFjZSgiR0dhbGx5IikpKQphc3NpZ24oImdncGxvdCIsZ2dwbG90LHBhcmVudC5lbnYoYXNOYW1lc3BhY2UoIkdHYWxseSIpKSkKYGBgCgojIERhdGEgcHJlcGFyYXRpb24KClRoZSBwcmltYXJ5IE1TIGRhdGEgYXJlIGNvbnRhaW5lZCBpbiB0aGUgZmlsZSBgcHJvdGVpbkdyb3Vwcy50eHRgLgoKYGBge3IgbG9hZCBwcmltYXJ5IE1TIGRhdGF9CnByaW1hcnlNYXNzU3BlY0RhdGEgPC0gcmVhZC50YWJsZSgiaW5wdXRkYXRhL3Byb3RlaW5Hcm91cHMudHh0IiwgaGVhZGVyID0gVFJVRSwgc2VwID0gIlx0IikKYGBgCgpUbyBlYXNlIGxhdGVyIGFuYWx5c2VzLCBjaGFyYWN0ZXIgdmVjdG9ycyBvZiBjb2x1bW5uYW1lcyBhcmUgY3JlYXRlZC4KCmBgYHtyIGNvbG5hbWVzIGludGVuc2l0aWVzfQppbnRlbnNpdGllcyA8LSBjKCJJbnRlbnNpdHkuSW5wdXRfTm9DbF9JIiwKICAgICAgICAgICAgICAgIkludGVuc2l0eS5JbnB1dF9Ob0NsX0lJIiwKICAgICAgICAgICAgICAgIkludGVuc2l0eS5JbnB1dF9Ob0NsX0lJSSIsCiAgICAgICAgICAgICAgICJJbnRlbnNpdHkuSW5wdXRfMDAxNV9JIiwKICAgICAgICAgICAgICAgIkludGVuc2l0eS5JbnB1dF8wMDE1X0lJIiwKICAgICAgICAgICAgICAgIkludGVuc2l0eS5JbnB1dF8wMDE1X0lJSSIsCiAgICAgICAgICAgICAgICJJbnRlbnNpdHkuSW5wdXRfMDE1X0kiLAogICAgICAgICAgICAgICAiSW50ZW5zaXR5LklucHV0XzAxNV9JSSIsCiAgICAgICAgICAgICAgICJJbnRlbnNpdHkuSW5wdXRfMDE1X0lJSSIsCiAgICAgICAgICAgICAgICJJbnRlbnNpdHkuSW5wdXRfMTVfSSIsCiAgICAgICAgICAgICAgICJJbnRlbnNpdHkuSW5wdXRfMTVfSUkiLAogICAgICAgICAgICAgICAiSW50ZW5zaXR5LklucHV0XzE1X0lJSSIsCiAgICAgICAgICAgICAgICJJbnRlbnNpdHkuUFRleF9Ob0NsX0kiLAogICAgICAgICAgICAgICAiSW50ZW5zaXR5LlBUZXhfTm9DbF9JSSIsCiAgICAgICAgICAgICAgICJJbnRlbnNpdHkuUFRleF9Ob0NsX0lJSSIsCiAgICAgICAgICAgICAgICJJbnRlbnNpdHkuUFRleF8wMDE1X0kiLAogICAgICAgICAgICAgICAiSW50ZW5zaXR5LlBUZXhfMDAxNV9JSSIsCiAgICAgICAgICAgICAgICJJbnRlbnNpdHkuUFRleF8wMDE1X0lJSSIsCiAgICAgICAgICAgICAgICJJbnRlbnNpdHkuUFRleF8wMTVfSSIsCiAgICAgICAgICAgICAgICJJbnRlbnNpdHkuUFRleF8wMTVfSUkiLAogICAgICAgICAgICAgICAiSW50ZW5zaXR5LlBUZXhfMDE1X0lJSSIsCiAgICAgICAgICAgICAgICJJbnRlbnNpdHkuUFRleF8xNV9JIiwKICAgICAgICAgICAgICAgIkludGVuc2l0eS5QVGV4XzE1X0lJIiwKICAgICAgICAgICAgICAgIkludGVuc2l0eS5QVGV4XzE1X0lJSSIpCmBgYAoKYGBge3IgY29sbmFtZXMgTEZRfQpMRlEgPC0gYygiTEZRLmludGVuc2l0eS5JbnB1dF9Ob0NsX0kiLAogICAgICAgICAgICAgICAiTEZRLmludGVuc2l0eS5JbnB1dF9Ob0NsX0lJIiwKICAgICAgICAgICAgICAgIkxGUS5pbnRlbnNpdHkuSW5wdXRfTm9DbF9JSUkiLAogICAgICAgICAgICAgICAiTEZRLmludGVuc2l0eS5JbnB1dF8wMDE1X0kiLAogICAgICAgICAgICAgICAiTEZRLmludGVuc2l0eS5JbnB1dF8wMDE1X0lJIiwKICAgICAgICAgICAgICAgIkxGUS5pbnRlbnNpdHkuSW5wdXRfMDAxNV9JSUkiLAogICAgICAgICAgICAgICAiTEZRLmludGVuc2l0eS5JbnB1dF8wMTVfSSIsCiAgICAgICAgICAgICAgICJMRlEuaW50ZW5zaXR5LklucHV0XzAxNV9JSSIsCiAgICAgICAgICAgICAgICJMRlEuaW50ZW5zaXR5LklucHV0XzAxNV9JSUkiLAogICAgICAgICAgICAgICAiTEZRLmludGVuc2l0eS5JbnB1dF8xNV9JIiwKICAgICAgICAgICAgICAgIkxGUS5pbnRlbnNpdHkuSW5wdXRfMTVfSUkiLAogICAgICAgICAgICAgICAiTEZRLmludGVuc2l0eS5JbnB1dF8xNV9JSUkiLAogICAgICAgICAgICAgICAiTEZRLmludGVuc2l0eS5QVGV4X05vQ2xfSSIsCiAgICAgICAgICAgICAgICJMRlEuaW50ZW5zaXR5LlBUZXhfTm9DbF9JSSIsCiAgICAgICAgICAgICAgICJMRlEuaW50ZW5zaXR5LlBUZXhfTm9DbF9JSUkiLAogICAgICAgICAgICAgICAiTEZRLmludGVuc2l0eS5QVGV4XzAwMTVfSSIsCiAgICAgICAgICAgICAgICJMRlEuaW50ZW5zaXR5LlBUZXhfMDAxNV9JSSIsCiAgICAgICAgICAgICAgICJMRlEuaW50ZW5zaXR5LlBUZXhfMDAxNV9JSUkiLAogICAgICAgICAgICAgICAiTEZRLmludGVuc2l0eS5QVGV4XzAxNV9JIiwKICAgICAgICAgICAgICAgIkxGUS5pbnRlbnNpdHkuUFRleF8wMTVfSUkiLAogICAgICAgICAgICAgICAiTEZRLmludGVuc2l0eS5QVGV4XzAxNV9JSUkiLAogICAgICAgICAgICAgICAiTEZRLmludGVuc2l0eS5QVGV4XzE1X0kiLAogICAgICAgICAgICAgICAiTEZRLmludGVuc2l0eS5QVGV4XzE1X0lJIiwKICAgICAgICAgICAgICAgIkxGUS5pbnRlbnNpdHkuUFRleF8xNV9JSUkiKQpgYGAKCmBgYHtyIGNvbG5hbWVzIGlCQVF9CklCQVEgPC0gYygiaUJBUS5JbnB1dF9Ob0NsX0kiLAogICAgICAgICAgICAgICAiaUJBUS5JbnB1dF9Ob0NsX0lJIiwKICAgICAgICAgICAgICAgImlCQVEuSW5wdXRfTm9DbF9JSUkiLAogICAgICAgICAgICAgICAiaUJBUS5JbnB1dF8wMDE1X0kiLAogICAgICAgICAgICAgICAiaUJBUS5JbnB1dF8wMDE1X0lJIiwKICAgICAgICAgICAgICAgImlCQVEuSW5wdXRfMDAxNV9JSUkiLAogICAgICAgICAgICAgICAiaUJBUS5JbnB1dF8wMTVfSSIsCiAgICAgICAgICAgICAgICJpQkFRLklucHV0XzAxNV9JSSIsCiAgICAgICAgICAgICAgICJpQkFRLklucHV0XzAxNV9JSUkiLAogICAgICAgICAgICAgICAiaUJBUS5JbnB1dF8xNV9JIiwKICAgICAgICAgICAgICAgImlCQVEuSW5wdXRfMTVfSUkiLAogICAgICAgICAgICAgICAiaUJBUS5JbnB1dF8xNV9JSUkiLAogICAgICAgICAgICAgICAiaUJBUS5QVGV4X05vQ2xfSSIsCiAgICAgICAgICAgICAgICJpQkFRLlBUZXhfTm9DbF9JSSIsCiAgICAgICAgICAgICAgICJpQkFRLlBUZXhfTm9DbF9JSUkiLAogICAgICAgICAgICAgICAiaUJBUS5QVGV4XzAwMTVfSSIsCiAgICAgICAgICAgICAgICJpQkFRLlBUZXhfMDAxNV9JSSIsCiAgICAgICAgICAgICAgICJpQkFRLlBUZXhfMDAxNV9JSUkiLAogICAgICAgICAgICAgICAiaUJBUS5QVGV4XzAxNV9JIiwKICAgICAgICAgICAgICAgImlCQVEuUFRleF8wMTVfSUkiLAogICAgICAgICAgICAgICAiaUJBUS5QVGV4XzAxNV9JSUkiLAogICAgICAgICAgICAgICAiaUJBUS5QVGV4XzE1X0kiLAogICAgICAgICAgICAgICAiaUJBUS5QVGV4XzE1X0lJIiwKICAgICAgICAgICAgICAgImlCQVEuUFRleF8xNV9JSUkiKQpgYGAKCiMjIEZpcnN0IGZpbHRlcgoKSW4gYSBmaXJzdCBmaWx0ZXJpbmcgc3RlcCByZXZlcnNlIGFuZCBoaXRzIG9ubHkgaWRlbnRpZmllZCBieSBzaXRlIGFyZSByZW1vdmVkLgoKYGBge3IgZmlyc3QgZmlsdGVyIHN0ZXB9Ck1hc3NTcGVjRGF0YSA8LSBwcmltYXJ5TWFzc1NwZWNEYXRhW3ByaW1hcnlNYXNzU3BlY0RhdGEkUmV2ZXJzZSAhPSAiKyIsXQpNYXNzU3BlY0RhdGEgPC0gTWFzc1NwZWNEYXRhW01hc3NTcGVjRGF0YSRPbmx5LmlkZW50aWZpZWQuYnkuc2l0ZSAhPSAiKyIsXQpgYGAKCiMjIFRyYW5zZm9ybWF0aW9uCgpBbGwgaW50ZW5zaXRlcyBhcmUgbG9nMiB0cmFuc2Zvcm1lZC4KCmBgYHtyIGxvZzIgdHJhbnNmb3JtYXRpb259Ck1hc3NTcGVjRGF0YVtjKGludGVuc2l0aWVzLCBJQkFRLCBMRlEpXSA8LSBsb2cyKE1hc3NTcGVjRGF0YVtjKGludGVuc2l0aWVzLCBJQkFRLCBMRlEpXSkKYGBgCgpJbmZpbml0eSB2YWx1ZXMgdGhhdCByZXN1bHRlZCBmcm9tIGxvZzIgdHJhbnNmb3JtYXRpb24gYXJlIGNvbnZlcnRlZCB0byBgTkFgOgoKYGBge3IgY29udmVyc2lvbiBvZiBpbmZpbml0eSB2YWx1ZXN9CmlzLm5hKE1hc3NTcGVjRGF0YVtjKGludGVuc2l0aWVzLElCQVEsTEZRKV0pIDwtIHNhcHBseShNYXNzU3BlY0RhdGFbYyhpbnRlbnNpdGllcyxJQkFRLExGUSldLCBpcy5pbmZpbml0ZSkKYGBgCgojIyBUcnlwc2luIG5vcm1hbGlzYXRpb24gZmFjdG9yCgpUaGUgTEZRIGludGVuc2l0aWVzIGFyZSBub3JtYWxpc2VkIHVuZGVyIHRoZSBhc3N1bXB0aW9uIHRoYXQgbW9zdCBpbnRlbnNpdGllcyB3b24ndCBjaGFuZ2UuIFRoaXMgaXMgbm90IHRydWUgZm9yIFBUZXggZGF0YSwgc28gdGhhdCB0aGUgbm9ybWFsaXNhdGlvbiBoYXMgdG8gYmUgY29ycmVjdGVkLiBDb3JyZWN0aW9uIGlzIGRvbmUgYnkgdXNpbmcgdHJ5cHNpbiAoYWRkZWQgdG8gYWxsIHNhbXBsZXMgaW4gdGhlIHNhbWUgYW1vdW50KSB0byBjYWxjdWxhdGUgYSBub3JtYWxpc2F0aW9uIGZhY3Rvci4KCmBgYHtyIGNhbGN1bGF0aW9uIG9mIHRyeXBzaW4gbm9ybWFsaXNhdGlvbiBmYWN0b3J9CnRyeXBzaW4gPSBNYXNzU3BlY0RhdGFbTWFzc1NwZWNEYXRhJE1ham9yaXR5LnByb3RlaW4uSURzID09ICJDT05fX1AwMDc2MSIsXQoKdHJwX21lYW5fSW5wX05vQ2wgPC0gbWVhbih0cnlwc2luWyxMRlFbMV1dLHRyeXBzaW5bLExGUVsyXV0sdHJ5cHNpblssTEZRWzNdXSkKdHJwX21lYW5fSW5wXzAwMTUgPC0gbWVhbih0cnlwc2luWyxMRlFbNF1dLHRyeXBzaW5bLExGUVs1XV0sdHJ5cHNpblssTEZRWzZdXSkKdHJwX21lYW5fSW5wXzAxNSA8LSBtZWFuKHRyeXBzaW5bLExGUVs3XV0sdHJ5cHNpblssTEZRWzhdXSx0cnlwc2luWyxMRlFbOV1dKQp0cnBfbWVhbl9JbnBfMTUgPC0gbWVhbih0cnlwc2luWyxMRlFbMTBdXSx0cnlwc2luWyxMRlFbMTFdXSx0cnlwc2luWyxMRlFbMTJdXSkKCnRycF9tZWFuX1BUZXhfTm9DbCA8LSBtZWFuKHRyeXBzaW5bLExGUVsxM11dLHRyeXBzaW5bLExGUVsxNF1dLHRyeXBzaW5bLExGUVsxNV1dKQp0cnBfbWVhbl9QVGV4XzAwMTUgPC0gbWVhbih0cnlwc2luWyxMRlFbMTZdXSx0cnlwc2luWyxMRlFbMTddXSx0cnlwc2luWyxMRlFbMThdXSkKdHJwX21lYW5fUFRleF8wMTUgPC0gbWVhbih0cnlwc2luWyxMRlFbMTldXSx0cnlwc2luWyxMRlFbMjBdXSx0cnlwc2luWyxMRlFbMjFdXSkKdHJwX21lYW5fUFRleF8xNSA8LSBtZWFuKHRyeXBzaW5bLExGUVsyMl1dLHRyeXBzaW5bLExGUVsyM11dLHRyeXBzaW5bLExGUVsyNF1dKSAKCm5mX05vQ2wgPC0gdHJwX21lYW5fUFRleF9Ob0NsIC0gdHJwX21lYW5fSW5wX05vQ2wKbmZfMDAxNUNsIDwtIHRycF9tZWFuX1BUZXhfMDAxNSAtIHRycF9tZWFuX0lucF8wMDE1Cm5mXzAxNUNsIDwtIHRycF9tZWFuX1BUZXhfMDE1IC0gdHJwX21lYW5fSW5wXzAxNQpuZl8xNUNsIDwtIHRycF9tZWFuX1BUZXhfMTUgLSB0cnBfbWVhbl9JbnBfMTUKYGBgCgpUaWR5IHVwOgoKYGBge3J9CnJtKGxpc3QgPSBscyhwYXR0ZXJuID0gInRycF9tZWFuXyIpKQpybSh0cnlwc2luKQpgYGAKCgojIyBDb250YW1pbmFudCByZW1vdmFsCgpBZnRlciBjYWxjdWxhdGlvbiBvZiB0aGUgbm9ybWFsaXNhdGlvbiBmYWN0b3JzIHRoZSBjb250YW1pbmFudHMgYXJlIHJlbW92ZWQgZnJvbSB0aGUgZGF0YSBzZXQuCgpgYGB7ciBjb250YW1pbmFudCByZW1vdmFsfQpNYXNzU3BlY0RhdGEgPC0gTWFzc1NwZWNEYXRhW01hc3NTcGVjRGF0YSRQb3RlbnRpYWwuY29udGFtaW5hbnQgIT0gIisiLF0KYGBgCgojIyBUcnlwc2luIG5vcm1hbGlzYXRpb24KClRoZSBub3JtYWxpc2F0aW9uIGZhY3RvcnMgYXJlIGFwcGxpZWQgYnkgc3VidHJhY3Rpb24sIHNpbmNlIHRoZSBkYXRhIHdlcmUgYWxyZWFkeSBsb2cgdHJhbnNmb3JtZWQuCgpgYGB7ciB0cnlwc2luIG5vcm1hbGlzYXRpb259Cm5vcm1fTWFzc1NwZWNEYXRhIDwtIE1hc3NTcGVjRGF0YQoKbm9ybV9NYXNzU3BlY0RhdGFbTEZRWzEzOjE1XV0gPC0gbm9ybV9NYXNzU3BlY0RhdGFbTEZRWzEzOjE1XV0gLSBuZl9Ob0NsCm5vcm1fTWFzc1NwZWNEYXRhW0xGUVsxNjoxOF1dIDwtIG5vcm1fTWFzc1NwZWNEYXRhW0xGUVsxNjoxOF1dIC0gbmZfMDAxNUNsCm5vcm1fTWFzc1NwZWNEYXRhW0xGUVsxOToyMV1dIDwtIG5vcm1fTWFzc1NwZWNEYXRhW0xGUVsxOToyMV1dIC0gbmZfMDE1Q2wKbm9ybV9NYXNzU3BlY0RhdGFbTEZRWzIyOjI0XV0gPC0gbm9ybV9NYXNzU3BlY0RhdGFbTEZRWzIyOjI0XV0gLSBuZl8xNUNsCmBgYAoKVGlkeSB1cDoKCmBgYHtyfQpybShsaXN0ID0gbHMocGF0dGVybiA9ICJuZl8iKSkKYGBgCgoKIyMgSW5jb21wbGV0ZSBvYnNlcnZhdGlvbiByZW1vdmFsCgpXZSBvbmx5IHdhbnQgdG8gY29uc2lkZXIgdGhvc2UgcHJvdGVpbnMsIHRoYXQgd2VyZSBmb3VuZCBpbiBhbGwgcmVwbGljYXRlcyBvZiBhbGwgZXhwZXJpbWVudHMuCgpgYGB7ciByZW1vdmFsIG9mIGluY29tcGxldGUgb2JzZXJ2YXRpb25zfQpmb3IoZXhwIGluIGMoIlBUZXhfbm9DTCIsIlBUZXhfMDAxNSIsIlBUZXhfMDE1IiwiUFRleF8xNSIsICJJbnB1dF9ub0NMIiwiSW5wdXRfMDAxNSIsIklucHV0XzAxNSIsIklucHV0XzE1IikpewogIAogIHN1Yl9NYXNzU3BlY0RhdGEgPC0gc2VsZWN0KG5vcm1fTWFzc1NwZWNEYXRhLCBtYXRjaGVzKHBhc3RlKCJMRlEuaW50ZW5zaXR5IiwgZXhwLCBzZXAgPSAiLiIpKSkKICB4IDwtIGFwcGx5KHN1Yl9NYXNzU3BlY0RhdGEsIDEsIGZ1bmN0aW9uKHgpc3VtKCFpcy5uYSh4KSk+MikKICBub3JtX01hc3NTcGVjRGF0YSA8LSBub3JtX01hc3NTcGVjRGF0YVt4LF0KfQpgYGAKClRpZHkgdXA6CgpgYGB7cn0Kcm0obGlzdCA9IGxzKHBhdHRlcm4gPSAic3ViXyIpKQpybShleHApCnJtKHgpCmBgYAoKCiMjIFBsb3RzCgojIyMgSW50ZW5zaXRpZXMgU2NhdHRlcnBsb3RzCgpgYGB7cn0KIyMgSW5wdXQgbm9DTAppbnB1dF9ub2NsX3NjYXR0ZXIgPC0gZ2dwYWlycyhub3JtX01hc3NTcGVjRGF0YVssTEZRWzE6M11dLCAKICAgICAgICBsb3dlciA9IGxpc3QoY29udGludW91cyA9IHdyYXAoImNvciIsIHNpemUgPSA3KSksIAogICAgICAgIHVwcGVyID0gbGlzdChjb250aW51b3VzID0gInNtb290aCIpLAogICAgICAgIGRpYWcgPSBsaXN0KGNvbnRpbnVvdXMgPSAiZGVuc2l0eURpYWciKSkgKyBzY2F0dGVyX3RoZW1lCgoKIyMgSW5wdXQgMC4wMTUKaW5wdXRfMDAxNV9zY2F0dGVyIDwtIGdncGFpcnMobm9ybV9NYXNzU3BlY0RhdGFbLExGUVs0OjZdXSwgCiAgICAgICAgbG93ZXIgPSBsaXN0KGNvbnRpbnVvdXMgPSB3cmFwKCJjb3IiLCBzaXplID0gNykpLCAKICAgICAgICB1cHBlciA9IGxpc3QoY29udGludW91cyA9ICJzbW9vdGgiKSwKICAgICAgICBkaWFnID0gbGlzdChjb250aW51b3VzID0gImRlbnNpdHlEaWFnIikpICsgc2NhdHRlcl90aGVtZQoKCiMjIElucHV0IDAuMTUKaW5wdXRfMDE1X3NjYXR0ZXIgPC0gZ2dwYWlycyhub3JtX01hc3NTcGVjRGF0YVssTEZRWzc6OV1dLCAKICAgICAgICBsb3dlciA9IGxpc3QoY29udGludW91cyA9IHdyYXAoImNvciIsIHNpemUgPSA3KSksIAogICAgICAgIHVwcGVyID0gbGlzdChjb250aW51b3VzID0gInNtb290aCIpLAogICAgICAgIGRpYWcgPSBsaXN0KGNvbnRpbnVvdXMgPSAiZGVuc2l0eURpYWciKSkgICsgc2NhdHRlcl90aGVtZQoKCiMjIElucHV0IDEuNQppbnB1dF8xNV9zY2F0dGVyIDwtIGdncGFpcnMobm9ybV9NYXNzU3BlY0RhdGFbLExGUVsxMDoxMl1dLCAKICAgICAgICBsb3dlciA9IGxpc3QoY29udGludW91cyA9IHdyYXAoImNvciIsIHNpemUgPSA3KSksIAogICAgICAgIHVwcGVyID0gbGlzdChjb250aW51b3VzID0gInNtb290aCIpLAogICAgICAgIGRpYWcgPSBsaXN0KGNvbnRpbnVvdXMgPSAiZGVuc2l0eURpYWciKSkgICsgc2NhdHRlcl90aGVtZQoKCiMjIFBUZXggbm9DTApwdGV4X25vY2xfc2NhdHRlciA8LSBnZ3BhaXJzKG5vcm1fTWFzc1NwZWNEYXRhWyxMRlFbMTM6MTVdXSwgCiAgICAgICAgbG93ZXIgPSBsaXN0KGNvbnRpbnVvdXMgPSB3cmFwKCJjb3IiLCBzaXplID0gNykpLCAKICAgICAgICB1cHBlciA9IGxpc3QoY29udGludW91cyA9ICJzbW9vdGgiKSwKICAgICAgICBkaWFnID0gbGlzdChjb250aW51b3VzID0gImRlbnNpdHlEaWFnIikpICArIHNjYXR0ZXJfdGhlbWUKCiMjIFBUZXggMC4wMTUKcHRleF8wMDE1X3NjYXR0ZXIgPC0gZ2dwYWlycyhub3JtX01hc3NTcGVjRGF0YVssTEZRWzE2OjE4XV0sIAogICAgICAgIGxvd2VyID0gbGlzdChjb250aW51b3VzID0gd3JhcCgiY29yIiwgc2l6ZSA9IDcpKSwgCiAgICAgICAgdXBwZXIgPSBsaXN0KGNvbnRpbnVvdXMgPSAic21vb3RoIiksCiAgICAgICAgZGlhZyA9IGxpc3QoY29udGludW91cyA9ICJkZW5zaXR5RGlhZyIpKSAgKyBzY2F0dGVyX3RoZW1lCgoKIyMgUFRleCAwLjE1CnB0ZXhfMDE1X3NjYXR0ZXIgPC0gZ2dwYWlycyhub3JtX01hc3NTcGVjRGF0YVssTEZRWzE5OjIxXV0sIAogICAgICAgIGxvd2VyID0gbGlzdChjb250aW51b3VzID0gd3JhcCgiY29yIiwgc2l6ZSA9IDcpKSwgCiAgICAgICAgdXBwZXIgPSBsaXN0KGNvbnRpbnVvdXMgPSAic21vb3RoIiksCiAgICAgICAgZGlhZyA9IGxpc3QoY29udGludW91cyA9ICJkZW5zaXR5RGlhZyIpKSAgKyBzY2F0dGVyX3RoZW1lCgoKIyMgUFRleCAxLjUKcHRleF8xNV9zY2F0dGVyIDwtIGdncGFpcnMobm9ybV9NYXNzU3BlY0RhdGFbLExGUVsyMjoyNF1dLAogICAgICAgIGxvd2VyID0gbGlzdChjb250aW51b3VzID0gd3JhcCgiY29yIiwgc2l6ZSA9IDcpKSwgCiAgICAgICAgdXBwZXIgPSBsaXN0KGNvbnRpbnVvdXMgPSAic21vb3RoIiksCiAgICAgICAgZGlhZyA9IGxpc3QoY29udGludW91cyA9ICJkZW5zaXR5RGlhZyIpKSAgKyBzY2F0dGVyX3RoZW1lCgpgYGAKCmBgYHtyLCBmaWcud2lkdGg9MTIsIGZpZy5oZWlnaHQ9N30KaW5wdXRfbm9jbF9zY2F0dGVyCmlucHV0XzAwMTVfc2NhdHRlcgppbnB1dF8wMTVfc2NhdHRlcgppbnB1dF8xNV9zY2F0dGVyCgpwdGV4X25vY2xfc2NhdHRlcgpwdGV4XzAwMTVfc2NhdHRlcgpwdGV4XzAxNV9zY2F0dGVyCnB0ZXhfMTVfc2NhdHRlcgpgYGAKClRpZHkgdXA6CgpgYGB7cn0Kcm0obGlzdCA9IGxzKHBhdHRlcm4gPSAiX3NjYXR0ZXIiKSkKYGBgCgoKIyBFbnJpY2htZW50IGFuYWx5c2lzCgpBZnRlciBwcmVwYXJhdGlvbiB0aGUgZm9sZCBjaGFuZ2VzIGJldHdlZW4gcmVzcGVjdGl2ZSBJbnB1dC9QVGV4IHBhaXJzIGNhbiBiZSBjYWxjdWxhdGVkIGFuZCBhIG1vZGVyYXRlZCB0LXRlc3Qgd2l0aCBmb2xsb3dpbmcgQmVuamFtaW5pLUhvY2hiZXJnIGNvcnJlY3Rpb24gaXMgdXNlZCB0byBkZXRlcm1pbmUgdGhlIGZhbHNlIGRpc2NvdmVyeSByYXRlIChGRFIpLgoKIyMgRm9sZCBDaGFuZ2VzCgpUaGUgZm9sZCBjaGFuZ2VzIGFyZSBjYWxjdWxhdGVkIGJ5IHN1YnRyYWN0aW5nIHRoZSBsb2ctdHJhbnNmb3JtZWQgTEZRIGludGVuc2l0eSB2YWx1ZXMgb2YgdGhlIG5vbi1jcm9zc2xpbmtlZCAoLUNMKSBmcm9tIHRoZSBjcm9zc2xpbmtlZCAoK0NMKSBzYW1wbGVzLgoKYGBge3IgY2FsY3VsYXRpb24gb2YgaW5kaXZpZHVhbCBmb2xkIGNoYW5nZXN9Cm5vcm1fTWFzc1NwZWNEYXRhJEZDLklucHV0XzAwMTUucmVwMSA8LSAobm9ybV9NYXNzU3BlY0RhdGEkTEZRLmludGVuc2l0eS5JbnB1dF8wMDE1X0kgLSBub3JtX01hc3NTcGVjRGF0YSRMRlEuaW50ZW5zaXR5LklucHV0X05vQ2xfSSkKbm9ybV9NYXNzU3BlY0RhdGEkRkMuSW5wdXRfMDAxNS5yZXAyIDwtIChub3JtX01hc3NTcGVjRGF0YSRMRlEuaW50ZW5zaXR5LklucHV0XzAwMTVfSUkgLSBub3JtX01hc3NTcGVjRGF0YSRMRlEuaW50ZW5zaXR5LklucHV0X05vQ2xfSUkpCm5vcm1fTWFzc1NwZWNEYXRhJEZDLklucHV0XzAwMTUucmVwMyA8LSAobm9ybV9NYXNzU3BlY0RhdGEkTEZRLmludGVuc2l0eS5JbnB1dF8wMDE1X0lJSSAtIG5vcm1fTWFzc1NwZWNEYXRhJExGUS5pbnRlbnNpdHkuSW5wdXRfTm9DbF9JSUkpCgpub3JtX01hc3NTcGVjRGF0YSRGQy5JbnB1dF8wMTUucmVwMSA8LSAobm9ybV9NYXNzU3BlY0RhdGEkTEZRLmludGVuc2l0eS5JbnB1dF8wMTVfSSAtIG5vcm1fTWFzc1NwZWNEYXRhJExGUS5pbnRlbnNpdHkuSW5wdXRfTm9DbF9JKQpub3JtX01hc3NTcGVjRGF0YSRGQy5JbnB1dF8wMTUucmVwMiA8LSAobm9ybV9NYXNzU3BlY0RhdGEkTEZRLmludGVuc2l0eS5JbnB1dF8wMTVfSUkgLSBub3JtX01hc3NTcGVjRGF0YSRMRlEuaW50ZW5zaXR5LklucHV0X05vQ2xfSUkpCm5vcm1fTWFzc1NwZWNEYXRhJEZDLklucHV0XzAxNS5yZXAzIDwtIChub3JtX01hc3NTcGVjRGF0YSRMRlEuaW50ZW5zaXR5LklucHV0XzAxNV9JSUkgLSBub3JtX01hc3NTcGVjRGF0YSRMRlEuaW50ZW5zaXR5LklucHV0X05vQ2xfSUlJKQoKbm9ybV9NYXNzU3BlY0RhdGEkRkMuSW5wdXRfMTUucmVwMSA8LSAobm9ybV9NYXNzU3BlY0RhdGEkTEZRLmludGVuc2l0eS5JbnB1dF8xNV9JIC0gbm9ybV9NYXNzU3BlY0RhdGEkTEZRLmludGVuc2l0eS5JbnB1dF9Ob0NsX0kpCm5vcm1fTWFzc1NwZWNEYXRhJEZDLklucHV0XzE1LnJlcDIgPC0gKG5vcm1fTWFzc1NwZWNEYXRhJExGUS5pbnRlbnNpdHkuSW5wdXRfMTVfSUkgLSBub3JtX01hc3NTcGVjRGF0YSRMRlEuaW50ZW5zaXR5LklucHV0X05vQ2xfSUkpCm5vcm1fTWFzc1NwZWNEYXRhJEZDLklucHV0XzE1LnJlcDMgPC0gKG5vcm1fTWFzc1NwZWNEYXRhJExGUS5pbnRlbnNpdHkuSW5wdXRfMTVfSUlJIC0gbm9ybV9NYXNzU3BlY0RhdGEkTEZRLmludGVuc2l0eS5JbnB1dF9Ob0NsX0lJSSkKCm5vcm1fTWFzc1NwZWNEYXRhJEZDLlBUZXhfMDAxNS5yZXAxIDwtIChub3JtX01hc3NTcGVjRGF0YSRMRlEuaW50ZW5zaXR5LlBUZXhfMDAxNV9JIC0gbm9ybV9NYXNzU3BlY0RhdGEkTEZRLmludGVuc2l0eS5QVGV4X05vQ2xfSSkKbm9ybV9NYXNzU3BlY0RhdGEkRkMuUFRleF8wMDE1LnJlcDIgPC0gKG5vcm1fTWFzc1NwZWNEYXRhJExGUS5pbnRlbnNpdHkuUFRleF8wMDE1X0lJIC0gbm9ybV9NYXNzU3BlY0RhdGEkTEZRLmludGVuc2l0eS5QVGV4X05vQ2xfSUkpCm5vcm1fTWFzc1NwZWNEYXRhJEZDLlBUZXhfMDAxNS5yZXAzIDwtIChub3JtX01hc3NTcGVjRGF0YSRMRlEuaW50ZW5zaXR5LlBUZXhfMDAxNV9JSUkgLSBub3JtX01hc3NTcGVjRGF0YSRMRlEuaW50ZW5zaXR5LlBUZXhfTm9DbF9JSUkpCgpub3JtX01hc3NTcGVjRGF0YSRGQy5QVGV4XzAxNS5yZXAxIDwtIChub3JtX01hc3NTcGVjRGF0YSRMRlEuaW50ZW5zaXR5LlBUZXhfMDE1X0kgLSBub3JtX01hc3NTcGVjRGF0YSRMRlEuaW50ZW5zaXR5LlBUZXhfTm9DbF9JKQpub3JtX01hc3NTcGVjRGF0YSRGQy5QVGV4XzAxNS5yZXAyIDwtIChub3JtX01hc3NTcGVjRGF0YSRMRlEuaW50ZW5zaXR5LlBUZXhfMDE1X0lJIC0gbm9ybV9NYXNzU3BlY0RhdGEkTEZRLmludGVuc2l0eS5QVGV4X05vQ2xfSUkpCm5vcm1fTWFzc1NwZWNEYXRhJEZDLlBUZXhfMDE1LnJlcDMgPC0gKG5vcm1fTWFzc1NwZWNEYXRhJExGUS5pbnRlbnNpdHkuUFRleF8wMTVfSUlJIC0gbm9ybV9NYXNzU3BlY0RhdGEkTEZRLmludGVuc2l0eS5QVGV4X05vQ2xfSUlJKQoKbm9ybV9NYXNzU3BlY0RhdGEkRkMuUFRleF8xNS5yZXAxIDwtIChub3JtX01hc3NTcGVjRGF0YSRMRlEuaW50ZW5zaXR5LlBUZXhfMTVfSSAtIG5vcm1fTWFzc1NwZWNEYXRhJExGUS5pbnRlbnNpdHkuUFRleF9Ob0NsX0kpCm5vcm1fTWFzc1NwZWNEYXRhJEZDLlBUZXhfMTUucmVwMiA8LSAobm9ybV9NYXNzU3BlY0RhdGEkTEZRLmludGVuc2l0eS5QVGV4XzE1X0lJIC0gbm9ybV9NYXNzU3BlY0RhdGEkTEZRLmludGVuc2l0eS5QVGV4X05vQ2xfSUkpCm5vcm1fTWFzc1NwZWNEYXRhJEZDLlBUZXhfMTUucmVwMyA8LSAobm9ybV9NYXNzU3BlY0RhdGEkTEZRLmludGVuc2l0eS5QVGV4XzE1X0lJSSAtIG5vcm1fTWFzc1NwZWNEYXRhJExGUS5pbnRlbnNpdHkuUFRleF9Ob0NsX0lJSSkKCgpgYGAKCk1lYW4gZm9sZCBjaGFuZ2VzIGFyZSBjYWxjdWxhdGVkLgoKYGBge3IgY2FsY3VsYXRpb24gb2YgbWVhbiBmb2xkIGNoYW5nZXN9Cm5vcm1fTWFzc1NwZWNEYXRhJEZDLklucHV0XzAwMTUubWVhbiA8LSByb3dNZWFucyhub3JtX01hc3NTcGVjRGF0YVssMjUxOjI1M10pCm5vcm1fTWFzc1NwZWNEYXRhJEZDLklucHV0XzAxNS5tZWFuIDwtIHJvd01lYW5zKG5vcm1fTWFzc1NwZWNEYXRhWywyNTQ6MjU2XSkKbm9ybV9NYXNzU3BlY0RhdGEkRkMuSW5wdXRfMTUubWVhbiA8LSByb3dNZWFucyhub3JtX01hc3NTcGVjRGF0YVssMjU3OjI1OV0pCm5vcm1fTWFzc1NwZWNEYXRhJEZDLlBUZXhfMDAxNS5tZWFuIDwtIHJvd01lYW5zKG5vcm1fTWFzc1NwZWNEYXRhWywyNjA6MjYyXSkKbm9ybV9NYXNzU3BlY0RhdGEkRkMuUFRleF8wMTUubWVhbiA8LSByb3dNZWFucyhub3JtX01hc3NTcGVjRGF0YVssMjYzOjI2NV0pCm5vcm1fTWFzc1NwZWNEYXRhJEZDLlBUZXhfMTUubWVhbiA8LSByb3dNZWFucyhub3JtX01hc3NTcGVjRGF0YVssMjY2OjI2OF0pCmBgYAoKIyMgTW9kZXJhdGVkIHQtdGVzdCBhbmQgQmVuamFtaW5pLUhvY2hiZXJnIGNvcnJlY3Rpb24KCkEgbW9kZXJhdGVkIHQtdGVzdCBpbmNsdWRpbmcgQmVuamFtaW5pLUhvY2hiZXJnIHAtdmFsdWUgY29ycmVjdGlvbiBpcyBsYXllZCBvdXQgb24gdGhlIGZvbGQgY2hhbmdlcy4KCmBgYHtyIG1vZCB0LXRlc3QgYW5kIEJIIGNvcnJlY3Rpb259CnB2YWwgPSAgZUJheWVzKGxtRml0KG5vcm1fTWFzc1NwZWNEYXRhWyxncmVwKCJGQy5JbnB1dF8wMDE1LnJlcCIsbmFtZXMobm9ybV9NYXNzU3BlY0RhdGEpKV0pKQpub3JtX01hc3NTcGVjRGF0YSRwdmFsLklucHV0XzAwMTUgPC0gcHZhbCRwLnZhbHVlCm5vcm1fTWFzc1NwZWNEYXRhJHBhZGouSW5wdXRfMDAxNSA8LSAgcC5hZGp1c3Qobm9ybV9NYXNzU3BlY0RhdGEkcHZhbC5JbnB1dF8wMDE1LCBtZXRob2Q9IkJIIikKCnB2YWwgPSAgZUJheWVzKGxtRml0KG5vcm1fTWFzc1NwZWNEYXRhWyxncmVwKCJGQy5JbnB1dF8wMTUucmVwIixuYW1lcyhub3JtX01hc3NTcGVjRGF0YSkpXSkpCm5vcm1fTWFzc1NwZWNEYXRhJHB2YWwuSW5wdXRfMDE1IDwtIHB2YWwkcC52YWx1ZQpub3JtX01hc3NTcGVjRGF0YSRwYWRqLklucHV0XzAxNSA8LSAgcC5hZGp1c3Qobm9ybV9NYXNzU3BlY0RhdGEkcHZhbC5JbnB1dF8wMTUsIG1ldGhvZD0iQkgiKQoKcHZhbCA9ICBlQmF5ZXMobG1GaXQobm9ybV9NYXNzU3BlY0RhdGFbLGdyZXAoIkZDLklucHV0XzE1LnJlcCIsbmFtZXMobm9ybV9NYXNzU3BlY0RhdGEpKV0pKQpub3JtX01hc3NTcGVjRGF0YSRwdmFsLklucHV0XzE1IDwtIHB2YWwkcC52YWx1ZQpub3JtX01hc3NTcGVjRGF0YSRwYWRqLklucHV0XzE1IDwtICBwLmFkanVzdChub3JtX01hc3NTcGVjRGF0YSRwdmFsLklucHV0XzE1LCBtZXRob2Q9IkJIIikKCnB2YWwgPSBlQmF5ZXMobG1GaXQobm9ybV9NYXNzU3BlY0RhdGFbLGdyZXAoIkZDLlBUZXhfMDAxNS5yZXAiLG5hbWVzKG5vcm1fTWFzc1NwZWNEYXRhKSldKSkKbm9ybV9NYXNzU3BlY0RhdGEkcHZhbC5QVGV4XzAwMTUgPC0gcHZhbCRwLnZhbHVlCm5vcm1fTWFzc1NwZWNEYXRhJHBhZGouUFRleF8wMDE1IDwtIHAuYWRqdXN0KG5vcm1fTWFzc1NwZWNEYXRhJHB2YWwuUFRleF8wMDE1LCBtZXRob2Q9IkJIIikKCnB2YWwgPSBlQmF5ZXMobG1GaXQobm9ybV9NYXNzU3BlY0RhdGFbLGdyZXAoIkZDLlBUZXhfMDE1LnJlcCIsbmFtZXMobm9ybV9NYXNzU3BlY0RhdGEpKV0pKQpub3JtX01hc3NTcGVjRGF0YSRwdmFsLlBUZXhfMDE1IDwtIHB2YWwkcC52YWx1ZQpub3JtX01hc3NTcGVjRGF0YSRwYWRqLlBUZXhfMDE1IDwtIHAuYWRqdXN0KG5vcm1fTWFzc1NwZWNEYXRhJHB2YWwuUFRleF8wMTUsIG1ldGhvZD0iQkgiKQoKcHZhbCA9IGVCYXllcyhsbUZpdChub3JtX01hc3NTcGVjRGF0YVssZ3JlcCgiRkMuUFRleF8xNS5yZXAiLG5hbWVzKG5vcm1fTWFzc1NwZWNEYXRhKSldKSkKbm9ybV9NYXNzU3BlY0RhdGEkcHZhbC5QVGV4XzE1IDwtIHB2YWwkcC52YWx1ZQpub3JtX01hc3NTcGVjRGF0YSRwYWRqLlBUZXhfMTUgPC0gcC5hZGp1c3Qobm9ybV9NYXNzU3BlY0RhdGEkcHZhbC5QVGV4XzE1LCBtZXRob2Q9IkJIIikKYGBgCgpUaWR5IHVwOgoKYGBge3J9CnJtKHB2YWwpCmBgYAoKCiMjIE1lYW4gaW50ZW5zaXRpZXMKCk1lYW4gaW50ZW5zaXRpZXMgKExGUSBhbmQgaUJBUSkgYXJlIGNhbGN1bGF0ZWQuCgpgYGB7cn0KIyMjIExGUQpub3JtX01hc3NTcGVjRGF0YSRMRlEuaW50ZW5zaXR5LklucHV0X25vQ2xfbWVhbiA8LSByb3dNZWFucyhub3JtX01hc3NTcGVjRGF0YVssYygiTEZRLmludGVuc2l0eS5JbnB1dF9Ob0NsX0kiLCAiTEZRLmludGVuc2l0eS5JbnB1dF9Ob0NsX0lJIiwgIkxGUS5pbnRlbnNpdHkuSW5wdXRfTm9DbF9JSUkiKV0sIG5hLnJtID0gRkFMU0UpCm5vcm1fTWFzc1NwZWNEYXRhJExGUS5pbnRlbnNpdHkuSW5wdXRfMDAxNV9tZWFuIDwtIHJvd01lYW5zKG5vcm1fTWFzc1NwZWNEYXRhWyxjKCJMRlEuaW50ZW5zaXR5LklucHV0XzAwMTVfSSIsICJMRlEuaW50ZW5zaXR5LklucHV0XzAwMTVfSUkiLCAiTEZRLmludGVuc2l0eS5JbnB1dF8wMDE1X0lJSSIpXSwgbmEucm0gPSBGQUxTRSkKbm9ybV9NYXNzU3BlY0RhdGEkTEZRLmludGVuc2l0eS5JbnB1dF8wMTVfbWVhbiA8LSByb3dNZWFucyhub3JtX01hc3NTcGVjRGF0YVssYygiTEZRLmludGVuc2l0eS5JbnB1dF8wMTVfSSIsICJMRlEuaW50ZW5zaXR5LklucHV0XzAxNV9JSSIsICJMRlEuaW50ZW5zaXR5LklucHV0XzAxNV9JSUkiKV0sIG5hLnJtID0gRkFMU0UpCm5vcm1fTWFzc1NwZWNEYXRhJExGUS5pbnRlbnNpdHkuSW5wdXRfMTVfbWVhbiA8LSByb3dNZWFucyhub3JtX01hc3NTcGVjRGF0YVssYygiTEZRLmludGVuc2l0eS5JbnB1dF8xNV9JIiwgIkxGUS5pbnRlbnNpdHkuSW5wdXRfMTVfSUkiLCAiTEZRLmludGVuc2l0eS5JbnB1dF8xNV9JSUkiKV0sIG5hLnJtID0gRkFMU0UpCm5vcm1fTWFzc1NwZWNEYXRhJExGUS5pbnRlbnNpdHkuUFRleF9ub0NsX21lYW4gPC0gcm93TWVhbnMobm9ybV9NYXNzU3BlY0RhdGFbLGMoIkxGUS5pbnRlbnNpdHkuUFRleF9Ob0NsX0kiLCAiTEZRLmludGVuc2l0eS5QVGV4X05vQ2xfSUkiLCAiTEZRLmludGVuc2l0eS5QVGV4X05vQ2xfSUlJIildLCBuYS5ybSA9IEZBTFNFKQpub3JtX01hc3NTcGVjRGF0YSRMRlEuaW50ZW5zaXR5LlBUZXhfMDAxNV9tZWFuIDwtIHJvd01lYW5zKG5vcm1fTWFzc1NwZWNEYXRhWyxjKCJMRlEuaW50ZW5zaXR5LlBUZXhfMDAxNV9JIiwgIkxGUS5pbnRlbnNpdHkuUFRleF8wMDE1X0lJIiwgIkxGUS5pbnRlbnNpdHkuUFRleF8wMDE1X0lJSSIpXSwgbmEucm0gPSBGQUxTRSkKbm9ybV9NYXNzU3BlY0RhdGEkTEZRLmludGVuc2l0eS5QVGV4XzAxNV9tZWFuIDwtIHJvd01lYW5zKG5vcm1fTWFzc1NwZWNEYXRhWyxjKCJMRlEuaW50ZW5zaXR5LlBUZXhfMDE1X0kiLCAiTEZRLmludGVuc2l0eS5QVGV4XzAxNV9JSSIsICJMRlEuaW50ZW5zaXR5LlBUZXhfMDE1X0lJSSIpXSwgbmEucm0gPSBGQUxTRSkKbm9ybV9NYXNzU3BlY0RhdGEkTEZRLmludGVuc2l0eS5QVGV4XzE1X21lYW4gPC0gcm93TWVhbnMobm9ybV9NYXNzU3BlY0RhdGFbLGMoIkxGUS5pbnRlbnNpdHkuUFRleF8xNV9JIiwgIkxGUS5pbnRlbnNpdHkuUFRleF8xNV9JSSIsICJMRlEuaW50ZW5zaXR5LlBUZXhfMTVfSUlJIildLCBuYS5ybSA9IEZBTFNFKQoKIyMjIGlCQVEKbm9ybV9NYXNzU3BlY0RhdGEkaUJBUS5JbnB1dF9ub0NsX21lYW4gPC0gcm93TWVhbnMobm9ybV9NYXNzU3BlY0RhdGFbLGMoImlCQVEuSW5wdXRfTm9DbF9JIiwgImlCQVEuSW5wdXRfTm9DbF9JSSIsICJpQkFRLklucHV0X05vQ2xfSUlJIildLCBuYS5ybSA9IEZBTFNFKQpub3JtX01hc3NTcGVjRGF0YSRpQkFRLklucHV0XzAwMTVfbWVhbiA8LSByb3dNZWFucyhub3JtX01hc3NTcGVjRGF0YVssYygiaUJBUS5JbnB1dF8wMDE1X0kiLCAiaUJBUS5JbnB1dF8wMDE1X0lJIiwgImlCQVEuSW5wdXRfMDAxNV9JSUkiKV0sIG5hLnJtID0gRkFMU0UpCm5vcm1fTWFzc1NwZWNEYXRhJGlCQVEuSW5wdXRfMDE1X21lYW4gPC0gcm93TWVhbnMobm9ybV9NYXNzU3BlY0RhdGFbLGMoImlCQVEuSW5wdXRfMDE1X0kiLCAiaUJBUS5JbnB1dF8wMTVfSUkiLCAiaUJBUS5JbnB1dF8wMTVfSUlJIildLCBuYS5ybSA9IEZBTFNFKQpub3JtX01hc3NTcGVjRGF0YSRpQkFRLklucHV0XzE1X21lYW4gPC0gcm93TWVhbnMobm9ybV9NYXNzU3BlY0RhdGFbLGMoImlCQVEuSW5wdXRfMTVfSSIsICJpQkFRLklucHV0XzE1X0lJIiwgImlCQVEuSW5wdXRfMTVfSUlJIildLCBuYS5ybSA9IEZBTFNFKQpub3JtX01hc3NTcGVjRGF0YSRpQkFRLlBUZXhfbm9DbF9tZWFuIDwtIHJvd01lYW5zKG5vcm1fTWFzc1NwZWNEYXRhWyxjKCJpQkFRLlBUZXhfTm9DbF9JIiwgImlCQVEuUFRleF9Ob0NsX0lJIiwgImlCQVEuUFRleF9Ob0NsX0lJSSIpXSwgbmEucm0gPSBGQUxTRSkKbm9ybV9NYXNzU3BlY0RhdGEkaUJBUS5QVGV4XzAwMTVfbWVhbiA8LSByb3dNZWFucyhub3JtX01hc3NTcGVjRGF0YVssYygiaUJBUS5QVGV4XzAwMTVfSSIsICJpQkFRLlBUZXhfMDAxNV9JSSIsICJpQkFRLlBUZXhfMDAxNV9JSUkiKV0sIG5hLnJtID0gRkFMU0UpCm5vcm1fTWFzc1NwZWNEYXRhJGlCQVEuUFRleF8wMTVfbWVhbiA8LSByb3dNZWFucyhub3JtX01hc3NTcGVjRGF0YVssYygiaUJBUS5QVGV4XzAxNV9JIiwgImlCQVEuUFRleF8wMTVfSUkiLCAiaUJBUS5QVGV4XzAxNV9JSUkiKV0sIG5hLnJtID0gRkFMU0UpCm5vcm1fTWFzc1NwZWNEYXRhJGlCQVEuUFRleF8xNV9tZWFuIDwtIHJvd01lYW5zKG5vcm1fTWFzc1NwZWNEYXRhWyxjKCJpQkFRLlBUZXhfMTVfSSIsICJpQkFRLlBUZXhfMTVfSUkiLCAiaUJBUS5QVGV4XzE1X0lJSSIpXSwgbmEucm0gPSBGQUxTRSkKYGBgCgoKIyMgRkRSIGFuZCBGQyBmaWx0ZXIgc3Vic2V0CgpXZSB3YW50IHRvIGFkZGl0aW9uYWxseSBleGNsdWRlIHByb3RlaW5zIHRoYXQgYXJlIG5vdCBzaWduaWZpY2FudGx5IGVucmljaGVkIGluIGFsbCAzIGxpYnJhcmllcyBhbmQgdGhvc2UsIHdoaWNoIGFyZSBzaWduaWZpY2FudGx5IGRlcGxldGVkOgoKYGBge3J9CnNpZ19ub3JtX01hc3NTcGVjRGF0YSA8LSBub3JtX01hc3NTcGVjRGF0YVtub3JtX01hc3NTcGVjRGF0YSRwYWRqLlBUZXhfMDAxNSA8PSAwLjAxICYgbm9ybV9NYXNzU3BlY0RhdGEkcGFkai5QVGV4XzAxNSA8PSAwLjAxICYgbm9ybV9NYXNzU3BlY0RhdGEkcGFkai5QVGV4XzE1IDw9IDAuMDEsXQpkaW0oc2lnX25vcm1fTWFzc1NwZWNEYXRhKQpgYGAKCmBgYHtyfQpzaWdfbm9ybV9NYXNzU3BlY0RhdGEgPC0gc2lnX25vcm1fTWFzc1NwZWNEYXRhW3NpZ19ub3JtX01hc3NTcGVjRGF0YSRGQy5QVGV4XzAwMTUubWVhbiA+IDAgJiBzaWdfbm9ybV9NYXNzU3BlY0RhdGEkRkMuUFRleF8wMTUubWVhbiA+IDAgJiBzaWdfbm9ybV9NYXNzU3BlY0RhdGEkRkMuUFRleF8xNS5tZWFuID4gMCxdCmRpbShzaWdfbm9ybV9NYXNzU3BlY0RhdGEpCmBgYAoKCiMjIFBsb3RzCgojIyMgRW5yaWNobWVudHMKCmBgYHtyfQpGQyA8LSBjb2xuYW1lcyhub3JtX01hc3NTcGVjRGF0YSlbMjUxOjI2OF0KCiMjIElucHV0IDAuMDE1CnBhZGpfY28gPC0gbm9ybV9NYXNzU3BlY0RhdGEkcGFkai5JbnB1dF8wMDE1IDw9IDAuMDEKcGFkal9jb1t3aGljaChwYWRqX2NvKV0gPC0gInNpZ25pZmljYW50IgpwYWRqX2NvW3doaWNoKHBhZGpfY289PUZBTFNFKV0gPC0gIm5vbi1zaWduaWZpY2FudCIKCmZjX2lucHV0XzAwMTVfc2NhdHRlciA8LSBnZ3BhaXJzKGNiaW5kKG5vcm1fTWFzc1NwZWNEYXRhWyxGQ1sxOjNdXSwgcGFkal9jbyksCiAgICAgICAgY29sdW1ucyA9IDE6MywKICAgICAgICBtYXBwaW5nID0gZ2dwbG90Mjo6YWVzKGNvbG91ciA9IHBhZGpfY28sIGFscGhhID0gMC41KSwKICAgICAgICBsb3dlciA9IGxpc3QoY29udGludW91cyA9IHdyYXAoImNvciIsIHNpemUgPSA1KSksIAogICAgICAgIHVwcGVyID0gbGlzdChjb250aW51b3VzID0gInNtb290aCIpLAogICAgICAgIGRpYWcgPSBsaXN0KGNvbnRpbnVvdXMgPSAiZGVuc2l0eURpYWciKSkgKyBzY2F0dGVyX3RoZW1lCgoKIyMgSW5wdXQgMC4xNQpwYWRqX2NvIDwtIG5vcm1fTWFzc1NwZWNEYXRhJHBhZGouSW5wdXRfMDE1IDw9IDAuMDEKcGFkal9jb1t3aGljaChwYWRqX2NvKV0gPC0gInNpZ25pZmljYW50IgpwYWRqX2NvW3doaWNoKHBhZGpfY289PUZBTFNFKV0gPC0gIm5vbi1zaWduaWZpY2FudCIKCmZjX2lucHV0XzAxNV9zY2F0dGVyIDwtIGdncGFpcnMoY2JpbmQobm9ybV9NYXNzU3BlY0RhdGFbLEZDWzQ6Nl1dLCBwYWRqX2NvKSwKICAgICAgICBjb2x1bW5zID0gMTozLAogICAgICAgIG1hcHBpbmcgPSBnZ3Bsb3QyOjphZXMoY29sb3VyID0gcGFkal9jbywgYWxwaGEgPSAwLjUpLAogICAgICAgIGxvd2VyID0gbGlzdChjb250aW51b3VzID0gd3JhcCgiY29yIiwgc2l6ZSA9IDUpKSwgCiAgICAgICAgdXBwZXIgPSBsaXN0KGNvbnRpbnVvdXMgPSAic21vb3RoIiksCiAgICAgICAgZGlhZyA9IGxpc3QoY29udGludW91cyA9ICJkZW5zaXR5RGlhZyIpKSAgKyBzY2F0dGVyX3RoZW1lCgoKIyMgSW5wdXQgMS41CnBhZGpfY28gPC0gbm9ybV9NYXNzU3BlY0RhdGEkcGFkai5JbnB1dF8xNSA8PSAwLjAxCnBhZGpfY29bd2hpY2gocGFkal9jbyldIDwtICJzaWduaWZpY2FudCIKcGFkal9jb1t3aGljaChwYWRqX2NvPT1GQUxTRSldIDwtICJub24tc2lnbmlmaWNhbnQiCgpmY19pbnB1dF8xNV9zY2F0dGVyIDwtIGdncGFpcnMoY2JpbmQobm9ybV9NYXNzU3BlY0RhdGFbLEZDWzc6OV1dLCBwYWRqX2NvKSwKICAgICAgICBjb2x1bW5zID0gMTozLAogICAgICAgIG1hcHBpbmcgPSBnZ3Bsb3QyOjphZXMoY29sb3VyID0gcGFkal9jbywgYWxwaGEgPSAwLjUpLCAKICAgICAgICBsb3dlciA9IGxpc3QoY29udGludW91cyA9IHdyYXAoImNvciIsIHNpemUgPSA1KSksIAogICAgICAgIHVwcGVyID0gbGlzdChjb250aW51b3VzID0gInNtb290aCIpLAogICAgICAgIGRpYWcgPSBsaXN0KGNvbnRpbnVvdXMgPSAiZGVuc2l0eURpYWciKSkgICsgc2NhdHRlcl90aGVtZQoKCiMjIFBUZXggMC4wMTUKcGFkal9jbyA8LSBub3JtX01hc3NTcGVjRGF0YSRwYWRqLlBUZXhfMDAxNSA8PSAwLjAxCnBhZGpfY29bd2hpY2gocGFkal9jbyldIDwtICJzaWduaWZpY2FudCIKcGFkal9jb1t3aGljaChwYWRqX2NvPT1GQUxTRSldIDwtICJub24tc2lnbmlmaWNhbnQiCgpmY19wdGV4XzAwMTVfc2NhdHRlciA8LSBnZ3BhaXJzKGNiaW5kKG5vcm1fTWFzc1NwZWNEYXRhWyxGQ1sxMDoxMl1dLCBwYWRqX2NvKSwKICAgICAgICBjb2x1bW5zID0gMTozLAogICAgICAgIG1hcHBpbmcgPSBnZ3Bsb3QyOjphZXMoY29sb3VyID0gcGFkal9jbywgYWxwaGEgPSAwLjUpLCAKICAgICAgICBsb3dlciA9IGxpc3QoY29udGludW91cyA9IHdyYXAoImNvciIsIHNpemUgPSA1KSksIAogICAgICAgIHVwcGVyID0gbGlzdChjb250aW51b3VzID0gInNtb290aCIpLAogICAgICAgIGRpYWcgPSBsaXN0KGNvbnRpbnVvdXMgPSAiZGVuc2l0eURpYWciKSkgICsgc2NhdHRlcl90aGVtZQoKCiMjIFBUZXggMC4xNQpwYWRqX2NvIDwtIG5vcm1fTWFzc1NwZWNEYXRhJHBhZGouUFRleF8wMTUgPD0gMC4wMQpwYWRqX2NvW3doaWNoKHBhZGpfY28pXSA8LSAic2lnbmlmaWNhbnQiCnBhZGpfY29bd2hpY2gocGFkal9jbz09RkFMU0UpXSA8LSAibm9uLXNpZ25pZmljYW50IgoKZmNfcHRleF8wMTVfc2NhdHRlciA8LSBnZ3BhaXJzKGNiaW5kKG5vcm1fTWFzc1NwZWNEYXRhWyxGQ1sxMzoxNV1dLCBwYWRqX2NvKSwKICAgICAgICBjb2x1bW5zID0gMTozLAogICAgICAgIG1hcHBpbmcgPSBnZ3Bsb3QyOjphZXMoY29sb3VyID0gcGFkal9jbywgYWxwaGEgPSAwLjUpLAogICAgICAgIGxvd2VyID0gbGlzdChjb250aW51b3VzID0gd3JhcCgiY29yIiwgc2l6ZSA9IDUpKSwgCiAgICAgICAgdXBwZXIgPSBsaXN0KGNvbnRpbnVvdXMgPSAic21vb3RoIiksCiAgICAgICAgZGlhZyA9IGxpc3QoY29udGludW91cyA9ICJkZW5zaXR5RGlhZyIpKSAgKyBzY2F0dGVyX3RoZW1lCgoKIyMgUFRleCAxLjUKcGFkal9jbyA8LSBub3JtX01hc3NTcGVjRGF0YSRwYWRqLlBUZXhfMTUgPD0gMC4wMQpwYWRqX2NvW3doaWNoKHBhZGpfY28pXSA8LSAic2lnbmlmaWNhbnQiCnBhZGpfY29bd2hpY2gocGFkal9jbz09RkFMU0UpXSA8LSAibm9uLXNpZ25pZmljYW50IgoKZmNfcHRleF8xNV9zY2F0dGVyIDwtIGdncGFpcnMoY2JpbmQobm9ybV9NYXNzU3BlY0RhdGFbLEZDWzE2OjE4XV0sIHBhZGpfY28pLAogICAgICAgIGNvbHVtbnMgPSAxOjMsCiAgICAgICAgbWFwcGluZyA9IGdncGxvdDI6OmFlcyhjb2xvdXIgPSBwYWRqX2NvLCBhbHBoYSA9IDAuNSksIAogICAgICAgIGxvd2VyID0gbGlzdChjb250aW51b3VzID0gd3JhcCgiY29yIiwgc2l6ZSA9IDUpKSwgCiAgICAgICAgdXBwZXIgPSBsaXN0KGNvbnRpbnVvdXMgPSAic21vb3RoIiksCiAgICAgICAgZGlhZyA9IGxpc3QoY29udGludW91cyA9ICJkZW5zaXR5RGlhZyIpKSAgKyBzY2F0dGVyX3RoZW1lCgpgYGAKCmBgYHtyLCBmaWcud2lkdGg9MTIsIGZpZy5oZWlnaHQ9N30KZmNfaW5wdXRfMDAxNV9zY2F0dGVyCmZjX2lucHV0XzAxNV9zY2F0dGVyCmZjX2lucHV0XzE1X3NjYXR0ZXIKCmZjX3B0ZXhfMDAxNV9zY2F0dGVyCmZjX3B0ZXhfMDE1X3NjYXR0ZXIKZmNfcHRleF8xNV9zY2F0dGVyCmBgYAoKIyMjIFZvbGNhbm8gUGxvdHMKCmBgYHtyfQojIyBJbnB1dCAwLjAxNQpwYWRqX2NvIDwtIG5vcm1fTWFzc1NwZWNEYXRhJHBhZGouSW5wdXRfMDAxNSA8PSAwLjAxCnBhZGpfY29bd2hpY2gocGFkal9jbyldIDwtICJzaWduaWZpY2FudCIKcGFkal9jb1t3aGljaChwYWRqX2NvPT1GQUxTRSldIDwtICJub24tc2lnbmlmaWNhbnQiCgpmY19pbnB1dF8wMDE1X3ZvbGNhbm8gPC0gZ2dwbG90KGRhdGE9Y2JpbmQobm9ybV9NYXNzU3BlY0RhdGEsIHBhZGpfY28pLCBhZXMoeD1GQy5JbnB1dF8wMDE1Lm1lYW4sIHk9LWxvZzEwKHBhZGouSW5wdXRfMDAxNSksIGNvbG91ciA9IHBhZGpfY28gKSApICsKICBnZW9tX3BvaW50KGFscGhhPTAuNSwgc2l6ZT0xLjc1KSArCiMgIHhsaW0oYygtNiwgNikpICsgeWxpbShjKDAsIDEwKSkgKwogIHhsYWIoImxvZzIgZm9sZCBjaGFuZ2UiKSArIHlsYWIoIi1sb2cxMCBhZGouIHAtdmFsdWUiKQoKCiMjIElucHV0IDAuMTUKcGFkal9jbyA8LSBub3JtX01hc3NTcGVjRGF0YSRwYWRqLklucHV0XzAxNSA8PSAwLjAxCnBhZGpfY29bd2hpY2gocGFkal9jbyldIDwtICJzaWduaWZpY2FudCIKcGFkal9jb1t3aGljaChwYWRqX2NvPT1GQUxTRSldIDwtICJub24tc2lnbmlmaWNhbnQiCgpmY19pbnB1dF8wMTVfdm9sY2FubyA8LSBnZ3Bsb3QoZGF0YT1jYmluZChub3JtX01hc3NTcGVjRGF0YSwgcGFkal9jbyksIGFlcyh4PUZDLklucHV0XzAxNS5tZWFuLCB5PS1sb2cxMChwYWRqLklucHV0XzAxNSksIGNvbG91ciA9IHBhZGpfY28gKSApICsKICBnZW9tX3BvaW50KGFscGhhPTAuNSwgc2l6ZT0xLjc1KSArCiMgIHhsaW0oYygtNiwgNikpICsgeWxpbShjKDAsIDEwKSkgKwogIHhsYWIoImxvZzIgZm9sZCBjaGFuZ2UiKSArIHlsYWIoIi1sb2cxMCBhZGouIHAtdmFsdWUiKQoKCiMjIElucHV0IDEuNQpwYWRqX2NvIDwtIG5vcm1fTWFzc1NwZWNEYXRhJHBhZGouSW5wdXRfMTUgPD0gMC4wMQpwYWRqX2NvW3doaWNoKHBhZGpfY28pXSA8LSAic2lnbmlmaWNhbnQiCnBhZGpfY29bd2hpY2gocGFkal9jbz09RkFMU0UpXSA8LSAibm9uLXNpZ25pZmljYW50IgoKZmNfaW5wdXRfMTVfdm9sY2FubyA8LSBnZ3Bsb3QoZGF0YT1jYmluZChub3JtX01hc3NTcGVjRGF0YSwgcGFkal9jbyksIGFlcyh4PUZDLklucHV0XzE1Lm1lYW4sIHk9LWxvZzEwKHBhZGouSW5wdXRfMTUpLCBjb2xvdXIgPSBwYWRqX2NvICkgKSArCiAgZ2VvbV9wb2ludChhbHBoYT0wLjUsIHNpemU9MS43NSkgKwojICB4bGltKGMoLTYsIDYpKSArIHlsaW0oYygwLCAxMCkpICsKICB4bGFiKCJsb2cyIGZvbGQgY2hhbmdlIikgKyB5bGFiKCItbG9nMTAgYWRqLiBwLXZhbHVlIikKCiMjIFBUZXggMC4wMTUKcGFkal9jbyA8LSBub3JtX01hc3NTcGVjRGF0YSRwYWRqLlBUZXhfMDAxNSA8PSAwLjAxCnBhZGpfY29bd2hpY2gocGFkal9jbyldIDwtICJzaWduaWZpY2FudCIKcGFkal9jb1t3aGljaChwYWRqX2NvPT1GQUxTRSldIDwtICJub24tc2lnbmlmaWNhbnQiCgpmY19wdGV4XzAwMTVfdm9sY2FubyA8LSBnZ3Bsb3QoZGF0YT1jYmluZChub3JtX01hc3NTcGVjRGF0YSwgcGFkal9jbyksIGFlcyh4PUZDLlBUZXhfMDAxNS5tZWFuLCB5PS1sb2cxMChwYWRqLlBUZXhfMDAxNSksIGNvbG91ciA9IHBhZGpfY28gKSApICsKICBnZW9tX3BvaW50KGFscGhhPTAuNSwgc2l6ZT0xLjc1KSArCiMgIHhsaW0oYygtNiwgNikpICsgeWxpbShjKDAsIDEwKSkgKwogIHhsYWIoImxvZzIgZm9sZCBjaGFuZ2UiKSArIHlsYWIoIi1sb2cxMCBhZGouIHAtdmFsdWUiKQoKCiMjIFBUZXggMC4xNQpwYWRqX2NvIDwtIG5vcm1fTWFzc1NwZWNEYXRhJHBhZGouUFRleF8wMTUgPD0gMC4wMQpwYWRqX2NvW3doaWNoKHBhZGpfY28pXSA8LSAic2lnbmlmaWNhbnQiCnBhZGpfY29bd2hpY2gocGFkal9jbz09RkFMU0UpXSA8LSAibm9uLXNpZ25pZmljYW50IgoKZmNfcHRleF8wMTVfdm9sY2FubyA8LSBnZ3Bsb3QoZGF0YT1jYmluZChub3JtX01hc3NTcGVjRGF0YSwgcGFkal9jbyksIGFlcyh4PUZDLlBUZXhfMDE1Lm1lYW4sIHk9LWxvZzEwKHBhZGouUFRleF8wMTUpLCBjb2xvdXIgPSBwYWRqX2NvICkgKSArCiAgZ2VvbV9wb2ludChhbHBoYT0wLjUsIHNpemU9MS43NSkgKwojICB4bGltKGMoLTYsIDYpKSArIHlsaW0oYygwLCAxMCkpICsKICB4bGFiKCJsb2cyIGZvbGQgY2hhbmdlIikgKyB5bGFiKCItbG9nMTAgYWRqLiBwLXZhbHVlIikKCiMjIFBUZXggMS41CnBhZGpfY28gPC0gbm9ybV9NYXNzU3BlY0RhdGEkcGFkai5QVGV4XzE1IDw9IDAuMDEKcGFkal9jb1t3aGljaChwYWRqX2NvKV0gPC0gInNpZ25pZmljYW50IgpwYWRqX2NvW3doaWNoKHBhZGpfY289PUZBTFNFKV0gPC0gIm5vbi1zaWduaWZpY2FudCIKCmZjX3B0ZXhfMTVfdm9sY2FubyA8LSBnZ3Bsb3QoZGF0YT1jYmluZChub3JtX01hc3NTcGVjRGF0YSwgcGFkal9jbyksIGFlcyh4PUZDLlBUZXhfMTUubWVhbiwgeT0tbG9nMTAocGFkai5QVGV4XzE1KSwgY29sb3VyID0gcGFkal9jbyApICkgKwogIGdlb21fcG9pbnQoYWxwaGE9MC41LCBzaXplPTEuNzUpICsKIyAgeGxpbShjKC02LCA2KSkgKyB5bGltKGMoMCwgMTApKSArCiAgeGxhYigibG9nMiBmb2xkIGNoYW5nZSIpICsgeWxhYigiLWxvZzEwIGFkai4gcC12YWx1ZSIpCgpgYGAKCmBgYHtyLCBmaWcud2lkdGg9MTIsIGZpZy5oZWlnaHQ9N30KZmNfaW5wdXRfMDAxNV92b2xjYW5vCmZjX2lucHV0XzAxNV92b2xjYW5vCmZjX2lucHV0XzE1X3ZvbGNhbm8KCmZjX3B0ZXhfMDAxNV92b2xjYW5vCmZjX3B0ZXhfMDE1X3ZvbGNhbm8KZmNfcHRleF8xNV92b2xjYW5vCmBgYAoKVGlkeSB1cDoKCmBgYHtyfQpybShsaXN0ID0gbHMocGF0dGVybiA9ICJfc2NhdHRlciIpKQpybShsaXN0ID0gbHMocGF0dGVybiA9ICJfdm9sY2FubyIpKQpybShwYWRqX2NvKQpgYGAKCiMgTWF0Y2hpbmcKClRvIGFzc2VzcyBwZXJmb3JtYW5jZSBvZiBQVGV4IHRvIGZpbmQgUkJQcywgdGhlIHNpZ25pZmljYW50bHkgZW5yaWNoZWQgcHJvdGVpbnMgYXJlIGNvbXBhcmVkIHRvIG90aGVyIHN0dWRpZXMuClRoZSBmb2xsb3dpbmcgc3R1ZGllcyBhcmUgdGFrZW4gaW50byBhY2NvdW50OgogIC0gR2Vyc3RiZXJnZXIgcmV2aWV3IDIwMTQKICAtIExhbmR0aGFsZXIgSEVLMjkzIGludGVyYWN0b21lIGNhcHR1cmUKICAtIFByZWlzcwogIC0gU09OQVIgKGFubm90YXRlZCBhbmQgcHJlZGljdGVkKQogIC0gSHVic3RlbmJlcmdlciBQLUJvZHkgcHJvdGVpbnMKICAtIFJJQ0sgKGhpZ2gsIGxvdyBhbmQgdW5pcXVlKQogIC0gQ0FSSUMKCmBgYHtyIGRhdGEgc2V0IGxvYWRpbmd9CiMjIEdlcnN0YmVyZ2VyIHJldmlldyAoMjAxNCkgUkJQcwoKZ2UgPC0gcmVhZC5jc3YoImlucHV0ZGF0YS9SQlAgdGFibGUtVGFibGUgMS5jc3YiKQoKIyMgR2Vyc3RiZXJnZXIgcmV2aWV3ICgyMDE0KSBURnMKCnRmIDwtIHJlYWQuY3N2KCJpbnB1dGRhdGEvaHVtYW4gVEZzLVRhYmxlIDEuY3N2IikKCiMjIEh1bWFuIG1STkEgaW50ZXJhY3RvbWUgY2FwdHVyZSBkYXRhOgoKaWMgPC0gcmVhZC5jc3YoImlucHV0ZGF0YS8yMDE1LTA2LTA5X2h1bWFuX2ludGVyYWN0b21lcy5jc3YiLG5hLnN0cmluZ3M9YygiIiwgIk5BIikgKQoKIyMgUHJlaXNzCgpwcmVpc3NfZ24gPC0gcmVhZC5jc3YoImlucHV0ZGF0YS9wcmVpc3NfZ2VuZW5hbWVzLmNzdiIsIGhlYWRlcj1ULCBzZXA9Ilx0IikKCiMjIGFubm90YXRlZCBSQlBzIGZyb20gU09OQVIgcGFwZXIgKEJyYW5uYW4gZXQgYWwuKQoKc29uYXJfYWxsIDwtIHJlYWQuY3N2KCJpbnB1dGRhdGEvQnJhbm5hbl9hbm5vdGF0ZWQudHh0IiwgaGVhZGVyID0gRkFMU0UpCgojIyBQcmVkaWN0ZWQgU09OQVIgcHJvdGVpbnMgKHdpdGggc2NvcmUgPiAwLjc5LCBzZWUgQnJhbm5hbiBldCBhbC4gTW9sIENlbGwgMjAxNikKCnNvbmFyXzA3OSA8LSByZWFkLmNzdigiaW5wdXRkYXRhL0JyYW5uYW5fU09OQVJfMC43OS50eHQiLCBoZWFkZXIgPSBGQUxTRSkKCiMjIEh1YnN0ZW5iZXJnZXIgUC1Cb2R5IFByb3RlaW5zCgpodWJzdCA8LSByZWFkLmNzdigiaW5wdXRkYXRhL2h1YnN0ZW5iZXJnZXIuY3N2IiwgaGVhZGVyID0gVFJVRSwgc2VwID0gIlx0IikKCiMjIFJJQ0sKCnJpY2tfaGlnaCA8LSByZWFkLmNzdigiaW5wdXRkYXRhL1JJQ0tfaGlnaF9jb25mLmNzdiIsIHNlcCA9ICJcdCIsIHN0cmluZ3NBc0ZhY3RvcnMgPSBGKQpyaWNrX2xvdyA8LSByZWFkLmNzdigiaW5wdXRkYXRhL1JJQ0tfbG93X2NvbmYuY3N2Iiwgc2VwID0gIlx0Iiwgc3RyaW5nc0FzRmFjdG9ycyA9IEYpCnJpY2tfdW5pcXVlIDwtIHJlYWQuY3N2KCJpbnB1dGRhdGEvUklDS191bmlxdWUuY3N2Iiwgc2VwPSJcdCIsIHN0cmluZ3NBc0ZhY3RvcnMgPSBGKQoKIyMgQ0FSSUMKCmNhcmljIDwtIHJlYWQuY3N2KCJpbnB1dGRhdGEvY2FyaWMuY3N2Iiwgc2VwID0gIlx0Iiwgc3RyaW5nc0FzRmFjdG9ycyA9IEYpCmBgYAoKIyMgTWF0Y2hpbmcgdGFibGUKCkEgbWF0Y2hpbmcgdGFibGUgaXMgY3JlYXRlZCB0byBwZXJmb3JtIHRoZSBtYXRjaGluZyBhbmQgc3RvcmUgdGhlIHJlc3VsdHMuIEl0IGNvbnRhaW5zIHRoZSBmb2xsb3dpbmcgaWRlbnRpZmllciBjb2x1bW5zOgogIC0gTWFqb3JpdHkucHJvdGVpbi5JRHM6IHRoZSBNYWpvcml0eS5wcm90ZWluLklEcyBvZiB0aGUgc2lnbmlmaWNhbnRseSBlbnJpY2hlZCBQVGV4IGhpdHMKICAtIE1QSURzTm9Jc286IE1ham9yaXR5LnByb3RlaW4uSURzIHdpdGggaXNvZm9ybXMgcmVzb2x2ZWQgdG8gY2Fub25pY2FsIGZvcm0KICAtIGdlbmVfbmFtZTogdGhlIGdlbmUgbmFtZXMgb2YgdGhlIHNpZ25pZmljYW50bHkgZW5yaWNoZWQgUFRleCBoaXRzCgpgYGB7cn0KbWF0Y2hpbmdfdGFibGUgPC0gZGF0YS5mcmFtZShNYWpvcml0eS5wcm90ZWluLklEcyA9IGFzLmNoYXJhY3RlcihzaWdfbm9ybV9NYXNzU3BlY0RhdGEkTWFqb3JpdHkucHJvdGVpbi5JRHMpKQptYXRjaGluZ190YWJsZSRNYWpvcml0eS5wcm90ZWluLklEcyA8LSBhcHBseShYID0gbWF0Y2hpbmdfdGFibGUsIE1BUkdJTiA9IDEsIEZVTiA9IGZ1bmN0aW9uKHgpdW5saXN0KHN0cnNwbGl0KGFzLmNoYXJhY3Rlcih4KSwgc3BsaXQ9IjsiKSkpCm1hdGNoaW5nX3RhYmxlJE1QSURzTm9Jc28gPC0gbGFwcGx5KHN0cnNwbGl0KGdzdWIoIi1bMC05XXsxLDJ9IiwiIixzaWdfbm9ybV9NYXNzU3BlY0RhdGEkTWFqb3JpdHkucHJvdGVpbi5JRHMpLHNwbGl0PSI7IiksIGZ1bmN0aW9uKHgpdW5pcXVlKHgpKQptYXRjaGluZ190YWJsZSRnZW5lX25hbWUgPC0gc2lnX25vcm1fTWFzc1NwZWNEYXRhJEdlbmUubmFtZXMKYGBgCgpUaGUgY2Fub25pY2FsIE1QSURzIHdlcmUgbWF0Y2hlZCB3aXRoIGFsbCByZXZpZXdlZCBodW1hbiBVbmlQcm90IChTd2lzc1Byb3QpIGVudHJpZXMuCgpgYGB7cn0KYWxsX3Jldl91bmlwcm90IDwtIHJlYWQudGFibGUoImlucHV0ZGF0YS9odW1hbl9wcm90ZW9tZV9yZXZpZXdlZF8yOTExMTgubGlzdCIsIGhlYWRlcj1GLCBjb2wubmFtZXMgPSAidW5pcHJvdCIpCgpNUElEc05vSXNvX3Jldl9zdGF0IDwtIGFwcGx5KG1hdGNoaW5nX3RhYmxlLCAxLCBmdW5jdGlvbih4KSB1bmxpc3QoeCRNUElEc05vSXNvKSAlaW4lIGFsbF9yZXZfdW5pcHJvdCR1bmlwcm90KQpNUElEc05vSXNvLnJldiA8LSBtYXBwbHkoeD1tYXRjaGluZ190YWJsZSRNUElEc05vSXNvLCB5PU1QSURzTm9Jc29fcmV2X3N0YXQsIGZ1bmN0aW9uKHgseSl1bmxpc3QoeFt1bmxpc3QoeSldKSkKTVBJRHNOb0lzby5yZXZbdW5saXN0KGxhcHBseShNUElEc05vSXNvLnJldiwgZnVuY3Rpb24oeClsZW5ndGgoeCk9PTApKV0gPC0gTkEKc3VtKGlzLm5hKE1QSURzTm9Jc28ucmV2KSkKCm1hdGNoaW5nX3RhYmxlJE1QSURyIDwtIE1QSURzTm9Jc28ucmV2CmBgYAoKRm9yIDE2IG1ham9yaXR5IHByb3RlaW4gSUQgZ3JvdXBzIG5vIFN3aXNzUHJvdCBlbnRyeSBjb3VsZCBiZSBmb3VuZC4KCmBgYHtyfQpkYXRhdGFibGUobWF0Y2hpbmdfdGFibGVbd2hpY2goTVBJRHNOb0lzby5yZXYlaW4lIk5BIiksXSkKYGBgCgpUaG9zZSAxNiBoaXRzIHdlcmUgc3ViamVjdGVkIHRvIG1hbnVhbCBjdXJhdGlvbiwgbWVhbmluZyB0aGUgYWNjZXNzaW9uIG51bWJlcnMgYW5kIGdlbmUgbmFtZXMgd2VyZSB1c2VkIGZvciBhIFVuaVByb3QgcXVlcnkuIElmIGFuIHVuYW1iaWd1b3VzIGhpdCB3YXMgZm91bmQgaXQgd2FzIGNvcnJlY3RlZC4gVGhlIGZvbGxvd2luZyB3ZXJlIGNvcnJlY3RlZDoKCmBgYHtyfQptYXRjaGluZ190YWJsZSRNUElEcls3NF0gPC0gIlA0MzI0MyIKbWF0Y2hpbmdfdGFibGUkTVBJRHJbODNdIDwtICJPNzUxMjIiCm1hdGNoaW5nX3RhYmxlJE1QSURyWzkzXSA8LSAiUTlVQlgzIgptYXRjaGluZ190YWJsZSRNUElEclsxMzRdIDwtICJPOTU4MTkiCm1hdGNoaW5nX3RhYmxlJE1QSURyWzIyNl0gPC0gIlE5VVBOMyIKbWF0Y2hpbmdfdGFibGUkTVBJRHJbMjY0XSA8LSAiUTk2TVU3IgptYXRjaGluZ190YWJsZSRNUElEclsyNjhdIDwtICJQMzY3NzYiCm1hdGNoaW5nX3RhYmxlJE1QSURyWzY3NF0gPC0gIlAwRE1WOCIKbWF0Y2hpbmdfdGFibGUkTVBJRHJbMjkzN10gPC0gIlAwRFBCNiIKbWF0Y2hpbmdfdGFibGUkTVBJRHJbMzAzNF0gPC0gIlAxNzk4MCIKbWF0Y2hpbmdfdGFibGUkTVBJRHJbMzAzNV0gPC0gIlE5VUtWMyIKYGBgCgpBZnRlciBtYW51YWwgY29ycmVjdGlvbiwgdGhlIGZvbGxvd2luZyB3ZXJlIHN0aWxsIG5vdCBpZGVudGlmaWFibGUuCgpgYGB7cn0KZGF0YXRhYmxlKG1hdGNoaW5nX3RhYmxlW3doaWNoKG1hdGNoaW5nX3RhYmxlJE1QSURyJWluJSJOQSIpLF0pCmBgYAoKVGhlc2UgaGl0cyB3ZXJlIGV4Y2x1ZGVkIGZyb20gZnVydGhlciBhbmFseXNlcy4KCmBgYHtyfQptYXRjaGluZ190YWJsZSA8LSBtYXRjaGluZ190YWJsZVstYygxNjIsMTY5LDE3OCw4OTYsMzAzNyksXQpkYXRhdGFibGUobWF0Y2hpbmdfdGFibGVbd2hpY2gobWF0Y2hpbmdfdGFibGUkTVBJRHIlaW4lIk5BIiksXSkKYGBgCgpXQVJOSU5HOmAyMzUgUTEzNDA0O0E1UExMNyBVQkUyVjE7VE1FTTE4OSAgVDtGYCBzdGF5cyEgT3RoZXJzIG1pZ2h0LCB0b28hCgojIyMgTWF0Y2hpbmcgd2l0aCBvdGhlciBkYXRhc2V0cwoKYGBge3J9CiMjIEdlcnN0YmVyZ2VyIFJCUHMKCm1hdGNoaW5nX3RhYmxlJEdlcnN0YmVyZ2VyLlJCUCA8LSBhcHBseShtYXRjaGluZ190YWJsZSwxLGZ1bmN0aW9uKHgpIGFueShnZSRnZW5lLm5hbWUgJWluJSB1bmxpc3Qoc3Ryc3BsaXQoYXMuY2hhcmFjdGVyKHgkZ2VuZV9uYW1lKSxzcGxpdD0iOyIpKSkpCgojIyBHZXJzdGJlcmdlciBURnMgKG5lZ2F0aXZlIGNvbnRyb2wpCgptYXRjaGluZ190YWJsZSRHZXJzdGJlcmdlci5URiA8LSBhcHBseShtYXRjaGluZ190YWJsZSwxLGZ1bmN0aW9uKHgpIGFueSh0ZiRnZW5lLm5hbWUgJWluJSB1bmxpc3Qoc3Ryc3BsaXQoYXMuY2hhcmFjdGVyKHgkZ2VuZV9uYW1lKSxzcGxpdD0iOyIpKSkpCgojIyBMYW5kdGhhbGVyIEhFSzI5MyBpbnRlcmFjdG9tZSBjYXB0dXJlIGRhdGEKCmhla21SQlAgPC0gc3Vic2V0KGljLCBpYyRIRUsyOTMgPT0gIm1STkEgaW50ZXJhY3RvbWUiKQptYXRjaGluZ190YWJsZSRMYW5kdGhhbGVyLm1SQlAgPC0gYXBwbHkobWF0Y2hpbmdfdGFibGUsIDEsIGZ1bmN0aW9uKHgpIGFueShoZWttUkJQJFVuaXByb3QuZW50cnkgJWluJSB1bmxpc3QoeCRNUElEcikpKQoKIyMgUHJlaXNzCgptYXRjaGluZ190YWJsZSRQcmVpc3MgPC0gYXBwbHkobWF0Y2hpbmdfdGFibGUsIDEsIGZ1bmN0aW9uKHgpIGFueShwcmVpc3NfZ24kZ2VuZS5uYW1lICVpbiUgdW5saXN0KHN0cnNwbGl0KGFzLmNoYXJhY3Rlcih4JGdlbmVfbmFtZSksc3BsaXQ9IjsiKSkpKQoKIyMgQW5ub3RhdGVkIFJCUHMgKGZyb20gdGhlIFNPTkFSIHBhcGVyKQoKbWF0Y2hpbmdfdGFibGUkQnJhbm5hbi5hbm5vdGF0ZWQgPC0gYXBwbHkobWF0Y2hpbmdfdGFibGUsIDEsIGZ1bmN0aW9uKHgpIGFueShzb25hcl9hbGwkVjEgJWluJSB1bmxpc3Qoc3Ryc3BsaXQoYXMuY2hhcmFjdGVyKHgkZ2VuZV9uYW1lKSxzcGxpdD0iOyIpKSkpCgojIyBTT05BUi1wcmVkaWN0ZWQgUkJQcwoKbWF0Y2hpbmdfdGFibGUkQnJhbm5hbi5wcmVkaWN0ZWQgPC0gYXBwbHkobWF0Y2hpbmdfdGFibGUsIDEsIGZ1bmN0aW9uKHgpIGFueShzb25hcl8wNzkkVjEgJWluJSB1bmxpc3Qoc3Ryc3BsaXQoYXMuY2hhcmFjdGVyKHgkZ2VuZV9uYW1lKSxzcGxpdD0iOyIpKSkpCgojIyBIdWJzdGVuYmVyZ2VyIFAtQm9keSBwcm90ZWlucwoKbWF0Y2hpbmdfdGFibGUkSHVic3RlbmJlcmdlci5QQlAgPC0gYXBwbHkobWF0Y2hpbmdfdGFibGUsIDEsIGZ1bmN0aW9uKHgpIGFueShodWJzdCRnZW5lLm5hbWUgJWluJSB1bmxpc3Qoc3Ryc3BsaXQoYXMuY2hhcmFjdGVyKHgkZ2VuZV9uYW1lKSxzcGxpdD0iOyIpKSkpCgojIyBSSUNLIGRhdGEKCm1hdGNoaW5nX3RhYmxlJFJpY2suaGlnaCA8LSBhcHBseShtYXRjaGluZ190YWJsZSwgMSwgZnVuY3Rpb24oeCkgYW55KHJpY2tfaGlnaCRQcm90ZWluX0lEICVpbiUgdW5saXN0KHgkTVBJRHIpKSkKbWF0Y2hpbmdfdGFibGUkUmljay5sb3cgPC0gYXBwbHkobWF0Y2hpbmdfdGFibGUsIDEsIGZ1bmN0aW9uKHgpIGFueShyaWNrX2xvdyRQcm90ZWluX0lEICVpbiUgdW5saXN0KHgkTVBJRHIpKSkKbWF0Y2hpbmdfdGFibGUkUmljay51bmlxdWUgPC0gYXBwbHkobWF0Y2hpbmdfdGFibGUsIDEsIGZ1bmN0aW9uKHgpIGFueShyaWNrX3VuaXF1ZSRQcm90ZWluX0lEICVpbiUgdW5saXN0KHgkTVBJRHIpKSkKCiMjIENBUklDIFJCUHMKCm1hdGNoaW5nX3RhYmxlJENhcmljIDwtIGFwcGx5KG1hdGNoaW5nX3RhYmxlLCAxLCBmdW5jdGlvbih4KSBhbnkoY2FyaWMkVW5pUHJvdC5hY2Nlc3Npb24gJWluJSB1bmxpc3QoeCRNUElEcikpKQpgYGAKCk5vdGUgdGhhdCBtYXRjaGluZyB2aWEgc3RyaW5nc3BsaXR0ZWQgZ2VuZSBuYW1lcyBjYW4gcHJvZHVjZSBtdWx0aXBsZSBoaXRzIGluIHJlZmVyZW5jZSBmb3IgYSBzaW5nbGUgUFRleCBoaXQuCgojIyBNYXRjaGluZyBkZgoKYGBge3J9CiMgaW5QVGV4LCBub3QgaW5QVGV4CgptYXRjaGluZ19kZiA8LSBkYXRhLmZyYW1lKAogIG1hdGNoID0gYygieWVzIiwgIm5vIiksCiAgR2Vyc3RiZXJnZXIuUkJQcyA9IGMocm91bmQoc3VtKG1hdGNoaW5nX3RhYmxlJEdlcnN0YmVyZ2VyLlJCUCkvbnJvdyhnZSksNCksIHJvdW5kKChucm93KGdlKSAtIHN1bShtYXRjaGluZ190YWJsZSRHZXJzdGJlcmdlci5SQlApKS9ucm93KGdlKSw0KSksCiAgR2Vyc3RiZXJnZXIuVEZzID1jKHJvdW5kKHN1bShtYXRjaGluZ190YWJsZSRHZXJzdGJlcmdlci5URikvbnJvdyh0ZiksNCksIHJvdW5kKChucm93KHRmKSAtIHN1bShtYXRjaGluZ190YWJsZSRHZXJzdGJlcmdlci5URikpL25yb3codGYpLDQpKSwKICBMYW5kdGhhbGVyID0gYyhyb3VuZChzdW0obWF0Y2hpbmdfdGFibGUkTGFuZHRoYWxlci5tUkJQKS9ucm93KGhla21SQlApLDQpLCByb3VuZCgobnJvdyhoZWttUkJQKSAtIHN1bShtYXRjaGluZ190YWJsZSRMYW5kdGhhbGVyLm1SQlApKS9ucm93KGhla21SQlApLDQpKSwKICBQcmVpc3MgPSBjKHJvdW5kKHN1bShtYXRjaGluZ190YWJsZSRQcmVpc3MpL25yb3cocHJlaXNzX2duKSw0KSwgcm91bmQoKG5yb3cocHJlaXNzX2duKS1zdW0obWF0Y2hpbmdfdGFibGUkUHJlaXNzKSkvbnJvdyhwcmVpc3NfZ24pLDQpKSwKICBCcmFubmFuLmFubm90YXRlZCA9IGMocm91bmQoc3VtKG1hdGNoaW5nX3RhYmxlJEJyYW5uYW4uYW5ub3RhdGVkKS9ucm93KHNvbmFyX2FsbCksNCksIHJvdW5kKChucm93KHNvbmFyX2FsbCkgLSBzdW0obWF0Y2hpbmdfdGFibGUkQnJhbm5hbi5hbm5vdGF0ZWQpKS9ucm93KHNvbmFyX2FsbCksNCkpLAogIEJyYW5uYW4ucHJlZGljdGVkID0gYyhyb3VuZChzdW0obWF0Y2hpbmdfdGFibGUkQnJhbm5hbi5wcmVkaWN0ZWQpL25yb3coc29uYXJfMDc5KSw0KSwgcm91bmQoKG5yb3coc29uYXJfMDc5KSAtIHN1bShtYXRjaGluZ190YWJsZSRCcmFubmFuLnByZWRpY3RlZCkpL25yb3coc29uYXJfMDc5KSw0KSksCiAgSHVic3RlbmJlcmdlci5QQlAgPSBjKHJvdW5kKHN1bShtYXRjaGluZ190YWJsZSRIdWJzdGVuYmVyZ2VyLlBCUCkvbnJvdyhodWJzdCksNCksIHJvdW5kKChucm93KGh1YnN0KSAtIHN1bShtYXRjaGluZ190YWJsZSRIdWJzdGVuYmVyZ2VyLlBCUCkpL25yb3coaHVic3QpLDQpKSwKICBSaWNrLmhpZ2ggPSBjKHJvdW5kKHN1bShtYXRjaGluZ190YWJsZSRSaWNrLmhpZ2gpL25yb3cocmlja19oaWdoKSw0KSwgcm91bmQoKG5yb3cocmlja19oaWdoKSAtIHN1bShtYXRjaGluZ190YWJsZSRSaWNrLmhpZ2gpKS9ucm93KHJpY2tfaGlnaCksNCkpLAogIFJpY2subG93ID0gYyhyb3VuZChzdW0obWF0Y2hpbmdfdGFibGUkUmljay5sb3cpL25yb3cocmlja19sb3cpLDQpLCByb3VuZCgobnJvdyhyaWNrX2xvdykgLSBzdW0obWF0Y2hpbmdfdGFibGUkUmljay5sb3cpKS9ucm93KHJpY2tfbG93KSw0KSksCiAgUmljay51bmlxdWUgPSBjKHJvdW5kKHN1bShtYXRjaGluZ190YWJsZSRSaWNrLnVuaXF1ZSkvbnJvdyhyaWNrX3VuaXF1ZSksNCksIHJvdW5kKChucm93KHJpY2tfdW5pcXVlKSAtIHN1bShtYXRjaGluZ190YWJsZSRSaWNrLnVuaXF1ZSkpL25yb3cocmlja191bmlxdWUpLDQpKSwKICBDYXJpYyA9IGMocm91bmQoc3VtKG1hdGNoaW5nX3RhYmxlJENhcmljKS9ucm93KGNhcmljKSw0KSwgcm91bmQoKG5yb3coY2FyaWMpIC0gc3VtKG1hdGNoaW5nX3RhYmxlJENhcmljKSkvbnJvdyhjYXJpYyksNCkpCikKCmBgYAoKYGBge3J9CmdncGxvdChtZWx0KG1hdGNoaW5nX2RmLCBpZC52YXJzID0gIm1hdGNoIiksIGFlcyh4PXZhcmlhYmxlLCB5PXZhbHVlLCBmaWxsPW1hdGNoKSkgKwogIGdlb21fYmFyKHN0YXQgPSAiaWRlbnRpdHkiLCBhbHBoYT0uNikgKwogIHlsYWIoImZyYWN0aW9uIikgKyB4bGFiKCIiKSArCiAgdGhlbWUoYXhpcy50ZXh0LnggPSBlbGVtZW50X3RleHQoYW5nbGUgPSA0NSwgaGp1c3QgPSAxKSkKYGBgCgojIEFubm90YXRpb25zCgpUaGUgc2lnbmlmaWNhbnRseSBlbnJpY2hlZCBoaXRzIGFyZSBhbm5vdGF0ZWQgd2l0aAotIGlzb2VsZWN0cmljIHBvaW50Ci0gbW9sZWN1bGFyIHdlaWdodAotIGh5ZHJvcGhvYmljaXR5Ci0gKFJCRCkKLSAoR08pCgpGb3IgdGhhdCBhIGxpc3Qgb2YgVW5pUHJvdCBhY2Nlc3Npb24gbnVtYmVycyBpcyBuZWVkZWQuIEZvciB0aGF0IHRoZSBgTVBJRHJgIG9mIHRoZSBtYXRjaGluZyB0YWJsZSBpcyB1c2VkLCB3aGlsZSBmb3IgZW50cmllcyB3aXRoIG11bHRpcGxlIGFjY2Vzc2lvbiBudW1iZXJzLCBvbmx5IHRoZSBmaXJzdCBpcyB1c2VkLgoKYGBge3J9CmFjY2Vzc2lvbnMgPC0gdW5saXN0KGxhcHBseShYPW1hdGNoaW5nX3RhYmxlJE1QSURyLCBGVU4gPSBmdW5jdGlvbih4KSBzdHJzcGxpdChhcy5jaGFyYWN0ZXIoeCksIHNwbGl0ID0gIjsiKVtbMV1dWzFdKSkKCndyaXRlLnRhYmxlKGFjY2Vzc2lvbnMsICJhY2Nlc3Npb25zLnRzdiIsIHJvdy5uYW1lcyA9IEYsIGNvbC5uYW1lcyA9IEYpCmBgYAoKIyMgSXNvZWxlY3RyaWMgUG9pbnQsIE1vbGVjdWxhciBXZWlnaHQsIFNlcXVlbmNlcyBhbmQgaHlkcm9waG9iaWNpdHkKCkZvciBjb21wYXJpc29uIG9mIHRoZSBNUyBkYXRhIHdpdGggdGhlIGh1bWFuIHByb3Rlb21lIGEgZGF0YXNldCBmcm9tIGBpc29lbGVjdHJpY3BvaW50ZGIub3JnYCB3YXMgcmV0cmlldmVkIChpbmNsdWRpbmcgaXNvZWxlY3RyaWMgcG9pbnRzLCBtb2xlY3VsYXIgd2VpZ2h0cyBhbmQgc2VxdWVuY2VzKSAoYXMgb2YgMjkuMTEuMTgpOgpgYGB7cn0KaXNvZWxlY3RyaWNwb2ludGRiIDwtIHJlYWQuY3N2KCJpbnB1dGRhdGEvVVAwMDAwMDU2NDBfOTYwNl9hbGwuZmFzdGEucEkuY3N2IiwgaGVhZGVyPVRSVUUsIHNlcD0iLCIpCmBgYAoKYGBge3J9CklQREJfbWF0Y2hpbmcgPC0gZGF0YS5mcmFtZSgKICBVbmlQcm90ID0gYWNjZXNzaW9ucywKICBTZXF1ZW5jZSA9IHJlcChOQSxsZW5ndGgoYWNjZXNzaW9ucykpLAogIE1vbFdlaWdodCA9IHJlcChOQSxsZW5ndGgoYWNjZXNzaW9ucykpLAogIElQQ19wcm90ZWluID0gcmVwKE5BLGxlbmd0aChhY2Nlc3Npb25zKSksCiAgSHlkcm9waG9iaWNpdHkgPSByZXAoTkEsbGVuZ3RoKGFjY2Vzc2lvbnMpKQopCmBgYAoKYGBge3J9CmZvcihpIGluIHNlcShucm93KElQREJfbWF0Y2hpbmcpKSl7CiAgdHJ5KElQREJfbWF0Y2hpbmckU2VxdWVuY2VbaV0gPC0gYXMuY2hhcmFjdGVyKGlzb2VsZWN0cmljcG9pbnRkYiRzZXF1ZW5jZVtncmVwKHBhdHRlcm4gPSBwYXN0ZSgifCIsSVBEQl9tYXRjaGluZyRVbmlQcm90W2ldLCJ8Iiwgc2VwPSIiKSwgeCA9IGlzb2VsZWN0cmljcG9pbnRkYiRoZWFkZXIsIGZpeGVkID0gVFJVRSldKSkKICB0cnkoSVBEQl9tYXRjaGluZyRNb2xXZWlnaHRbaV0gPC0gaXNvZWxlY3RyaWNwb2ludGRiJG1vbGVjdWxhcl93ZWlnaHRbZ3JlcChwYXR0ZXJuID0gcGFzdGUoInwiLElQREJfbWF0Y2hpbmckVW5pUHJvdFtpXSwifCIsIHNlcD0iIiksIHggPSBpc29lbGVjdHJpY3BvaW50ZGIkaGVhZGVyLCBmaXhlZCA9IFRSVUUpXSkKICB0cnkoSVBEQl9tYXRjaGluZyRJUENfcHJvdGVpbltpXSA8LSBpc29lbGVjdHJpY3BvaW50ZGIkSVBDX3Byb3RlaW5bZ3JlcChwYXR0ZXJuID0gcGFzdGUoInwiLElQREJfbWF0Y2hpbmckVW5pUHJvdFtpXSwifCIsIHNlcD0iIiksIHggPSBpc29lbGVjdHJpY3BvaW50ZGIkaGVhZGVyLCBmaXhlZCA9IFRSVUUpXSkKfQpgYGAKCk5vdCBmb3VuZDogUDBEUEI2IHdoaWNoIGlzID5zcHxROVkyUzB8UlBBQzJfSFVNQU4gRE5BLWRpcmVjdGVkIFJOQSBwb2x5bWVyYXNlcyBJIGFuZCBJSUkgc3VidW5pdCBSUEFDMiBPUz1Ib21vIHNhcGllbnMgR049UE9MUjFEIFBFPTEgU1Y9MQoKYGBge3J9CmZvcihpIGluIHNlcShucm93KElQREJfbWF0Y2hpbmcpKSl7CiAgdHJ5KElQREJfbWF0Y2hpbmckSHlkcm9waG9iaWNpdHlbaV0gPC0gYWFDb21wKElQREJfbWF0Y2hpbmckU2VxdWVuY2VbaV0pW1sxXV1bIk5vblBvbGFyIiwiTW9sZSUiXSkKfQpgYGAKCmBhbGxfcmV2X3VuaXByb3RgIHdlcmUgbWF0Y2hlZCB3aXRoIHRoZSBpc29lbGVjdHJpY3BvaW50ZGIuIChOb3RlIHRoYXQgdGhlIGZvbGxvd2luZyBjaHVuayBydW5zIHZlcnkgbG9uZywgYmVjYXVzZSBvZiB0aGUgbG93IHNwZWVkIG9mIGZvciBsb29wcykKCmBgYHtyfQpJUERCX21hdGNoaW5nX3Byb3Rlb21lIDwtIGRhdGEuZnJhbWUoCiAgVW5pUHJvdCA9IGFzLmNoYXJhY3RlcihhbGxfcmV2X3VuaXByb3QkdW5pcHJvdCksCiAgU2VxdWVuY2UgPSByZXAoTkEsbGVuZ3RoKGFsbF9yZXZfdW5pcHJvdCkpLAogIE1vbFdlaWdodCA9IHJlcChOQSxsZW5ndGgoYWxsX3Jldl91bmlwcm90KSksCiAgSVBDX3Byb3RlaW4gPSByZXAoTkEsbGVuZ3RoKGFsbF9yZXZfdW5pcHJvdCkpLAogIEh5ZHJvcGhvYmljaXR5ID0gcmVwKE5BLGxlbmd0aChhbGxfcmV2X3VuaXByb3QpKQopCgpmb3IoaSBpbiBzZXEobnJvdyhJUERCX21hdGNoaW5nX3Byb3Rlb21lKSkpewogIHRyeShJUERCX21hdGNoaW5nX3Byb3Rlb21lJFNlcXVlbmNlW2ldIDwtIGFzLmNoYXJhY3Rlcihpc29lbGVjdHJpY3BvaW50ZGIkc2VxdWVuY2VbZ3JlcChwYXR0ZXJuID0gcGFzdGUoInwiLElQREJfbWF0Y2hpbmdfcHJvdGVvbWUkVW5pUHJvdFtpXSwifCIsIHNlcD0iIiksIHggPSBpc29lbGVjdHJpY3BvaW50ZGIkaGVhZGVyLCBmaXhlZCA9IFRSVUUpXSkpCiAgdHJ5KElQREJfbWF0Y2hpbmdfcHJvdGVvbWUkTW9sV2VpZ2h0W2ldIDwtIGlzb2VsZWN0cmljcG9pbnRkYiRtb2xlY3VsYXJfd2VpZ2h0W2dyZXAocGF0dGVybiA9IHBhc3RlKCJ8IixJUERCX21hdGNoaW5nX3Byb3Rlb21lJFVuaVByb3RbaV0sInwiLCBzZXA9IiIpLCB4ID0gaXNvZWxlY3RyaWNwb2ludGRiJGhlYWRlciwgZml4ZWQgPSBUUlVFKV0pCiAgdHJ5KElQREJfbWF0Y2hpbmdfcHJvdGVvbWUkSVBDX3Byb3RlaW5baV0gPC0gaXNvZWxlY3RyaWNwb2ludGRiJElQQ19wcm90ZWluW2dyZXAocGF0dGVybiA9IHBhc3RlKCJ8IixJUERCX21hdGNoaW5nX3Byb3Rlb21lJFVuaVByb3RbaV0sInwiLCBzZXA9IiIpLCB4ID0gaXNvZWxlY3RyaWNwb2ludGRiJGhlYWRlciwgZml4ZWQgPSBUUlVFKV0pCn0KCmZvcihpIGluIHNlcShucm93KElQREJfbWF0Y2hpbmdfcHJvdGVvbWUpKSl7CiAgdHJ5KElQREJfbWF0Y2hpbmdfcHJvdGVvbWUkSHlkcm9waG9iaWNpdHlbaV0gPC0gYWFDb21wKElQREJfbWF0Y2hpbmdfcHJvdGVvbWUkU2VxdWVuY2VbaV0pW1sxXV1bIk5vblBvbGFyIiwiTW9sZSUiXSkKfQpgYGAKClNvbWUgb2YgdGhlIGFjY2Vzc2lvbiBudW1iZXJzIHdlcmUgbm90IGluY2x1ZGVkIGluIHRoZSBpc29lbGVjdHJpY3BvaW50ZGIuCgpgYGB7cn0Kc3VtKHNhcHBseShJUERCX21hdGNoaW5nX3Byb3Rlb21lJFNlcXVlbmNlLCBmdW5jdGlvbih4KWlzLm5hKHgpKSkKc3VtKHNhcHBseShJUERCX21hdGNoaW5nX3Byb3Rlb21lJE1vbFdlaWdodCwgZnVuY3Rpb24oeClpcy5uYSh4KSkpCnN1bShzYXBwbHkoSVBEQl9tYXRjaGluZ19wcm90ZW9tZSRJUENfcHJvdGVpbiwgZnVuY3Rpb24oeClpcy5uYSh4KSkpCnN1bShzYXBwbHkoSVBEQl9tYXRjaGluZ19wcm90ZW9tZSRIeWRyb3Bob2JpY2l0eSwgZnVuY3Rpb24oeClpcy5uYW4oeCkpKQpgYGAKCiMjIyBQbG90cwoKIyMjIyBNb2xlY3VsYXIgV2VpZ2h0CgpgYGB7cn0KbXdfZGlzdHJpYnV0aW9uIDwtIGRhdGEuZnJhbWUoCiAgTW9sV2VpZ2h0ID0gYyhJUERCX21hdGNoaW5nJE1vbFdlaWdodCwgSVBEQl9tYXRjaGluZ19wcm90ZW9tZSRNb2xXZWlnaHQpLAogIHNldCA9IGMocmVwKCJQVGV4IiwgbGVuZ3RoKElQREJfbWF0Y2hpbmckTW9sV2VpZ2h0KSksIHJlcCgiUHJvdGVvbWUiLCBsZW5ndGgoSVBEQl9tYXRjaGluZ19wcm90ZW9tZSRNb2xXZWlnaHQpKSkKKQoKZ2dwbG90KG13X2Rpc3RyaWJ1dGlvbiwgYWVzKHggPSBzZXQsIHkgPSBNb2xXZWlnaHQpKSArCiAgZ2VvbV9ib3hwbG90KCkgKyAKICBzY2FsZV95X2xvZzEwKCkgKwogIHhsYWIoIiIpICsKICB5bGFiKCJNb2xlY3VsYXIgV2VpZ2h0IikKCnBkZigiTW9sV2VpZ2h0X2JveHBsb3QucGRmIiwgd2lkdGggPSAxMCwgaGVpZ2h0ID0gNykKbGFzdF9wbG90KCkKZGV2Lm9mZigpCmBgYAoKIyMjIyBJc29lbGVjdHJpYyBQb2ludAoKIyMjIyMgQm94cGxvdAoKYGBge3J9CnBpX2Rpc3RyaWJ1dGlvbiA8LSBkYXRhLmZyYW1lKAogIElQQ19wcm90ZWluID0gYyhJUERCX21hdGNoaW5nJElQQ19wcm90ZWluLCBJUERCX21hdGNoaW5nX3Byb3Rlb21lJElQQ19wcm90ZWluKSwKICBzZXQgPSBjKHJlcCgiUFRleCIsIGxlbmd0aChJUERCX21hdGNoaW5nJElQQ19wcm90ZWluKSksIHJlcCgiUHJvdGVvbWUiLCBsZW5ndGgoSVBEQl9tYXRjaGluZ19wcm90ZW9tZSRJUENfcHJvdGVpbikpKQopCgpnZ3Bsb3QocGlfZGlzdHJpYnV0aW9uLCBhZXMoeCA9IHNldCwgeSA9IElQQ19wcm90ZWluKSkgKwogIGdlb21fYm94cGxvdCgpICsgCiAgeGxhYigiIikgKwogIHlsYWIoIklQQ19wcm90ZWluIikKCnBkZigiSVBDX3Byb3RlaW5fYm94cGxvdC5wZGYiLCB3aWR0aCA9IDEwLCBoZWlnaHQgPSA3KQpsYXN0X3Bsb3QoKQpkZXYub2ZmKCkKYGBgCgojIyMjIyBEZW5zaXR5IFBsb3QKCkEgZGVuc2l0eSBwbG90IG9mIHRoZSBJUENfcHJvdGVpbiBkaXN0cmlidXRpb25zIGNvbXBhcmluZyBQVGV4LCBMYW5kdGhhbGVyLCBSaWNrIHVuaXF1ZSwgUmljayBoaWdoIGFuZCBwcm90ZW9tZSBpcyBwcmVwYXJlZC4KCmBgYHtyfQpyaWNrX2hpZ2hfaXAgPC0gZGF0YS5mcmFtZSgKICBJUENfcHJvdGVpbiA9IElQREJfbWF0Y2hpbmdfcHJvdGVvbWUkSVBDX3Byb3RlaW5bSVBEQl9tYXRjaGluZ19wcm90ZW9tZSRVbmlQcm90ICVpbiUgcmlja19oaWdoJFByb3RlaW5fSURdLAogIHNldCA9IHJlcCgiUmljay5oaWdoIiwgbnJvdyhyaWNrX2hpZ2gpKQopCgpyaWNrX3VuaXF1ZV9pcCA8LSBkYXRhLmZyYW1lKAogIElQQ19wcm90ZWluID0gSVBEQl9tYXRjaGluZ19wcm90ZW9tZSRJUENfcHJvdGVpbltJUERCX21hdGNoaW5nX3Byb3Rlb21lJFVuaVByb3QgJWluJSByaWNrX3VuaXF1ZSRQcm90ZWluX0lEXSwKICBzZXQgPSByZXAoIlJpY2sudW5pcXVlIiwgbnJvdyhyaWNrX3VuaXF1ZSkpCikKCiMgNiBhY2Nlc3Npb25zIG9mIHRoZSBMYW5kdGhhbGVyIGRhdGEgYXJlIG5vdCBpbmNsdWRlZCBpbiB0aGUgaXNvZWxlY3RyaWNwb2ludGRiIGFuZCB3aWxsIGJlIHJlbW92ZWQKCmxhbmR0aGFsZXJfaXAgPC0gZGF0YS5mcmFtZSgKICBJUENfcHJvdGVpbiA9IElQREJfbWF0Y2hpbmdfcHJvdGVvbWUkSVBDX3Byb3RlaW5bSVBEQl9tYXRjaGluZ19wcm90ZW9tZSRVbmlQcm90ICVpbiUgaGVrbVJCUCRVbmlwcm90LmVudHJ5Wy13aGljaChoZWttUkJQJFVuaXByb3QuZW50cnkgJWluJSBJUERCX21hdGNoaW5nX3Byb3Rlb21lJFVuaVByb3Q9PUZBTFNFKV1dLAogIHNldCA9IHJlcCgiTGFuZHRoYWxlci5tUkJQIiwgbGVuZ3RoKHdoaWNoKGhla21SQlAkVW5pcHJvdC5lbnRyeSAlaW4lIElQREJfbWF0Y2hpbmdfcHJvdGVvbWUkVW5pUHJvdD09VFJVRSkpKQopCmlwX2RlbnNpdHlfZGlzdHJpYnV0aW9uIDwtIGRhdGEuZnJhbWUoCiAgSVBDX3Byb3RlaW4gPSBjKElQREJfbWF0Y2hpbmckSVBDX3Byb3RlaW4sIGxhbmR0aGFsZXJfaXAkSVBDX3Byb3RlaW4sIHJpY2tfdW5pcXVlX2lwJElQQ19wcm90ZWluLCByaWNrX2hpZ2hfaXAkSVBDX3Byb3RlaW4sIElQREJfbWF0Y2hpbmdfcHJvdGVvbWUkSVBDX3Byb3RlaW4pLAogIHNldCA9IGMocmVwKCJQVGV4IiwgbnJvdyhJUERCX21hdGNoaW5nKSksIGFzLmNoYXJhY3RlcihsYW5kdGhhbGVyX2lwJHNldCksIGFzLmNoYXJhY3RlcihyaWNrX3VuaXF1ZV9pcCRzZXQpLCBhcy5jaGFyYWN0ZXIocmlja19oaWdoX2lwJHNldCksIHJlcCgiUHJvdGVvbWUiLCBucm93KElQREJfbWF0Y2hpbmdfcHJvdGVvbWUpKSkKKQoKZ2dwbG90Mjo6Z2dwbG90KGlwX2RlbnNpdHlfZGlzdHJpYnV0aW9uLCBhZXMoeCA9IElQQ19wcm90ZWluLCBjb2xvdXIgPSBzZXQpKSArCiAgZ2VvbV9kZW5zaXR5KCkKCnBkZigiSVBDX3Byb3RlaW5fZGVuc2l0eS5wZGYiLCB3aWR0aCA9IDEwLCBoZWlnaHQgPSA3KQpsYXN0X3Bsb3QoKQpkZXYub2ZmKCkKYGBgCgoKIyMjIyBIeWRyb3Bob2JpY2l0eQoKYGBge3J9Cmh5ZHJvcGhvYmljaXR5X2Rpc3RyaWJ1dGlvbiA8LSBkYXRhLmZyYW1lKAogIEh5ZHJvcGhvYmljaXR5ID0gYyhJUERCX21hdGNoaW5nJEh5ZHJvcGhvYmljaXR5LCBJUERCX21hdGNoaW5nX3Byb3Rlb21lJEh5ZHJvcGhvYmljaXR5KSwKICBzZXQgPSBjKHJlcCgiUFRleCIsIGxlbmd0aChJUERCX21hdGNoaW5nJEh5ZHJvcGhvYmljaXR5KSksIHJlcCgiUHJvdGVvbWUiLCBsZW5ndGgoSVBEQl9tYXRjaGluZ19wcm90ZW9tZSRIeWRyb3Bob2JpY2l0eSkpKQopCgpnZ3Bsb3QoaHlkcm9waG9iaWNpdHlfZGlzdHJpYnV0aW9uLCBhZXMoeCA9IHNldCwgeSA9IEh5ZHJvcGhvYmljaXR5KSkgKwogIGdlb21fYm94cGxvdCgpICsgCiAgeGxhYigiIikgKwogIHlsYWIoIkh5ZHJvcGhvYmljaXR5IikKCnBkZigiSHlkcm9waG9iaWNpdHlfYm94cGxvdC5wZGYiLCB3aWR0aCA9IDEwLCBoZWlnaHQgPSA3KQpsYXN0X3Bsb3QoKQpkZXYub2ZmKCkKYGBgCgojIyBSQkQKClRoZSBSQkQgZG9tYWluIGFubm90YXRpb24gaXMgcGVyZm9ybWVkIHVzaW5nIERBVklEIG9ubGluZSB0b29sLiBTaW5jZSBpdCBvbmx5IGFjY2VwdHMgbGlzdHMgd2l0aCBtYXhpbXVtIDMwMDAgZW50cmllcyAxMCByYW5kb21seSBzYW1wbGVkIGxpc3RzIHdlcmUgY3JlYXRlZC4KCmBgYHtyfQpzZXQuc2VlZCg0MikKYWNjZXNzaW9uc19TMSA8LSBzYW1wbGUoYWNjZXNzaW9ucywgMzAwMCkKYWNjZXNzaW9uc19TMiA8LSBzYW1wbGUoYWNjZXNzaW9ucywgMzAwMCkKYWNjZXNzaW9uc19TMyA8LSBzYW1wbGUoYWNjZXNzaW9ucywgMzAwMCkKYWNjZXNzaW9uc19TNCA8LSBzYW1wbGUoYWNjZXNzaW9ucywgMzAwMCkKYWNjZXNzaW9uc19TNSA8LSBzYW1wbGUoYWNjZXNzaW9ucywgMzAwMCkKYWNjZXNzaW9uc19TNiA8LSBzYW1wbGUoYWNjZXNzaW9ucywgMzAwMCkKYWNjZXNzaW9uc19TNyA8LSBzYW1wbGUoYWNjZXNzaW9ucywgMzAwMCkKYWNjZXNzaW9uc19TOCA8LSBzYW1wbGUoYWNjZXNzaW9ucywgMzAwMCkKYWNjZXNzaW9uc19TOSA8LSBzYW1wbGUoYWNjZXNzaW9ucywgMzAwMCkKYWNjZXNzaW9uc19TMTAgPC0gc2FtcGxlKGFjY2Vzc2lvbnMsIDMwMDApCgp3cml0ZS50YWJsZShhY2Nlc3Npb25zX1MxLCAiYWNjZXNzaW9uc19TMS50c3YiLCByb3cubmFtZXMgPSBGLCBjb2wubmFtZXMgPSBGKQp3cml0ZS50YWJsZShhY2Nlc3Npb25zX1MyLCAiYWNjZXNzaW9uc19TMi50c3YiLCByb3cubmFtZXMgPSBGLCBjb2wubmFtZXMgPSBGKQp3cml0ZS50YWJsZShhY2Nlc3Npb25zX1MzLCAiYWNjZXNzaW9uc19TMy50c3YiLCByb3cubmFtZXMgPSBGLCBjb2wubmFtZXMgPSBGKQp3cml0ZS50YWJsZShhY2Nlc3Npb25zX1M0LCAiYWNjZXNzaW9uc19TNC50c3YiLCByb3cubmFtZXMgPSBGLCBjb2wubmFtZXMgPSBGKQp3cml0ZS50YWJsZShhY2Nlc3Npb25zX1M1LCAiYWNjZXNzaW9uc19TNS50c3YiLCByb3cubmFtZXMgPSBGLCBjb2wubmFtZXMgPSBGKQp3cml0ZS50YWJsZShhY2Nlc3Npb25zX1M2LCAiYWNjZXNzaW9uc19TNi50c3YiLCByb3cubmFtZXMgPSBGLCBjb2wubmFtZXMgPSBGKQp3cml0ZS50YWJsZShhY2Nlc3Npb25zX1M3LCAiYWNjZXNzaW9uc19TNy50c3YiLCByb3cubmFtZXMgPSBGLCBjb2wubmFtZXMgPSBGKQp3cml0ZS50YWJsZShhY2Nlc3Npb25zX1M4LCAiYWNjZXNzaW9uc19TOC50c3YiLCByb3cubmFtZXMgPSBGLCBjb2wubmFtZXMgPSBGKQp3cml0ZS50YWJsZShhY2Nlc3Npb25zX1M5LCAiYWNjZXNzaW9uc19TOS50c3YiLCByb3cubmFtZXMgPSBGLCBjb2wubmFtZXMgPSBGKQp3cml0ZS50YWJsZShhY2Nlc3Npb25zX1MxMCwgImFjY2Vzc2lvbnNfUzEwLnRzdiIsIHJvdy5uYW1lcyA9IEYsIGNvbC5uYW1lcyA9IEYpCmBgYAoKCiMjIEdPCgpHTyB0ZXJtIGFuYWx5c2VzIHdhcyBwZXJmb3JtZWQgYnkgdGhlIFBBTlRIRVIgb25saW5lIHRvb2wgdXNpbmcgdGhlIGBhY2Nlc3Npb25zYCB0YWJsZS4KCiMgTWFzdGVyIFRhYmxlCgpBIG1hc3RlciB0YWJsZSBjb250YWluaW5nIGlkZW50aWZpZXJzLCBlbnJpY2htZW50cywgbWF0Y2hlcyBhbmQgYW5ub3RhdGlvbnMgaXMgY3JlYXRlZC4KCmBgYHtyfQojIGNyZWF0ZSB2ZWN0b3IgaG9sZGluZyByb3dzIG9mIHNpZ19ub3JtX01hc3NTcGVjRGF0YSB0aGF0IGFyZSBpbiBtYXRjaGluZ190YWJsZQpNU0RfdG9fTVQgPC0gd2hpY2goc2FwcGx5KFggPSBzaWdfbm9ybV9NYXNzU3BlY0RhdGEkTWFqb3JpdHkucHJvdGVpbi5JRHMsIEZVTiA9IGZ1bmN0aW9uKHgpdW5saXN0KHN0cnNwbGl0KGFzLmNoYXJhY3Rlcih4KSwgc3BsaXQ9IjsiKSkpICVpbiUgbWF0Y2hpbmdfdGFibGUkTWFqb3JpdHkucHJvdGVpbi5JRHMpCgptYXN0ZXJfdGFibGUgPC0gZGF0YS5mcmFtZSgKICBVbmlQcm90ID0gYWNjZXNzaW9ucywKICBNYWpvcml0eS5wcm90ZWluLklEcyA9IHNpZ19ub3JtX01hc3NTcGVjRGF0YSRNYWpvcml0eS5wcm90ZWluLklEc1tNU0RfdG9fTVRdLAogIE1QSUQucmV2aWV3ZWQgPSBhcy5jaGFyYWN0ZXIobWF0Y2hpbmdfdGFibGUkTVBJRHIpLAogIFByb3RlaW4ubmFtZSA9IHNpZ19ub3JtX01hc3NTcGVjRGF0YSRQcm90ZWluLm5hbWVzW01TRF90b19NVF0sCiAgR2VuZS5uYW1lID0gbWF0Y2hpbmdfdGFibGUkZ2VuZV9uYW1lLAogIEZDLlBUZXhfMDAxNS5tZWFuID0gc2lnX25vcm1fTWFzc1NwZWNEYXRhJEZDLlBUZXhfMDAxNS5tZWFuW01TRF90b19NVF0sCiAgcGFkai5QVGV4XzAwMTUgPSBzaWdfbm9ybV9NYXNzU3BlY0RhdGEkcGFkai5QVGV4XzAwMTVbTVNEX3RvX01UXSwKICBGQy5QVGV4XzAxNS5tZWFuID0gc2lnX25vcm1fTWFzc1NwZWNEYXRhJEZDLlBUZXhfMDE1Lm1lYW5bTVNEX3RvX01UXSwKICBwYWRqLlBUZXhfMDE1ID0gc2lnX25vcm1fTWFzc1NwZWNEYXRhJHBhZGouUFRleF8wMTVbTVNEX3RvX01UXSwKICBGQy5QVGV4XzE1Lm1lYW4gPSBzaWdfbm9ybV9NYXNzU3BlY0RhdGEkRkMuUFRleF8xNS5tZWFuW01TRF90b19NVF0sCiAgcGFkai5QVGV4XzE1ID0gc2lnX25vcm1fTWFzc1NwZWNEYXRhJHBhZGouUFRleF8xNVtNU0RfdG9fTVRdLAogIEdlcnN0YmVyZ2VyLlJCUCA9IG1hdGNoaW5nX3RhYmxlJEdlcnN0YmVyZ2VyLlJCUCwKICBHZXJzdGJlcmdlci5URiA9IG1hdGNoaW5nX3RhYmxlJEdlcnN0YmVyZ2VyLlRGLAogIExhbmR0aGFsZXIubVJCUCA9IG1hdGNoaW5nX3RhYmxlJExhbmR0aGFsZXIubVJCUCwKICBQcmVpc3MgPSBtYXRjaGluZ190YWJsZSRQcmVpc3MsCiAgQnJhbm5hbi5hbm5vdGF0ZWQgPSBtYXRjaGluZ190YWJsZSRCcmFubmFuLmFubm90YXRlZCwKICBCcmFubmFuLnByZWRpY3RlZCA9IG1hdGNoaW5nX3RhYmxlJEJyYW5uYW4ucHJlZGljdGVkLAogIEh1YnN0ZW5iZXJnZXIuUEJQID0gbWF0Y2hpbmdfdGFibGUkSHVic3RlbmJlcmdlci5QQlAsCiAgUmljay5oaWdoID0gbWF0Y2hpbmdfdGFibGUkUmljay5oaWdoLAogIFJpY2subG93ID0gbWF0Y2hpbmdfdGFibGUkUmljay5sb3csCiAgUmljay51bmlxdWUgPSBtYXRjaGluZ190YWJsZSRSaWNrLnVuaXF1ZSwKICBDYXJpYyA9IG1hdGNoaW5nX3RhYmxlJENhcmljLAogIElQQ19wcm90ZWluID0gSVBEQl9tYXRjaGluZyRJUENfcHJvdGVpbiwKICBNb2xXZWlnaHQgPSBJUERCX21hdGNoaW5nJE1vbFdlaWdodCwKICBIeWRyb3Bob2JpY2l0eSA9IElQREJfbWF0Y2hpbmckSHlkcm9waG9iaWNpdHkKKQoKd3JpdGUudGFibGUobWFzdGVyX3RhYmxlLCAibWFzdGVyX3RhYmxlLnRzdiIsIHNlcCA9ICJcdCIsIHJvdy5uYW1lcyA9IEYpCmBgYAoKCiMgU2Vzc2lvbiBJbmZvCgpgYGB7cn0Kc2Vzc2lvbkluZm8oKQpgYGA=
