# Supplementary material for: Purification of cross-linked RNA-protein complexes by phenol-toluol extraction
Source: Nat Commun. 2019 Mar 1;10:990. doi: 10.1038/s41467-019-08942-3 (PMC6397201; doi:10.1038/s41467-019-08942-3)
Supplement: Supplementary file 12 — Supplementary Data 9 [file 41467_2019_8942_MOESM12_ESM.zip › PTex_Supplementary_Salmonella_MS_analysis/PTex_Salmonella.nb.html]

HotPTex Salmonella


Code 

- Show All Code
- Hide All Code
- Download Rmd

# HotPTex Salmonella

- 1 Introduction
- 2 Data loading
  - 2.1 First filter
  - 2.2 Transformation
  - 2.3 Normalisation and trypsin check
  - 2.4 Contaminant removal
  - 2.5 Incomplete observation removal
- 3 Enrichment analysis
  - 3.1 Fold Changes
  - 3.2 Enrichment analysis
    - 3.2.1 Moderated t-test and Benjamini-Hochberg correction
  - 3.3 Mean intensities
  - 3.4 Quality control and results plots
  - 3.5 Replicate reproducibility
- 4 Plots

# 1 Introduction


```
#source("https://bioconductor.org/biocLite.R")
#biocLite()
library(ggplot2)
library(dplyr)
#biocLite("limma")
library(limma)
library(GGally)
library(tidyverse)
library(reshape2)
```

# 2 Data loading


```
### To load primary MS data
primaryMassSpecData <- read.table("inputdata/2017-12-21_PG_hotptex03.txt", header = TRUE, sep = "\t")
```


To ease later analyses, character vectors of columnnames are created.


```
intensities <- c("Intensity.HotPTex_DDM_NoXL_1",
               "Intensity.HotPTex_DDM_NoXL_2",
               "Intensity.HotPTex_DDM_XL_1",
               "Intensity.HotPTex_DDM_XL_2",
               "Intensity.HotPTex_PBS_NoXL_1",
               "Intensity.HotPTex_PBS_NoXL_2",
               "Intensity.HotPTex_PBS_XL_1",
               "Intensity.HotPTex_PBS_XL_2",
               "Intensity.Input_DDM_NoXL_1",
               "Intensity.Input_DDM_NoXL_2",
               "Intensity.Input_DDM_XL_1",
               "Intensity.Input_DDM_XL_2",
               "Intensity.Input_PBS_NoXL_1",
               "Intensity.Input_PBS_NoXL_2",
               "Intensity.Input_PBS_XL_1",
               "Intensity.Input_PBS_XL_2")
```


```
IBAQ <- c("iBAQ.HotPTex_DDM_NoXL_1",
               "iBAQ.HotPTex_DDM_NoXL_2",
               "iBAQ.HotPTex_DDM_XL_1",
               "iBAQ.HotPTex_DDM_XL_2",
               "iBAQ.HotPTex_PBS_NoXL_1",
               "iBAQ.HotPTex_PBS_NoXL_2",
               "iBAQ.HotPTex_PBS_XL_1",
               "iBAQ.HotPTex_PBS_XL_2",
               "iBAQ.Input_DDM_NoXL_1",
               "iBAQ.Input_DDM_NoXL_2",
               "iBAQ.Input_DDM_XL_1",
               "iBAQ.Input_DDM_XL_2",
               "iBAQ.Input_PBS_NoXL_1",
               "iBAQ.Input_PBS_NoXL_2",
               "iBAQ.Input_PBS_XL_1",
               "iBAQ.Input_PBS_XL_2")
```


```
LFQ <- c("LFQ.intensity.HotPTex_DDM_NoXL_1",
               "LFQ.intensity.HotPTex_DDM_NoXL_2",
               "LFQ.intensity.HotPTex_DDM_XL_1",
               "LFQ.intensity.HotPTex_DDM_XL_2",
               "LFQ.intensity.HotPTex_PBS_NoXL_1",
               "LFQ.intensity.HotPTex_PBS_NoXL_2",
               "LFQ.intensity.HotPTex_PBS_XL_1",
               "LFQ.intensity.HotPTex_PBS_XL_2",
               "LFQ.intensity.Input_DDM_NoXL_1",
               "LFQ.intensity.Input_DDM_NoXL_2",
               "LFQ.intensity.Input_DDM_XL_1",
               "LFQ.intensity.Input_DDM_XL_2",
               "LFQ.intensity.Input_PBS_NoXL_1",
               "LFQ.intensity.Input_PBS_NoXL_2",
               "LFQ.intensity.Input_PBS_XL_1",
               "LFQ.intensity.Input_PBS_XL_2")
```


## 2.1 First filter

In a first filtering step reverse and hits only identified by site are removed


```
MassSpecData <- primaryMassSpecData[primaryMassSpecData$Reverse != "+",]
MassSpecData <- MassSpecData[MassSpecData$Only.identified.by.site != "+",]
```

## 2.2 Transformation

All intensites are log2 transformed.


```
MassSpecData[c(intensities, IBAQ, LFQ)] <- log2(MassSpecData[c(intensities, IBAQ, LFQ)])
#colnames(MassSpecData)
```


Infinity values that resulted from log2 transformation are converted to `NA`:


```
is.na(MassSpecData[c(intensities,IBAQ,LFQ)]) <- sapply(MassSpecData[c(intensities,IBAQ,LFQ)], is.infinite)
```

## 2.3 Normalisation and trypsin check


```
trypsin = MassSpecData[MassSpecData$Majority.protein.IDs == "CON__P00761",]
#select genes you want to see patterns
ptn = c("CON__P00761")           #Trypsin
#select corresponding colors
cl = c(rgb(0.85,0,0))
for (k in c("intensities", "IBAQ", "LFQ")) {
  
  all <- melt(MassSpecData,id.vars=c("Majority.protein.IDs"), measure.vars=eval(parse(text = k)))
  
  
  tiff(paste("figures/Boxplot_",k,".tiff", sep = ""), width = 800, height = 1000, pointsize = 25)
  
  p1 <- ggplot(all, aes(variable,value), col(Majority.protein.IDs)) +     # Plot boxplot
    geom_boxplot(width = 1) +
    theme(axis.title.x = element_blank(),
          axis.text.x  = element_text(face = "bold", color = "black",
                                      angle=90, 
                                      vjust=0.5,
                                      hjust = 1,
                                      size=25),
          axis.title.y = element_text(face="bold",
                                      size=40,
                                      hjust = 0.5,
                                      vjust = 1.5),
          axis.text.y  = element_text(color = "black", size = 30)) +
    ylab(paste(k," (Log2)", sep = "")) +
    coord_cartesian(ylim = c(12, 40)) +
    scale_y_continuous(breaks=seq(12, 40, 4)) +
    scale_x_discrete(labels= eval(parse(text = k))) +
    scale_color_hue(l=65, c=65) +
    
    #Error: Incompatible lengths for set aesthetics: colour, x, y      <--   this error happens when one of your gene names is identified in more than one protein group (isoforms)
    
    geom_point(data = subset(all,Majority.protein.IDs %in% ptn), aes(x = variable, col = Majority.protein.IDs), size = 4) +
    geom_line(data = subset(all,Majority.protein.IDs %in% ptn), aes(x = variable, col = Majority.protein.IDs, group = Majority.protein.IDs) , size = 2, linetype = 1)
  
  plot(p1)
  
  graphics.off()
  rm(all)
   
}
rm(cl,k,p1)
```

## 2.4 Contaminant removal

After calculation of the normalisation factors the contaminants are removed from the data set.


```
MassSpecData <- MassSpecData[MassSpecData$Potential.contaminant != "+",]
```

## 2.5 Incomplete observation removal

We only want to consider those proteins, that were found in 2 out of 3 replicates of all experiments.


```
norm_MassSpecData <- MassSpecData
#PTex PBS only 
for(exp in c("HotPTex_PBS_noXL","HotPTex_PBS_XL")){  
  
  sub_MassSpecData <- select(norm_MassSpecData, matches(paste("iBAQ", exp, sep = ".")))
  x <- apply(sub_MassSpecData, 1, function(x)sum(!is.na(x))>1) #set to 1 if 2/3 or set to 2 if 3/3
  norm_MassSpecData <- norm_MassSpecData[x,]
}
```

# 3 Enrichment analysis

After preparation the fold changes between respective Input/PTex pairs can be calculated and a moderated t-test with following Benjamini-Hochberg correction is used to determine the false discovery rate (FDR).

## 3.1 Fold Changes

The fold changes are calculated by subtracting the log-transformed LFQ intensity values of the non-crosslinked (-CL) from the crosslinked (+CL) samples.


```
norm_MassSpecData$FC.Input_PBS.rep1 <- (norm_MassSpecData$iBAQ.Input_PBS_XL_1 - norm_MassSpecData$iBAQ.Input_PBS_NoXL_1)
norm_MassSpecData$FC.Input_PBS.rep2 <- (norm_MassSpecData$iBAQ.Input_PBS_XL_2 - norm_MassSpecData$iBAQ.Input_PBS_NoXL_2)
norm_MassSpecData$FC.Input_DDM.rep1 <- (norm_MassSpecData$iBAQ.Input_DDM_XL_1 - norm_MassSpecData$iBAQ.Input_DDM_NoXL_1)
norm_MassSpecData$FC.Input_DDM.rep2 <- (norm_MassSpecData$iBAQ.Input_DDM_XL_2 - norm_MassSpecData$iBAQ.Input_DDM_NoXL_2)
norm_MassSpecData$FC.PTex_PBS.rep1 <- (norm_MassSpecData$iBAQ.HotPTex_PBS_XL_1 - norm_MassSpecData$iBAQ.HotPTex_PBS_NoXL_1)
norm_MassSpecData$FC.PTex_PBS.rep2 <- (norm_MassSpecData$iBAQ.HotPTex_PBS_XL_2 - norm_MassSpecData$iBAQ.HotPTex_PBS_NoXL_2)
norm_MassSpecData$FC.PTex_DDM.rep1 <- (norm_MassSpecData$iBAQ.HotPTex_DDM_XL_1 - norm_MassSpecData$iBAQ.HotPTex_DDM_NoXL_1)
norm_MassSpecData$FC.PTex_DDM.rep2 <- (norm_MassSpecData$iBAQ.HotPTex_DDM_XL_2 - norm_MassSpecData$iBAQ.HotPTex_DDM_NoXL_2)
```


```
norm_MassSpecData$FC.Input_PBS.mean <- rowMeans(norm_MassSpecData[,180:181])
norm_MassSpecData$FC.Input_DDM.mean <- rowMeans(norm_MassSpecData[,182:183])
norm_MassSpecData$FC.PTex_PBS.mean <- rowMeans(norm_MassSpecData[,184:185])
norm_MassSpecData$FC.PTex_DDM.mean <- rowMeans(norm_MassSpecData[,186:187])
```

## 3.2 Enrichment analysis

### 3.2.1 Moderated t-test and Benjamini-Hochberg correction


```
pval =  eBayes(lmFit(norm_MassSpecData[,grep("FC.Input_PBS.rep",names(norm_MassSpecData))]))
norm_MassSpecData$pval.Input_PBS <- pval$p.value
norm_MassSpecData$padj.Input_PBS <-  p.adjust(norm_MassSpecData$pval.Input_PBS, method="BH")
pval =  eBayes(lmFit(norm_MassSpecData[,grep("FC.Input_DDM.rep",names(norm_MassSpecData))]))
norm_MassSpecData$pval.Input_DDM <- pval$p.value
norm_MassSpecData$padj.Input_DDM <-  p.adjust(norm_MassSpecData$pval.Input_DDM, method="BH")
pval = eBayes(lmFit(norm_MassSpecData[,grep("FC.PTex_PBS.rep",names(norm_MassSpecData))]))
norm_MassSpecData$pval.PTex_PBS <- pval$p.value
norm_MassSpecData$padj.PTex_PBS <- p.adjust(norm_MassSpecData$pval.PTex_PBS, method="BH")
pval = eBayes(lmFit(norm_MassSpecData[,grep("FC.PTex_DDM.rep",names(norm_MassSpecData))]))
norm_MassSpecData$pval.PTex_DDM <- pval$p.value
norm_MassSpecData$padj.PTex_DDM <- p.adjust(norm_MassSpecData$pval.PTex_DDM, method="BH")
```

## 3.3 Mean intensities


```
### iBAQ
norm_MassSpecData$iBAQ.Input_PBS_noCL_mean <- rowMeans(norm_MassSpecData[,c("iBAQ.Input_PBS_NoXL_1", "iBAQ.Input_PBS_NoXL_2")], na.rm = FALSE)
norm_MassSpecData$iBAQ.Input_DDM_noCL_mean <- rowMeans(norm_MassSpecData[,c("iBAQ.Input_DDM_NoXL_1", "iBAQ.Input_DDM_NoXL_2")], na.rm = FALSE)
norm_MassSpecData$iBAQ.Input_PBS_mean <- rowMeans(norm_MassSpecData[,c("iBAQ.Input_PBS_XL_1", "iBAQ.Input_PBS_XL_2")], na.rm = FALSE)
norm_MassSpecData$iBAQ.Input_DDM_mean <- rowMeans(norm_MassSpecData[,c("iBAQ.Input_DDM_XL_1", "iBAQ.Input_DDM_XL_2")], na.rm = FALSE)
norm_MassSpecData$iBAQ.PTex_PBS_noCL_mean <- rowMeans(norm_MassSpecData[,c("iBAQ.HotPTex_PBS_NoXL_1", "iBAQ.HotPTex_PBS_NoXL_2")], na.rm = FALSE)
norm_MassSpecData$iBAQ.PTex_DDM_noCL_mean <- rowMeans(norm_MassSpecData[,c("iBAQ.HotPTex_DDM_NoXL_1", "iBAQ.HotPTex_DDM_NoXL_2")], na.rm = FALSE)
norm_MassSpecData$iBAQ.PTex_PBS_mean <- rowMeans(norm_MassSpecData[,c("iBAQ.HotPTex_PBS_XL_1", "iBAQ.HotPTex_PBS_XL_2")], na.rm = FALSE)
norm_MassSpecData$iBAQ.PTex_DDM_mean <- rowMeans(norm_MassSpecData[,c("iBAQ.HotPTex_DDM_XL_1", "iBAQ.HotPTex_DDM_XL_2")], na.rm = FALSE)
write.table(norm_MassSpecData, file="MassSpecData_Exp3_iBAQ_notrypsin.txt", sep="\t", col.names=TRUE, row.names=FALSE)
```

## 3.4 Quality control and results plots

To produce pairwise scatter plots with correlation information we use the `ggpairs()` function from the `GGally` package:

## 3.5 Replicate reproducibility


```
scatter_theme <- theme(legend.position = "none", 
      panel.grid.major = element_blank(), 
      axis.ticks = element_blank(), 
      panel.border = element_rect(linetype = "solid", colour = "black", fill = NA))
ggplot <- function(...) ggplot2::ggplot(...) + scale_colour_manual(values = c("black","red")) + scale_fill_manual(values = c("black","red"))
unlockBinding("ggplot",parent.env(asNamespace("GGally")))
assign("ggplot",ggplot,parent.env(asNamespace("GGally")))
```


```
## Input PBS noCL
input_PBS_nocl_scatter <- ggpairs(norm_MassSpecData[,IBAQ[13:14]], 
        lower = list(continuous = wrap("cor", size = 7)), 
        upper = list(continuous = "smooth"),
        diag = list(continuous = "densityDiag")) + scatter_theme
## Input PBS CL
input_PBS_cl_scatter <- ggpairs(norm_MassSpecData[,IBAQ[15:16]], 
        lower = list(continuous = wrap("cor", size = 7)), 
        upper = list(continuous = "smooth"),
        diag = list(continuous = "densityDiag")) + scatter_theme
## HotPTex PBS noCL
hotptex_PBS_nocl_scatter <- ggpairs(norm_MassSpecData[,IBAQ[5:6]], 
        lower = list(continuous = wrap("cor", size = 7)), 
        upper = list(continuous = "smooth"),
        diag = list(continuous = "densityDiag"))  + scatter_theme
## HotPTex PBS Cl
hotptex_PBS_cl_scatter <- ggpairs(norm_MassSpecData[,IBAQ[7:8]], 
        lower = list(continuous = wrap("cor", size = 7)), 
        upper = list(continuous = "smooth"),
        diag = list(continuous = "densityDiag"))  + scatter_theme
```


```
input_PBS_nocl_scatter
```


```
input_PBS_cl_scatter
```


```
hotptex_PBS_nocl_scatter
```


```
hotptex_PBS_cl_scatter
```


Plots were saved with:


```
ggsave("figures/input_PBS_nocl_scatter.pdf", plot = input_PBS_nocl_scatter, units = "in", height = 7, width = 12)
ggsave("figures/input_PBS_cl_scatter.pdf", plot = input_PBS_cl_scatter, units = "in", height = 7, width = 12)

ggsave("figures/hotptex_PBS_nocl_scatter.pdf", plot = hotptex_PBS_nocl_scatter, units = "in", height = 7, width = 12)
ggsave("figures/hotptex_PBS_cl_scatter.pdf", plot = hotptex_PBS_cl_scatter, units = "in", height = 7, width = 12)
```


Tidy up:


```
rm(list = ls(pattern = "_scatter"))
```

# 4 Plots

Plot RBP classes


Safe plot


```
ggsave("figures/intensity_enrichment.pdf", plot = enr, units = "in", height = 7, width = 12)
```

LS0tCnRpdGxlOiAiSG90UFRleCBTYWxtb25lbGxhIgpvdXRwdXQ6CiAgaHRtbF9ub3RlYm9vazoKICAgIG51bWJlcl9zZWN0aW9uczogeWVzCiAgICBzbWFydDogbm8KICAgIHRvYzogeWVzCiAgcGRmX2RvY3VtZW50OgogICAgdG9jOiB5ZXMKLS0tCgojIEludHJvZHVjdGlvbgoKYGBge3J9CiNzb3VyY2UoImh0dHBzOi8vYmlvY29uZHVjdG9yLm9yZy9iaW9jTGl0ZS5SIikKI2Jpb2NMaXRlKCkKCmxpYnJhcnkoZ2dwbG90MikKbGlicmFyeShkcGx5cikKI2Jpb2NMaXRlKCJsaW1tYSIpCmxpYnJhcnkobGltbWEpCmxpYnJhcnkoR0dhbGx5KQpsaWJyYXJ5KHRpZHl2ZXJzZSkKbGlicmFyeShyZXNoYXBlMikKbGlicmFyeShSQ29sb3JCcmV3ZXIpCmBgYAoKCgojIERhdGEgbG9hZGluZwoKYGBge3IgbG9hZCBwcmltYXJ5IE1TIGRhdGF9CiMjIyBUbyBsb2FkIHByaW1hcnkgTVMgZGF0YQpwcmltYXJ5TWFzc1NwZWNEYXRhIDwtIHJlYWQudGFibGUoImlucHV0ZGF0YS8yMDE3LTEyLTIxX1BHX2hvdHB0ZXgwMy50eHQiLCBoZWFkZXIgPSBUUlVFLCBzZXAgPSAiXHQiKQpgYGAKClRvIGVhc2UgbGF0ZXIgYW5hbHlzZXMsIGNoYXJhY3RlciB2ZWN0b3JzIG9mIGNvbHVtbm5hbWVzIGFyZSBjcmVhdGVkLgpgYGB7ciBjb2xuYW1lcyBpbnRlbnNpdGllc30KaW50ZW5zaXRpZXMgPC0gYygiSW50ZW5zaXR5LkhvdFBUZXhfRERNX05vWExfMSIsCiAgICAgICAgICAgICAgICJJbnRlbnNpdHkuSG90UFRleF9ERE1fTm9YTF8yIiwKICAgICAgICAgICAgICAgIkludGVuc2l0eS5Ib3RQVGV4X0RETV9YTF8xIiwKICAgICAgICAgICAgICAgIkludGVuc2l0eS5Ib3RQVGV4X0RETV9YTF8yIiwKICAgICAgICAgICAgICAgIkludGVuc2l0eS5Ib3RQVGV4X1BCU19Ob1hMXzEiLAogICAgICAgICAgICAgICAiSW50ZW5zaXR5LkhvdFBUZXhfUEJTX05vWExfMiIsCiAgICAgICAgICAgICAgICJJbnRlbnNpdHkuSG90UFRleF9QQlNfWExfMSIsCiAgICAgICAgICAgICAgICJJbnRlbnNpdHkuSG90UFRleF9QQlNfWExfMiIsCiAgICAgICAgICAgICAgICJJbnRlbnNpdHkuSW5wdXRfRERNX05vWExfMSIsCiAgICAgICAgICAgICAgICJJbnRlbnNpdHkuSW5wdXRfRERNX05vWExfMiIsCiAgICAgICAgICAgICAgICJJbnRlbnNpdHkuSW5wdXRfRERNX1hMXzEiLAogICAgICAgICAgICAgICAiSW50ZW5zaXR5LklucHV0X0RETV9YTF8yIiwKICAgICAgICAgICAgICAgIkludGVuc2l0eS5JbnB1dF9QQlNfTm9YTF8xIiwKICAgICAgICAgICAgICAgIkludGVuc2l0eS5JbnB1dF9QQlNfTm9YTF8yIiwKICAgICAgICAgICAgICAgIkludGVuc2l0eS5JbnB1dF9QQlNfWExfMSIsCiAgICAgICAgICAgICAgICJJbnRlbnNpdHkuSW5wdXRfUEJTX1hMXzIiKQpgYGAKCmBgYHtyIGNvbG5hbWVzIGlCQVF9CklCQVEgPC0gYygiaUJBUS5Ib3RQVGV4X0RETV9Ob1hMXzEiLAogICAgICAgICAgICAgICAiaUJBUS5Ib3RQVGV4X0RETV9Ob1hMXzIiLAogICAgICAgICAgICAgICAiaUJBUS5Ib3RQVGV4X0RETV9YTF8xIiwKICAgICAgICAgICAgICAgImlCQVEuSG90UFRleF9ERE1fWExfMiIsCiAgICAgICAgICAgICAgICJpQkFRLkhvdFBUZXhfUEJTX05vWExfMSIsCiAgICAgICAgICAgICAgICJpQkFRLkhvdFBUZXhfUEJTX05vWExfMiIsCiAgICAgICAgICAgICAgICJpQkFRLkhvdFBUZXhfUEJTX1hMXzEiLAogICAgICAgICAgICAgICAiaUJBUS5Ib3RQVGV4X1BCU19YTF8yIiwKICAgICAgICAgICAgICAgImlCQVEuSW5wdXRfRERNX05vWExfMSIsCiAgICAgICAgICAgICAgICJpQkFRLklucHV0X0RETV9Ob1hMXzIiLAogICAgICAgICAgICAgICAiaUJBUS5JbnB1dF9ERE1fWExfMSIsCiAgICAgICAgICAgICAgICJpQkFRLklucHV0X0RETV9YTF8yIiwKICAgICAgICAgICAgICAgImlCQVEuSW5wdXRfUEJTX05vWExfMSIsCiAgICAgICAgICAgICAgICJpQkFRLklucHV0X1BCU19Ob1hMXzIiLAogICAgICAgICAgICAgICAiaUJBUS5JbnB1dF9QQlNfWExfMSIsCiAgICAgICAgICAgICAgICJpQkFRLklucHV0X1BCU19YTF8yIikKYGBgCgpgYGB7ciBjb2xuYW1lcyBMRlF9CkxGUSA8LSBjKCJMRlEuaW50ZW5zaXR5LkhvdFBUZXhfRERNX05vWExfMSIsCiAgICAgICAgICAgICAgICJMRlEuaW50ZW5zaXR5LkhvdFBUZXhfRERNX05vWExfMiIsCiAgICAgICAgICAgICAgICJMRlEuaW50ZW5zaXR5LkhvdFBUZXhfRERNX1hMXzEiLAogICAgICAgICAgICAgICAiTEZRLmludGVuc2l0eS5Ib3RQVGV4X0RETV9YTF8yIiwKICAgICAgICAgICAgICAgIkxGUS5pbnRlbnNpdHkuSG90UFRleF9QQlNfTm9YTF8xIiwKICAgICAgICAgICAgICAgIkxGUS5pbnRlbnNpdHkuSG90UFRleF9QQlNfTm9YTF8yIiwKICAgICAgICAgICAgICAgIkxGUS5pbnRlbnNpdHkuSG90UFRleF9QQlNfWExfMSIsCiAgICAgICAgICAgICAgICJMRlEuaW50ZW5zaXR5LkhvdFBUZXhfUEJTX1hMXzIiLAogICAgICAgICAgICAgICAiTEZRLmludGVuc2l0eS5JbnB1dF9ERE1fTm9YTF8xIiwKICAgICAgICAgICAgICAgIkxGUS5pbnRlbnNpdHkuSW5wdXRfRERNX05vWExfMiIsCiAgICAgICAgICAgICAgICJMRlEuaW50ZW5zaXR5LklucHV0X0RETV9YTF8xIiwKICAgICAgICAgICAgICAgIkxGUS5pbnRlbnNpdHkuSW5wdXRfRERNX1hMXzIiLAogICAgICAgICAgICAgICAiTEZRLmludGVuc2l0eS5JbnB1dF9QQlNfTm9YTF8xIiwKICAgICAgICAgICAgICAgIkxGUS5pbnRlbnNpdHkuSW5wdXRfUEJTX05vWExfMiIsCiAgICAgICAgICAgICAgICJMRlEuaW50ZW5zaXR5LklucHV0X1BCU19YTF8xIiwKICAgICAgICAgICAgICAgIkxGUS5pbnRlbnNpdHkuSW5wdXRfUEJTX1hMXzIiKQpgYGAKCgojIyBGaXJzdCBmaWx0ZXIKCkluIGEgZmlyc3QgZmlsdGVyaW5nIHN0ZXAgcmV2ZXJzZSBhbmQgaGl0cyBvbmx5IGlkZW50aWZpZWQgYnkgc2l0ZSBhcmUgcmVtb3ZlZAoKYGBge3IgZmlyc3QgZmlsdGVyIHN0ZXB9Ck1hc3NTcGVjRGF0YSA8LSBwcmltYXJ5TWFzc1NwZWNEYXRhW3ByaW1hcnlNYXNzU3BlY0RhdGEkUmV2ZXJzZSAhPSAiKyIsXQpNYXNzU3BlY0RhdGEgPC0gTWFzc1NwZWNEYXRhW01hc3NTcGVjRGF0YSRPbmx5LmlkZW50aWZpZWQuYnkuc2l0ZSAhPSAiKyIsXQpgYGAKCgoKIyMgVHJhbnNmb3JtYXRpb24KCkFsbCBpbnRlbnNpdGVzIGFyZSBsb2cyIHRyYW5zZm9ybWVkLgoKYGBge3IgbG9nMiB0cmFuc2Zvcm1hdGlvbn0KTWFzc1NwZWNEYXRhW2MoaW50ZW5zaXRpZXMsIElCQVEsIExGUSldIDwtIGxvZzIoTWFzc1NwZWNEYXRhW2MoaW50ZW5zaXRpZXMsIElCQVEsIExGUSldKQoKI2NvbG5hbWVzKE1hc3NTcGVjRGF0YSkKCmBgYAoKSW5maW5pdHkgdmFsdWVzIHRoYXQgcmVzdWx0ZWQgZnJvbSBsb2cyIHRyYW5zZm9ybWF0aW9uIGFyZSBjb252ZXJ0ZWQgdG8gYE5BYDoKCmBgYHtyIGNvbnZlcnNpb24gb2YgaW5maW5pdHkgdmFsdWVzfQppcy5uYShNYXNzU3BlY0RhdGFbYyhpbnRlbnNpdGllcyxJQkFRLExGUSldKSA8LSBzYXBwbHkoTWFzc1NwZWNEYXRhW2MoaW50ZW5zaXRpZXMsSUJBUSxMRlEpXSwgaXMuaW5maW5pdGUpCmBgYAoKCgoKIyNOb3JtYWxpc2F0aW9uIGFuZCB0cnlwc2luIGNoZWNrCgoKYGBge3J9Cgp0cnlwc2luID0gTWFzc1NwZWNEYXRhW01hc3NTcGVjRGF0YSRNYWpvcml0eS5wcm90ZWluLklEcyA9PSAiQ09OX19QMDA3NjEiLF0KCiNzZWxlY3QgZ2VuZXMgeW91IHdhbnQgdG8gc2VlIHBhdHRlcm5zCnB0biA9IGMoIkNPTl9fUDAwNzYxIikgICAgICAgICAgICNUcnlwc2luCiNzZWxlY3QgY29ycmVzcG9uZGluZyBjb2xvcnMKY2wgPSBjKHJnYigwLjg1LDAsMCkpCgpmb3IgKGsgaW4gYygiaW50ZW5zaXRpZXMiLCAiSUJBUSIsICJMRlEiKSkgewogIAogIGFsbCA8LSBtZWx0KE1hc3NTcGVjRGF0YSxpZC52YXJzPWMoIk1ham9yaXR5LnByb3RlaW4uSURzIiksIG1lYXN1cmUudmFycz1ldmFsKHBhcnNlKHRleHQgPSBrKSkpCiAgCiAgCiAgdGlmZihwYXN0ZSgiZmlndXJlcy9Cb3hwbG90XyIsaywiLnRpZmYiLCBzZXAgPSAiIiksIHdpZHRoID0gODAwLCBoZWlnaHQgPSAxMDAwLCBwb2ludHNpemUgPSAyNSkKICAKICBwMSA8LSBnZ3Bsb3QoYWxsLCBhZXModmFyaWFibGUsdmFsdWUpLCBjb2woTWFqb3JpdHkucHJvdGVpbi5JRHMpKSArICAgICAjIFBsb3QgYm94cGxvdAogICAgZ2VvbV9ib3hwbG90KHdpZHRoID0gMSkgKwogICAgdGhlbWUoYXhpcy50aXRsZS54ID0gZWxlbWVudF9ibGFuaygpLAogICAgICAgICAgYXhpcy50ZXh0LnggID0gZWxlbWVudF90ZXh0KGZhY2UgPSAiYm9sZCIsIGNvbG9yID0gImJsYWNrIiwKICAgICAgICAgICAgICAgICAgICAgICAgICAgICAgICAgICAgICBhbmdsZT05MCwgCiAgICAgICAgICAgICAgICAgICAgICAgICAgICAgICAgICAgICAgdmp1c3Q9MC41LAogICAgICAgICAgICAgICAgICAgICAgICAgICAgICAgICAgICAgIGhqdXN0ID0gMSwKICAgICAgICAgICAgICAgICAgICAgICAgICAgICAgICAgICAgICBzaXplPTI1KSwKICAgICAgICAgIGF4aXMudGl0bGUueSA9IGVsZW1lbnRfdGV4dChmYWNlPSJib2xkIiwKICAgICAgICAgICAgICAgICAgICAgICAgICAgICAgICAgICAgICBzaXplPTQwLAogICAgICAgICAgICAgICAgICAgICAgICAgICAgICAgICAgICAgIGhqdXN0ID0gMC41LAogICAgICAgICAgICAgICAgICAgICAgICAgICAgICAgICAgICAgIHZqdXN0ID0gMS41KSwKICAgICAgICAgIGF4aXMudGV4dC55ICA9IGVsZW1lbnRfdGV4dChjb2xvciA9ICJibGFjayIsIHNpemUgPSAzMCkpICsKICAgIHlsYWIocGFzdGUoaywiIChMb2cyKSIsIHNlcCA9ICIiKSkgKwogICAgY29vcmRfY2FydGVzaWFuKHlsaW0gPSBjKDEyLCA0MCkpICsKICAgIHNjYWxlX3lfY29udGludW91cyhicmVha3M9c2VxKDEyLCA0MCwgNCkpICsKICAgIHNjYWxlX3hfZGlzY3JldGUobGFiZWxzPSBldmFsKHBhcnNlKHRleHQgPSBrKSkpICsKICAgIHNjYWxlX2NvbG9yX2h1ZShsPTY1LCBjPTY1KSArCiAgICAKICAgICNFcnJvcjogSW5jb21wYXRpYmxlIGxlbmd0aHMgZm9yIHNldCBhZXN0aGV0aWNzOiBjb2xvdXIsIHgsIHkgICAgICA8LS0gICB0aGlzIGVycm9yIGhhcHBlbnMgd2hlbiBvbmUgb2YgeW91ciBnZW5lIG5hbWVzIGlzIGlkZW50aWZpZWQgaW4gbW9yZSB0aGFuIG9uZSBwcm90ZWluIGdyb3VwIChpc29mb3JtcykKICAgIAogICAgZ2VvbV9wb2ludChkYXRhID0gc3Vic2V0KGFsbCxNYWpvcml0eS5wcm90ZWluLklEcyAlaW4lIHB0biksIGFlcyh4ID0gdmFyaWFibGUsIGNvbCA9IE1ham9yaXR5LnByb3RlaW4uSURzKSwgc2l6ZSA9IDQpICsKICAgIGdlb21fbGluZShkYXRhID0gc3Vic2V0KGFsbCxNYWpvcml0eS5wcm90ZWluLklEcyAlaW4lIHB0biksIGFlcyh4ID0gdmFyaWFibGUsIGNvbCA9IE1ham9yaXR5LnByb3RlaW4uSURzLCBncm91cCA9IE1ham9yaXR5LnByb3RlaW4uSURzKSAsIHNpemUgPSAyLCBsaW5ldHlwZSA9IDEpCiAgCiAgcGxvdChwMSkKICAKICBncmFwaGljcy5vZmYoKQogIHJtKGFsbCkKICAgCn0KCnJtKGNsLGsscDEpCmBgYAoKCiMjIENvbnRhbWluYW50IHJlbW92YWwKCkFmdGVyIGNhbGN1bGF0aW9uIG9mIHRoZSBub3JtYWxpc2F0aW9uIGZhY3RvcnMgdGhlIGNvbnRhbWluYW50cyBhcmUgcmVtb3ZlZCBmcm9tIHRoZSBkYXRhIHNldC4KCmBgYHtyIGNvbnRhbWluYW50IHJlbW92YWx9Ck1hc3NTcGVjRGF0YSA8LSBNYXNzU3BlY0RhdGFbTWFzc1NwZWNEYXRhJFBvdGVudGlhbC5jb250YW1pbmFudCAhPSAiKyIsXQpgYGAKCgojIyBJbmNvbXBsZXRlIG9ic2VydmF0aW9uIHJlbW92YWwKCldlIG9ubHkgd2FudCB0byBjb25zaWRlciB0aG9zZSBwcm90ZWlucywgdGhhdCB3ZXJlIGZvdW5kIGluIDIgb3V0IG9mIDMgcmVwbGljYXRlcyBvZiBhbGwgZXhwZXJpbWVudHMuCgpgYGB7ciByZW1vdmFsIG9mIGluY29tcGxldGUgb2JzZXJ2YXRpb25zfQoKbm9ybV9NYXNzU3BlY0RhdGEgPC0gTWFzc1NwZWNEYXRhCgojUFRleCBQQlMgb25seSAKZm9yKGV4cCBpbiBjKCJIb3RQVGV4X1BCU19ub1hMIiwiSG90UFRleF9QQlNfWEwiKSl7ICAKICAKICBzdWJfTWFzc1NwZWNEYXRhIDwtIHNlbGVjdChub3JtX01hc3NTcGVjRGF0YSwgbWF0Y2hlcyhwYXN0ZSgiaUJBUSIsIGV4cCwgc2VwID0gIi4iKSkpCiAgeCA8LSBhcHBseShzdWJfTWFzc1NwZWNEYXRhLCAxLCBmdW5jdGlvbih4KXN1bSghaXMubmEoeCkpPjEpICNzZXQgdG8gMSBpZiAyLzMgb3Igc2V0IHRvIDIgaWYgMy8zCiAgbm9ybV9NYXNzU3BlY0RhdGEgPC0gbm9ybV9NYXNzU3BlY0RhdGFbeCxdCn0KYGBgCgoKIyBFbnJpY2htZW50IGFuYWx5c2lzCgpBZnRlciBwcmVwYXJhdGlvbiB0aGUgZm9sZCBjaGFuZ2VzIGJldHdlZW4gcmVzcGVjdGl2ZSBJbnB1dC9QVGV4IHBhaXJzIGNhbiBiZSBjYWxjdWxhdGVkIGFuZCBhIG1vZGVyYXRlZCB0LXRlc3Qgd2l0aCBmb2xsb3dpbmcgQmVuamFtaW5pLUhvY2hiZXJnIGNvcnJlY3Rpb24gaXMgdXNlZCB0byBkZXRlcm1pbmUgdGhlIGZhbHNlIGRpc2NvdmVyeSByYXRlIChGRFIpLgoKIyMgRm9sZCBDaGFuZ2VzCgpUaGUgZm9sZCBjaGFuZ2VzIGFyZSBjYWxjdWxhdGVkIGJ5IHN1YnRyYWN0aW5nIHRoZSBsb2ctdHJhbnNmb3JtZWQgTEZRIGludGVuc2l0eSB2YWx1ZXMgb2YgdGhlIG5vbi1jcm9zc2xpbmtlZCAoLUNMKSBmcm9tIHRoZSBjcm9zc2xpbmtlZCAoK0NMKSBzYW1wbGVzLgoKYGBge3IgY2FsY3VsYXRpb24gb2YgaW5kaXZpZHVhbCBmb2xkIGNoYW5nZXN9Cm5vcm1fTWFzc1NwZWNEYXRhJEZDLklucHV0X1BCUy5yZXAxIDwtIChub3JtX01hc3NTcGVjRGF0YSRpQkFRLklucHV0X1BCU19YTF8xIC0gbm9ybV9NYXNzU3BlY0RhdGEkaUJBUS5JbnB1dF9QQlNfTm9YTF8xKQpub3JtX01hc3NTcGVjRGF0YSRGQy5JbnB1dF9QQlMucmVwMiA8LSAobm9ybV9NYXNzU3BlY0RhdGEkaUJBUS5JbnB1dF9QQlNfWExfMiAtIG5vcm1fTWFzc1NwZWNEYXRhJGlCQVEuSW5wdXRfUEJTX05vWExfMikKCgpub3JtX01hc3NTcGVjRGF0YSRGQy5JbnB1dF9ERE0ucmVwMSA8LSAobm9ybV9NYXNzU3BlY0RhdGEkaUJBUS5JbnB1dF9ERE1fWExfMSAtIG5vcm1fTWFzc1NwZWNEYXRhJGlCQVEuSW5wdXRfRERNX05vWExfMSkKbm9ybV9NYXNzU3BlY0RhdGEkRkMuSW5wdXRfRERNLnJlcDIgPC0gKG5vcm1fTWFzc1NwZWNEYXRhJGlCQVEuSW5wdXRfRERNX1hMXzIgLSBub3JtX01hc3NTcGVjRGF0YSRpQkFRLklucHV0X0RETV9Ob1hMXzIpCgoKbm9ybV9NYXNzU3BlY0RhdGEkRkMuUFRleF9QQlMucmVwMSA8LSAobm9ybV9NYXNzU3BlY0RhdGEkaUJBUS5Ib3RQVGV4X1BCU19YTF8xIC0gbm9ybV9NYXNzU3BlY0RhdGEkaUJBUS5Ib3RQVGV4X1BCU19Ob1hMXzEpCm5vcm1fTWFzc1NwZWNEYXRhJEZDLlBUZXhfUEJTLnJlcDIgPC0gKG5vcm1fTWFzc1NwZWNEYXRhJGlCQVEuSG90UFRleF9QQlNfWExfMiAtIG5vcm1fTWFzc1NwZWNEYXRhJGlCQVEuSG90UFRleF9QQlNfTm9YTF8yKQoKbm9ybV9NYXNzU3BlY0RhdGEkRkMuUFRleF9ERE0ucmVwMSA8LSAobm9ybV9NYXNzU3BlY0RhdGEkaUJBUS5Ib3RQVGV4X0RETV9YTF8xIC0gbm9ybV9NYXNzU3BlY0RhdGEkaUJBUS5Ib3RQVGV4X0RETV9Ob1hMXzEpCm5vcm1fTWFzc1NwZWNEYXRhJEZDLlBUZXhfRERNLnJlcDIgPC0gKG5vcm1fTWFzc1NwZWNEYXRhJGlCQVEuSG90UFRleF9ERE1fWExfMiAtIG5vcm1fTWFzc1NwZWNEYXRhJGlCQVEuSG90UFRleF9ERE1fTm9YTF8yKQoKYGBgCgpgYGB7ciBjYWxjdWxhdGlvbiBvZiBtZWFuIGZvbGQgY2hhbmdlc30Kbm9ybV9NYXNzU3BlY0RhdGEkRkMuSW5wdXRfUEJTLm1lYW4gPC0gcm93TWVhbnMobm9ybV9NYXNzU3BlY0RhdGFbLDE4MDoxODFdKQpub3JtX01hc3NTcGVjRGF0YSRGQy5JbnB1dF9ERE0ubWVhbiA8LSByb3dNZWFucyhub3JtX01hc3NTcGVjRGF0YVssMTgyOjE4M10pCgpub3JtX01hc3NTcGVjRGF0YSRGQy5QVGV4X1BCUy5tZWFuIDwtIHJvd01lYW5zKG5vcm1fTWFzc1NwZWNEYXRhWywxODQ6MTg1XSkKbm9ybV9NYXNzU3BlY0RhdGEkRkMuUFRleF9ERE0ubWVhbiA8LSByb3dNZWFucyhub3JtX01hc3NTcGVjRGF0YVssMTg2OjE4N10pCgpgYGAKCiMjRW5yaWNobWVudCBhbmFseXNpcwoKIyMjIE1vZGVyYXRlZCB0LXRlc3QgYW5kIEJlbmphbWluaS1Ib2NoYmVyZyBjb3JyZWN0aW9uCmBgYHtyIG1vZCB0LXRlc3QgYW5kIEJIIGNvcnJlY3Rpb259CnB2YWwgPSAgZUJheWVzKGxtRml0KG5vcm1fTWFzc1NwZWNEYXRhWyxncmVwKCJGQy5JbnB1dF9QQlMucmVwIixuYW1lcyhub3JtX01hc3NTcGVjRGF0YSkpXSkpCm5vcm1fTWFzc1NwZWNEYXRhJHB2YWwuSW5wdXRfUEJTIDwtIHB2YWwkcC52YWx1ZQpub3JtX01hc3NTcGVjRGF0YSRwYWRqLklucHV0X1BCUyA8LSAgcC5hZGp1c3Qobm9ybV9NYXNzU3BlY0RhdGEkcHZhbC5JbnB1dF9QQlMsIG1ldGhvZD0iQkgiKQoKcHZhbCA9ICBlQmF5ZXMobG1GaXQobm9ybV9NYXNzU3BlY0RhdGFbLGdyZXAoIkZDLklucHV0X0RETS5yZXAiLG5hbWVzKG5vcm1fTWFzc1NwZWNEYXRhKSldKSkKbm9ybV9NYXNzU3BlY0RhdGEkcHZhbC5JbnB1dF9ERE0gPC0gcHZhbCRwLnZhbHVlCm5vcm1fTWFzc1NwZWNEYXRhJHBhZGouSW5wdXRfRERNIDwtICBwLmFkanVzdChub3JtX01hc3NTcGVjRGF0YSRwdmFsLklucHV0X0RETSwgbWV0aG9kPSJCSCIpCgoKcHZhbCA9IGVCYXllcyhsbUZpdChub3JtX01hc3NTcGVjRGF0YVssZ3JlcCgiRkMuUFRleF9QQlMucmVwIixuYW1lcyhub3JtX01hc3NTcGVjRGF0YSkpXSkpCm5vcm1fTWFzc1NwZWNEYXRhJHB2YWwuUFRleF9QQlMgPC0gcHZhbCRwLnZhbHVlCm5vcm1fTWFzc1NwZWNEYXRhJHBhZGouUFRleF9QQlMgPC0gcC5hZGp1c3Qobm9ybV9NYXNzU3BlY0RhdGEkcHZhbC5QVGV4X1BCUywgbWV0aG9kPSJCSCIpCgpwdmFsID0gZUJheWVzKGxtRml0KG5vcm1fTWFzc1NwZWNEYXRhWyxncmVwKCJGQy5QVGV4X0RETS5yZXAiLG5hbWVzKG5vcm1fTWFzc1NwZWNEYXRhKSldKSkKbm9ybV9NYXNzU3BlY0RhdGEkcHZhbC5QVGV4X0RETSA8LSBwdmFsJHAudmFsdWUKbm9ybV9NYXNzU3BlY0RhdGEkcGFkai5QVGV4X0RETSA8LSBwLmFkanVzdChub3JtX01hc3NTcGVjRGF0YSRwdmFsLlBUZXhfRERNLCBtZXRob2Q9IkJIIikKCgpgYGAKCgojIyBNZWFuIGludGVuc2l0aWVzCgoKYGBge3J9CgojIyMgaUJBUQpub3JtX01hc3NTcGVjRGF0YSRpQkFRLklucHV0X1BCU19ub0NMX21lYW4gPC0gcm93TWVhbnMobm9ybV9NYXNzU3BlY0RhdGFbLGMoImlCQVEuSW5wdXRfUEJTX05vWExfMSIsICJpQkFRLklucHV0X1BCU19Ob1hMXzIiKV0sIG5hLnJtID0gRkFMU0UpCm5vcm1fTWFzc1NwZWNEYXRhJGlCQVEuSW5wdXRfRERNX25vQ0xfbWVhbiA8LSByb3dNZWFucyhub3JtX01hc3NTcGVjRGF0YVssYygiaUJBUS5JbnB1dF9ERE1fTm9YTF8xIiwgImlCQVEuSW5wdXRfRERNX05vWExfMiIpXSwgbmEucm0gPSBGQUxTRSkKbm9ybV9NYXNzU3BlY0RhdGEkaUJBUS5JbnB1dF9QQlNfbWVhbiA8LSByb3dNZWFucyhub3JtX01hc3NTcGVjRGF0YVssYygiaUJBUS5JbnB1dF9QQlNfWExfMSIsICJpQkFRLklucHV0X1BCU19YTF8yIildLCBuYS5ybSA9IEZBTFNFKQpub3JtX01hc3NTcGVjRGF0YSRpQkFRLklucHV0X0RETV9tZWFuIDwtIHJvd01lYW5zKG5vcm1fTWFzc1NwZWNEYXRhWyxjKCJpQkFRLklucHV0X0RETV9YTF8xIiwgImlCQVEuSW5wdXRfRERNX1hMXzIiKV0sIG5hLnJtID0gRkFMU0UpCgpub3JtX01hc3NTcGVjRGF0YSRpQkFRLlBUZXhfUEJTX25vQ0xfbWVhbiA8LSByb3dNZWFucyhub3JtX01hc3NTcGVjRGF0YVssYygiaUJBUS5Ib3RQVGV4X1BCU19Ob1hMXzEiLCAiaUJBUS5Ib3RQVGV4X1BCU19Ob1hMXzIiKV0sIG5hLnJtID0gRkFMU0UpCm5vcm1fTWFzc1NwZWNEYXRhJGlCQVEuUFRleF9ERE1fbm9DTF9tZWFuIDwtIHJvd01lYW5zKG5vcm1fTWFzc1NwZWNEYXRhWyxjKCJpQkFRLkhvdFBUZXhfRERNX05vWExfMSIsICJpQkFRLkhvdFBUZXhfRERNX05vWExfMiIpXSwgbmEucm0gPSBGQUxTRSkKbm9ybV9NYXNzU3BlY0RhdGEkaUJBUS5QVGV4X1BCU19tZWFuIDwtIHJvd01lYW5zKG5vcm1fTWFzc1NwZWNEYXRhWyxjKCJpQkFRLkhvdFBUZXhfUEJTX1hMXzEiLCAiaUJBUS5Ib3RQVGV4X1BCU19YTF8yIildLCBuYS5ybSA9IEZBTFNFKQpub3JtX01hc3NTcGVjRGF0YSRpQkFRLlBUZXhfRERNX21lYW4gPC0gcm93TWVhbnMobm9ybV9NYXNzU3BlY0RhdGFbLGMoImlCQVEuSG90UFRleF9ERE1fWExfMSIsICJpQkFRLkhvdFBUZXhfRERNX1hMXzIiKV0sIG5hLnJtID0gRkFMU0UpCgoKd3JpdGUudGFibGUobm9ybV9NYXNzU3BlY0RhdGEsIGZpbGU9Ik1hc3NTcGVjRGF0YV9FeHAzX2lCQVFfbm90cnlwc2luLnR4dCIsIHNlcD0iXHQiLCBjb2wubmFtZXM9VFJVRSwgcm93Lm5hbWVzPUZBTFNFKQoKYGBgCgojIyBRdWFsaXR5IGNvbnRyb2wgYW5kIHJlc3VsdHMgcGxvdHMKCgpUbyBwcm9kdWNlIHBhaXJ3aXNlIHNjYXR0ZXIgcGxvdHMgd2l0aCBjb3JyZWxhdGlvbiBpbmZvcm1hdGlvbiB3ZSB1c2UgdGhlIGBnZ3BhaXJzKClgIGZ1bmN0aW9uIGZyb20gdGhlIGBHR2FsbHlgIHBhY2thZ2U6CgojIyBSZXBsaWNhdGUgcmVwcm9kdWNpYmlsaXR5CmBgYHtyfQpzY2F0dGVyX3RoZW1lIDwtIHRoZW1lKGxlZ2VuZC5wb3NpdGlvbiA9ICJub25lIiwgCiAgICAgIHBhbmVsLmdyaWQubWFqb3IgPSBlbGVtZW50X2JsYW5rKCksIAogICAgICBheGlzLnRpY2tzID0gZWxlbWVudF9ibGFuaygpLCAKICAgICAgcGFuZWwuYm9yZGVyID0gZWxlbWVudF9yZWN0KGxpbmV0eXBlID0gInNvbGlkIiwgY29sb3VyID0gImJsYWNrIiwgZmlsbCA9IE5BKSkKCmdncGxvdCA8LSBmdW5jdGlvbiguLi4pIGdncGxvdDI6OmdncGxvdCguLi4pICsgc2NhbGVfY29sb3VyX21hbnVhbCh2YWx1ZXMgPSBjKCJibGFjayIsInJlZCIpKSArIHNjYWxlX2ZpbGxfbWFudWFsKHZhbHVlcyA9IGMoImJsYWNrIiwicmVkIikpCnVubG9ja0JpbmRpbmcoImdncGxvdCIscGFyZW50LmVudihhc05hbWVzcGFjZSgiR0dhbGx5IikpKQphc3NpZ24oImdncGxvdCIsZ2dwbG90LHBhcmVudC5lbnYoYXNOYW1lc3BhY2UoIkdHYWxseSIpKSkKYGBgCgpgYGB7cn0KIyMgSW5wdXQgUEJTIG5vQ0wKaW5wdXRfUEJTX25vY2xfc2NhdHRlciA8LSBnZ3BhaXJzKG5vcm1fTWFzc1NwZWNEYXRhWyxJQkFRWzEzOjE0XV0sIAogICAgICAgIGxvd2VyID0gbGlzdChjb250aW51b3VzID0gd3JhcCgiY29yIiwgc2l6ZSA9IDcpKSwgCiAgICAgICAgdXBwZXIgPSBsaXN0KGNvbnRpbnVvdXMgPSAic21vb3RoIiksCiAgICAgICAgZGlhZyA9IGxpc3QoY29udGludW91cyA9ICJkZW5zaXR5RGlhZyIpKSArIHNjYXR0ZXJfdGhlbWUKCgojIyBJbnB1dCBQQlMgQ0wKaW5wdXRfUEJTX2NsX3NjYXR0ZXIgPC0gZ2dwYWlycyhub3JtX01hc3NTcGVjRGF0YVssSUJBUVsxNToxNl1dLCAKICAgICAgICBsb3dlciA9IGxpc3QoY29udGludW91cyA9IHdyYXAoImNvciIsIHNpemUgPSA3KSksIAogICAgICAgIHVwcGVyID0gbGlzdChjb250aW51b3VzID0gInNtb290aCIpLAogICAgICAgIGRpYWcgPSBsaXN0KGNvbnRpbnVvdXMgPSAiZGVuc2l0eURpYWciKSkgKyBzY2F0dGVyX3RoZW1lCgoKIyMgSG90UFRleCBQQlMgbm9DTApob3RwdGV4X1BCU19ub2NsX3NjYXR0ZXIgPC0gZ2dwYWlycyhub3JtX01hc3NTcGVjRGF0YVssSUJBUVs1OjZdXSwgCiAgICAgICAgbG93ZXIgPSBsaXN0KGNvbnRpbnVvdXMgPSB3cmFwKCJjb3IiLCBzaXplID0gNykpLCAKICAgICAgICB1cHBlciA9IGxpc3QoY29udGludW91cyA9ICJzbW9vdGgiKSwKICAgICAgICBkaWFnID0gbGlzdChjb250aW51b3VzID0gImRlbnNpdHlEaWFnIikpICArIHNjYXR0ZXJfdGhlbWUKCiMjIEhvdFBUZXggUEJTIENsCmhvdHB0ZXhfUEJTX2NsX3NjYXR0ZXIgPC0gZ2dwYWlycyhub3JtX01hc3NTcGVjRGF0YVssSUJBUVs3OjhdXSwgCiAgICAgICAgbG93ZXIgPSBsaXN0KGNvbnRpbnVvdXMgPSB3cmFwKCJjb3IiLCBzaXplID0gNykpLCAKICAgICAgICB1cHBlciA9IGxpc3QoY29udGludW91cyA9ICJzbW9vdGgiKSwKICAgICAgICBkaWFnID0gbGlzdChjb250aW51b3VzID0gImRlbnNpdHlEaWFnIikpICArIHNjYXR0ZXJfdGhlbWUKCgoKYGBgCgpgYGB7ciwgZmlnLndpZHRoPTEyLCBmaWcuaGVpZ2h0PTd9CmlucHV0X1BCU19ub2NsX3NjYXR0ZXIKaW5wdXRfUEJTX2NsX3NjYXR0ZXIKCmhvdHB0ZXhfUEJTX25vY2xfc2NhdHRlcgpob3RwdGV4X1BCU19jbF9zY2F0dGVyCgpgYGAKClBsb3RzIHdlcmUgc2F2ZWQgd2l0aDoKCmBgYHtyLCBldmFsPUZBTFNFLCBpbmNsdWRlPVRSVUV9Cmdnc2F2ZSgiZmlndXJlcy9pbnB1dF9QQlNfbm9jbF9zY2F0dGVyLnBkZiIsIHBsb3QgPSBpbnB1dF9QQlNfbm9jbF9zY2F0dGVyLCB1bml0cyA9ICJpbiIsIGhlaWdodCA9IDcsIHdpZHRoID0gMTIpCmdnc2F2ZSgiZmlndXJlcy9pbnB1dF9QQlNfY2xfc2NhdHRlci5wZGYiLCBwbG90ID0gaW5wdXRfUEJTX2NsX3NjYXR0ZXIsIHVuaXRzID0gImluIiwgaGVpZ2h0ID0gNywgd2lkdGggPSAxMikKCmdnc2F2ZSgiZmlndXJlcy9ob3RwdGV4X1BCU19ub2NsX3NjYXR0ZXIucGRmIiwgcGxvdCA9IGhvdHB0ZXhfUEJTX25vY2xfc2NhdHRlciwgdW5pdHMgPSAiaW4iLCBoZWlnaHQgPSA3LCB3aWR0aCA9IDEyKQpnZ3NhdmUoImZpZ3VyZXMvaG90cHRleF9QQlNfY2xfc2NhdHRlci5wZGYiLCBwbG90ID0gaG90cHRleF9QQlNfY2xfc2NhdHRlciwgdW5pdHMgPSAiaW4iLCBoZWlnaHQgPSA3LCB3aWR0aCA9IDEyKQoKYGBgCgpUaWR5IHVwOgoKYGBge3J9CnJtKGxpc3QgPSBscyhwYXR0ZXJuID0gIl9zY2F0dGVyIikpCmBgYAoKIyBQbG90cwoKClBsb3QgUkJQIGNsYXNzZXMKCmBgYHtyfQoKbWxpc3QgPC0gcmVhZC50YWJsZSgiUFRleFNhbG1vbmVsbGFfbWFzdGVyLnR4dCIsIHNlcCA9ICJcdCIsIGhlYWRlcj1UUlVFKQoKZ2dwbG90Mjo6Z2dwbG90KGRhdGEgPSBtbGlzdCwgYWVzKHggPSBpQkFRLlBUZXhfUEJTX21lYW4sIHkgPSBpQkFRLlBUZXhfUEJTX25vQ0xfbWVhbiwgY29sb3I9a25vd24uUk5BLmludGVyYWN0aW9uKSkgKwogIHNjYWxlX2NvbG91cl9tYW51YWwodmFsdWVzID0gYygib3JhbmdlIiwiZ3JleSIsInJlZCIpKSArIAogIHNjYWxlX2ZpbGxfbWFudWFsKHZhbHVlcyA9IGMoIm9yYW5nZSIsImdyZXkiLCJyZWQiKSkgKwogIGdlb21fcG9pbnQoc2l6ZSA9IDIuNSkgKwogIGdlb21fcG9pbnQoYWxwaGEgPSAwLjgpICsKICBnZW9tX2FibGluZShpbnRlcmNlcHQgPSAwKQoKI2VuciA8LSBnZ3Bsb3QyOjpnZ3Bsb3QoZGF0YSA9IG1saXN0LCBhZXMoeCA9IGlCQVEuUFRleF9QQlNfbWVhbiwgeSA9IGlCQVEuUFRleF9QQlNfbm9DTF9tZWFuLCBjb2xvcj1SaWJvc29tZSwgZ3JvdXA9Umlib3NvbWUpKSArCiMgIHNjYWxlX2NvbG91cl9tYW51YWwodmFsdWVzID0gYygiZ3JleSIsIm9yYW5nZSIpKSArIAojICBzY2FsZV9maWxsX21hbnVhbCh2YWx1ZXMgPSBjKCJncmV5Iiwib3JhbmdlIikpICsKIyAgZ2VvbV9wb2ludChzaXplID0gNSkgKwogICNnZW9tX3BvaW50KGFscGhhID0gMC44KSArCiMgIGdlb21fYWJsaW5lKGludGVyY2VwdCA9IDApCiAgCmVuciA8LSBnZ3Bsb3QyOjpnZ3Bsb3QoZGF0YSA9IG1saXN0LCBhZXMoeCA9IGlCQVEuUFRleF9QQlNfbWVhbiwgeSA9IGlCQVEuUFRleF9QQlNfbm9DTF9tZWFuLCBjb2xvcj1rbm93bi5STkEuaW50ZXJhY3Rpb24pKSArCiAgc2NhbGVfY29sb3VyX21hbnVhbCh2YWx1ZXMgPSBjKCJncmV5Iiwib3JhbmdlIikpICsgCiAgc2NhbGVfZmlsbF9tYW51YWwodmFsdWVzID0gYygiZ3JleSIsIm9yYW5nZSIpKSArCiAgZ2VvbV9wb2ludChzaXplID0gNSkgKwogICNnZW9tX3BvaW50KGFscGhhID0gMC44KSArCiAgZ2VvbV9hYmxpbmUoaW50ZXJjZXB0ID0gMCkKCgplbnIgPC0gZ2dwbG90Mjo6Z2dwbG90KGRhdGEgPSBtbGlzdCwgYWVzKHggPSBpQkFRLlBUZXhfUEJTX21lYW4sIHkgPSBpQkFRLlBUZXhfUEJTX25vQ0xfbWVhbiwgY29sb3I9a25vd24uUk5BLmludGVyYWN0aW9uKSkgKwogIHNjYWxlX2NvbG91cl9tYW51YWwodmFsdWVzID0gYygib3JhbmdlIiwiZ3JleSIsInJlZCIpKSArIAogIHNjYWxlX2ZpbGxfbWFudWFsKHZhbHVlcyA9IGMoIm9yYW5nZSIsImdyZXkiLCJyZWQiKSkgKwogIGdlb21fcG9pbnQoYWxwaGEgPSAwLjgpICsKICAgZ2VvbV9wb2ludChzaXplID0gNSkgKwogIGdlb21fYWJsaW5lKGludGVyY2VwdCA9IDApIAogICNnZW9tX2FibGluZShpbnRlcmNlcHQgPSAyLCBsdHkgPSAyKSArCiMgIGdlb21fYWJsaW5lKGludGVyY2VwdCA9IDQsIGx0eSA9IDMpICsKICAjZ2VvbV9hYmxpbmUoaW50ZXJjZXB0ID0gLTIsIGx0eSA9IDIpCmBgYApTYWZlIHBsb3QKYGBge3IsIGV2YWw9RkFMU0UsIGluY2x1ZGU9VFJVRX0KZ2dzYXZlKCJmaWd1cmVzL2ludGVuc2l0eV9lbnJpY2htZW50LnBkZiIsIHBsb3QgPSBlbnIsIHVuaXRzID0gImluIiwgaGVpZ2h0ID0gNywgd2lkdGggPSAxMikKCgpgYGAKCgoKCg==
