# Supplementary material for: Purification of cross-linked RNA-protein complexes by phenol-toluol extraction
Source: Nat Commun. 2019 Mar 1;10:990. doi: 10.1038/s41467-019-08942-3 (PMC6397201; doi:10.1038/s41467-019-08942-3)
Supplement: Supplementary file 13 — Reporting Summary [file 41467_2019_8942_MOESM13_ESM.pdf]

nature research

Corresponding author(s): Benedikt Beckmann

Last updated by author(s): Feb 2, 2019

# Reporting Summary

Nature Research wishes to improve the reproducibility of the work that we publish. This form provides structure for consistency and transparency in reporting. For further information on Nature Research policies, see Authors & Referees and the Editorial Policy Checklist.

## Statistics

For all statistical analyses, confirm that the following items are present in the figure legend, table legend, main text, or Methods section.

| n/a | Confirmed | |
|---|---|---|
| ☐ | ☒ | The exact sample size (*n*) for each experimental group/condition, given as a discrete number and unit of measurement |
| ☒ | ☐ | A statement on whether measurements were taken from distinct samples or whether the same sample was measured repeatedly |
| ☐ | ☒ | The statistical test(s) used AND whether they are one- or two-sided<br>*Only common tests should be described solely by name; describe more complex techniques in the Methods section.* |
| ☒ | ☐ | A description of all covariates tested |
| ☐ | ☒ | A description of any assumptions or corrections, such as tests of normality and adjustment for multiple comparisons |
| ☒ | ☐ | A full description of the statistical parameters including central tendency (e.g. means) or other basic estimates (e.g. regression coefficient) AND variation (e.g. standard deviation) or associated estimates of uncertainty (e.g. confidence intervals) |
| ☐ | ☒ | For null hypothesis testing, the test statistic (e.g. *F*, *t*, *r*) with confidence intervals, effect sizes, degrees of freedom and *P* value noted<br>*Give P values as exact values whenever suitable.* |
| ☒ | ☐ | For Bayesian analysis, information on the choice of priors and Markov chain Monte Carlo settings |
| ☒ | ☐ | For hierarchical and complex designs, identification of the appropriate level for tests and full reporting of outcomes |
| ☒ | ☐ | Estimates of effect sizes (e.g. Cohen's *d*, Pearson's *r*), indicating how they were calculated |

*Our web collection on statistics for biologists contains articles on many of the points above.*

## Software and code

Policy information about availability of computer code

| | |
|---|---|
| Data collection | No specific software was used for data collection. Raw data from devices (western blot images) was collected as described in the Methods section. |
| Data analysis | Data analysis was performed using R. Documented analysis is available as Supplementary Material as R notebooks. |

For manuscripts utilizing custom algorithms or software that are central to the research but not yet described in published literature, software must be made available to editors/reviewers. We strongly encourage code deposition in a community repository (e.g. GitHub). See the Nature Research guidelines for submitting code & software for further information.

## Data

Policy information about availability of data

All manuscripts must include a data availability statement. This statement should provide the following information, where applicable:

- Accession codes, unique identifiers, or web links for publicly available datasets
- A list of figures that have associated raw data
- A description of any restrictions on data availability

Data (RNASeq, Proteomics) have been made available in data repositories with the accession numbers as described in the respective paragraphs in the Methods section. Fig. 1,2,3,5 and 7 contain cropped images of western blots; the full images are provided in the Supplementary Information.

# Field-specific reporting

Please select the one below that is the best fit for your research. If you are not sure, read the appropriate sections before making your selection.

☒ Life sciences ☐ Behavioural & social sciences ☐ Ecological, evolutionary & environmental sciences

For a reference copy of the document with all sections, see nature.com/documents/nr-reporting-summary-flat.pdf

# Life sciences study design

All studies must disclose on these points even when the disclosure is negative.

| | |
|---|---|
| Sample size | Sample size was n = 3 (HEK293) or n = 2 (Salmonella) in the case of global RNP mapping. |
| Data exclusions | 1 RNA-Seq library (HEK293 input 0.015 J/cm2) was excluded from analysis due to poor library quality. |
| Replication | Western blot and PNK assays were performed at least three times independently. |
| Randomization | Not relevant to the study; the experimental setup (e.g. UV-irradiation vs. no irradiation) requires samples to come from the same original cell culture to ensure comparability. |
| Blinding | No blinding was performed. Instead, experiments were (partially) carried out by different lab members/co-authors. |

# Reporting for specific materials, systems and methods

We require information from authors about some types of materials, experimental systems and methods used in many studies. Here, indicate whether each material, system or method listed is relevant to your study. If you are not sure if a list item applies to your research, read the appropriate section before selecting a response.

## Materials & experimental systems

| n/a | Involved in the study | |
|---|---|---|
| ☐ | ☒ | Antibodies |
| ☐ | ☒ | Eukaryotic cell lines |
| ☒ | ☐ | Palaeontology |
| ☐ | ☒ | Animals and other organisms |
| ☒ | ☐ | Human research participants |
| ☒ | ☐ | Clinical data |

## Methods

| n/a | Involved in the study | |
|---|---|---|
| ☒ | ☐ | ChIP-seq |
| ☒ | ☐ | Flow cytometry |
| ☒ | ☐ | MRI-based neuroimaging |

## Antibodies

| | |
|---|---|
| Antibodies used | anti-HuR (proteintech, 11910-1-AP 0.42 µg/µL); anti-ACTB (proteintech, 66009-1-Ig 0.13 µg/µL); anti-FLAG (Sigma, F1804 1µg/µL); anti-Sxl-RBD4 (DHSB, antiSxl hybridome culture supernatant M114 1:20), FUS (ab- cam, ab124923), GAPDH (proteintech, 10494-1-AP), alpha- enolase (ENO1, proteintech,11204-1-AP), PTBP1 (abcam, ab133734), PABPC1 (proteintech, 10970-1-AP), Histone H3 (abcam, ab21054) |
| Validation | Commercially available antibodies were validated as described by the manufacturers; for anti-Sxl-RBD4 see Methods section |

## Eukaryotic cell lines

Policy information about cell lines

| | |
|---|---|
| Cell line source(s) | HEK293 cells were a gift from Markus Landthalers lab (MDC Berlin, Germany) |
| Authentication | HEK293 was not authenticated. Salmonella strains were tested by genomic DNA preparation and sequencing of single loci. |
| Mycoplasma contamination | HEK293 cell culture was routinely tested for Mycoplasma contamination. The results were negative. |
| Commonly misidentified lines (See ICLAC register) | No commonly misidentified cell lines were used. |

## Animals and other organisms

Policy information about studies involving animals; ARRIVE guidelines recommended for reporting animal research

| | |
|---|---|
| Laboratory animals | Mouse (C57BL/6) brain samples were a kind gift from M. Preußner (Laboratory Florian Heyd, Free University Berlin). |
| Wild animals | The study does not involve wild animals. |
| Field-collected samples | The study does not contain field-collected samples. |
| Ethics oversight | Ethics approval granted to the Heyd Laboratory at the Free University Berlin. Approval no.: T0311/13 |

Note that full information on the approval of the study protocol must also be provided in the manuscript.
