# Supplementary material for: Purification of cross-linked RNA-protein complexes by phenol-toluol extraction
Source: Nat Commun. 2019 Mar 1;10:990. doi: 10.1038/s41467-019-08942-3 (PMC6397201; doi:10.1038/s41467-019-08942-3)
Supplement: Supplementary file 2 — Peer Review File [file 41467_2019_8942_MOESM2_ESM.pdf]

Editorial Note: This manuscript has been previously reviewed at another journal that is not operating a transparent peer review scheme. This document only contains reviewer comments and rebuttal letters for versions considered at *Nature Communications*.

REVIEWERS' COMMENTS:

Reviewer #1 (Remarks to the Author):

The authors have addressed my comments well

Reviewer #2 (Remarks to the Author):

The Author have addressed all my comments in a satisfactory way. New data fully supports the authors' claims.

Reviewer #3 (Remarks to the Author):

The manuscript by Urdaneta and colleagues has been improved significantly after revision. This is an interesting work and deserves quick publication. I still have some concerns regarding specificity, and it can be seen, for example, in some Westerns (Figure 5h and i among others) that the background tends to be high. It might, thus, be worthy adding an extra note in the Discussion saying that this is a potential disadvantage compared to other methods based on nucleosides. I leave this to the discretion of the authors and editor. Nevertheless, the simplicity of the method and its application to cells difficult to label and potentially to tissues (even if the latter has not been fully assessed) makes the study timely and relevant.
